# A genome-wide association study of Chinese and English language phenotypes in Hong Kong Chinese children

**DOI:** 10.1038/s41539-024-00229-7

**Published:** 2024-03-27

**Authors:** Yu-Ping Lin, Yujia Shi, Ruoyu Zhang, Xiao Xue, Shitao Rao, Liangying Yin, Kelvin Fai Hong Lui, Dora Jue PAN, Urs Maurer, Kwong-Wai Choy, Silvia Paracchini, Catherine McBride, Hon-Cheong So

**Affiliations:** 1 School of Biomedical Sciences, The Chinese University of Hong Kong, Shatin, Hong Kong SAR, China; 2 Department of Bioinformatics, Fujian Key Laboratory of Medical Bioinformatics, School of Medical Technology and Engineering, Fujian Medical University, Fuzhou, China; 3 Key Laboratory of Ministry of Education for Gastrointestinal Cancer, School of Basic Medical Sciences, Fujian Medical University, Fuzhou, China; 4 Department of Psychology, Lingnan University, Tuen Mun, Hong Kong, China; 5 Wofoo Joseph Lee Consulting and Counselling Psychology Research Centre, Lingnan University, Tuen Mun, Hong Kong, China; 6 School of Humanities and Social Science, The Chinese University of Hong Kong (Shenzhen), Shenzhen, China; 7 Department of Psychology, The Chinese University of Hong Kong, Hong Kong SAR, China; 8 Brain and Mind Institute, The Chinese University of Hong Kong, Hong Kong SAR, China; 9 Centre for Developmental Psychology, The Chinese University of Hong Kong, Hong Kong SAR, China; 10 Department of Obstetrics and Gynecology, The Chinese University of Hong Kong, Hong Kong SAR, China; 11 School of Medicine, University of St Andrews, North Haugh KY16 9TF St Andrews, Scotland; 12 Department of Human Development and Family Science, Purdue University, West Lafayette, IN USA; 13 KIZ-CUHK Joint Laboratory of Bioresources and Molecular Research of Common Diseases, Kunming Institute of Zoology and The Chinese University of Hong Kong, Hong Kong SAR, China; 14 Department of Psychiatry, The Chinese University of Hong Kong, Hong Kong SAR, China; 15 CUHK Shenzhen Research Institute, Shenzhen, China; 16 Margaret K.L. Cheung Research Centre for Management of Parkinsonism, The Chinese University of Hong Kong, Shatin, Hong Kong SAR, China; 17 Hong Kong Branch of the Chinese Academy of Sciences Center for Excellence in Animal Evolution and Genetics, The Chinese University of Hong Kong, Hong Kong SAR, China

**Keywords:** Psychology, Human behaviour

## Abstract

Dyslexia and developmental language disorders are important learning difficulties. However, their genetic basis remains poorly understood, and most genetic studies were performed on Europeans. There is a lack of genome-wide association studies (GWAS) on literacy phenotypes of Chinese as a native language and English as a second language (ESL) in a Chinese population. In this study, we conducted GWAS on 34 reading/language-related phenotypes in Hong Kong Chinese bilingual children (including both twins and singletons; total *N* = 1046). We performed association tests at the single-variant, gene, and pathway levels. In addition, we tested genetic overlap of these phenotypes with other neuropsychiatric disorders, as well as cognitive performance (CP) and educational attainment (EA) using polygenic risk score (PRS) analysis. Totally 5 independent loci (LD-clumped at r^2^ = 0.01; MAF > 0.05) reached genome-wide significance (*p* < 5e-08; filtered by imputation quality metric Rsq>0.3 and having at least 2 correlated SNPs (r^2^ > 0.5) with *p* < 1e-3). The loci were associated with a range of language/literacy traits such as Chinese vocabulary, character and word reading, and rapid digit naming, as well as English lexical decision. Several SNPs from these loci mapped to genes that were reported to be associated with EA and other neuropsychiatric phenotypes, such as *MANEA* and *PLXNC1*. In PRS analysis, EA and CP showed the most consistent and significant polygenic overlap with a variety of language traits, especially English literacy skills. To summarize, this study revealed the genetic basis of Chinese and English abilities in a group of Chinese bilingual children. Further studies are warranted to replicate the findings.

## Introduction

Literacy and language skills are important for academic development in children. Learning difficulties (e.g., dyslexia) are common and may affect one’s school performance, leading to poorer work attainment and socioeconomic status, as well as decreased general well-being^1^. Multiple cognitive and language skills serve as the foundation for literacy and language development; these include, for example, working memory, rapid naming, and vocabulary knowledge^2^. A wide range of factors of environmental and genetic origins may also affect children’s literacy/language skills across different languages. Family, twin, and adoption studies have provided strong evidence that these complex cognitive and language traits and academic performance in young children are heritable^3–7^ and also highly polygenic^8,9^. However, the exact genes/variants involved in these traits are still not well understood, probably due to the complexity of the phenotypes and difficulty in gathering sufficient samples.

In recent years, several genome-wide association studies (GWAS) have been conducted on reading and language phenotypes in European populations. Several studies have focused on developmental dyslexia (DD) or high/low reading ability as a binary outcome, adopting a case-control study method^8,10–14^. Such study design may enable a larger sample size to be collected, but also has its shortcomings. Language and literacy skills cover a broad range of phenotypes, and dyslexia is also a highly heterogenous condition. The focus on a single binary outcome may limit our understanding into the biological mechanisms underlying different domains of language abilities. Other studies have investigated reading and language abilities as continuous traits^9,14–18^. However, one potential limitation is that many studies focused on a limited number or domain of phenotypes (e.g., rapid naming, word reading).

Given the relatively high heritability of literacy and language skills^19,20^, the genetic variants discovered thus far are still far from explaining the full genetic basis of these complex traits. In addition, most previous GWAS were conducted in European populations. However, the genetic architecture of language phenotypes may be different across ancestries, and some of the variants may be more readily discovered in other populations due to differences in allele frequency or LD (linkage disequilibrium) structure.

In addition, to our knowledge, very few GWAS have been published on children’s literacy/language skills of Chinese as a native language, or English as a second language (ESL) within Chinese. Given possible differences in mechanisms underlying Chinese and English literacy/language phenotypes, it is essential to specifically study the genetic basis of Chinese literacy/language phenotypes. In one recent GWAS on dyslexia^8^, several associated loci were also replicated in the Chinese Reading Study of reading accuracy and fluency; yet the primary GWAS was conducted predominantly on populations of European ancestry. After submission of this manuscript (and after this work was posted as a preprint on MedRxiv^21^), we also found a new GWAS on reading abilities in Chinese being published^22^. However, the current study covered a much wider range of phenotypes, and importantly, we also covered phenotypes related to ESL. Our sample was based on bilingual children with Cantonese as the native language, as opposed to children with (presumably) Putonghua as the native language in Wang et al.^22^.

In view of the limitations of previous studies, here we conducted GWAS and related bioinformatics analyses on a comprehensive panel of 34 literacy/language-related phenotypes in a Hong Kong (HK) Chinese population. The wide coverage enables a systematic and unbiased analysis of a variety of phenotypes. Since this is among the first study of Chinese- and ESL-related phenotypes in a Chinese population, and the genetic bases of such phenotypes are still largely unknown, it is our objective to explore a wider range of traits to maximize the chance of discovery, and to provide a starting point and important reference for future studies.

To summarize, in this study we investigated how genetics is associated with individual differences in Chinese and English reading and writing. We performed association tests at the single-variant, gene, and pathway levels, and employed transcriptome-wide association studies (TWAS) to explore how genotype-imputed expression changes affect the phenotypes. In addition, we tested potential associations between these complex cognitive traits with other neuropsychiatric disorders, as well as cognitive performance and educational attainment by polygenic risk score (PRS) analysis. To the best of our knowledge, this is among the first GWAS conducted on a comprehensive range of Chinese-language phenotypes together with ESL-related phenotypes in a Chinese population.

## Results

In this study, we recruited 1048 Chinese children aged 5–12 years from Hong Kong, including 274 MZ twin pairs, 350 DZ twin pairs and 424 singletons. All children were typically developing with Cantonese as their first language and English as their second language. We conducted GWAS on 34 reading/language-related phenotypes. Association tests were performed at the single-variant, gene, and pathway levels. In addition, we tested genetic overlap of these phenotypes with other language-related or neuropsychiatric traits using polygenic risk score (PRS) analysis.

### Single-variant associations

Quantile-quantile plots (QQ-plots) with lambda (*λ*) were constructed for each trait with and without rank-based inverse normal transformation (RINT). We found that the QQ-plots were very similar for most phenotypes with or without the transformation, except for four [Backward digit span (BDS_Total), Chinese Vocabulary - Receptive Vocabulary (CVA_Total), Chinese digit rapid naming (CDRAN_Mean) and English digit rapid naming (EDRAN_Mean)] (see Supplementary Fig. 3 and Supplementary Data 1). For these 4 traits, subsequent analyses were based on the RINT-transformed values. Based on the updated QQ-plots, all four traits showed no evidence of inflated false positives after the transformation. Manhattan plots for all traits are shown in Supplementary Data 2.

In SNP-based analysis, a total of 5 independent loci (LD-clumped at r^2^ threshold 0.01; MAF threshold = 0.05) reached genome-wide (GW) significance (*p* < 5e-08), filtered by imputation quality score (Rsq) > 0.3 and having at least 2 correlated SNPs (r^2^ > 0.5) with *p* < 1e-3 (Supplementary Data 3/Table 1). Here the check for correlated significant SNPs was performed to further reduce the risk of false positives, and the check was performed using the default settings of LD-clumping in PLINK. For the purpose of replication analysis, we also provided data on GW-significant SNPs with MAF > 0.01 (Supplementary Data 19); however, given the small sample size, we recommend that the top SNPs with low MAF should be viewed very cautiously, and confirmation in independent samples is required.

**Table 1:** Results of the SNP-based association analysis

Phenotype	CHR	BP	SNP	A1	A2 (Effect allele)	*P*	MAF	Rsq	Genotyped	Closest gene	S0001	FDR-adjusted *P*
ELD_Total	6	95643248	rs6905617	C	A	3.29E-09	0.352	0.52	Imputed	*MANEA-AS1(-364.7* *kb)*	43	2.82E-03
CCR_Total	9	115640979	rs56024259	G	A	1.53E-08	0.124	0.98	Imputed	*SLC46A2(-0.22* *kb)*	7	3.07E-02
CDRAN_Mean	12	94529190	rs3847795	A	C	1.73E-08	0.173	0.94	Imputed	*PLXNC1(-13.31* *kb)*	4	8.95E-02
CVB_Total	4	57573275	rs4865143	T	C	4.97E-08	0.071	0.80	Imputed	*HOPX(* + *25.4* *kb)*	27	9.04E-02
CWR_Total	4	57573275	rs4865143	T	C	3.61E-08	0.071	0.80	Imputed	*HOPX(* + *25.4* *kb)*	15	1.55E-01

For full results please refer to Supplementary Data 3. A2 is the effect allele. Results are sorted by *P*-value. *MAF* minor allele frequency, *Rsq* R-squared (imputation quality metric), *BP* base pair (position of the SNP); S0001, number of clumped SNPs (SNPs in LD) with *p* < 1e-3. Only SNPs with S0001 > = 2 and MAF > 0.05 are shown. FDR-adjusted *P*, false-discovery rate-adjusted *P*-value by the Benjamini-Hochberg method.

The significant loci were associated with a variety of language/literacy phenotypes such as Chinese vocabulary, character and word reading, and digit rapid naming, as well as English lexicon decision. Note that one locus was associated with two (correlated) phenotypes, namely rs4865143 which was associated with both CWR_total and CVB_total (*r* = 0.63). In addition, we also searched the top-listed genes in GWAS catalog for associations with other phenotypes (especially neuropsychiatric traits) in previous studies. Please refer to Supplementary Data 12 for details.

The most significant association was observed for rs6905617 (*p* = 3.29E-09) with English Lexical Decision (ELD); the SNP is located close to *MANEA* (−382.1 kb) and *MANEA-AS1* (−364.7 kb). As for Chinese-related traits, we discovered one significant locus for CCR, CWR, CDRAN, and CVB respectively (filtered by MAF > 0.05; see Table 1 and Supplementary Data 3.3).

We also calculated the lambda-GC (genomic inflation factor) for each untransformed trait and there was no evidence of inflation (Supplementary Data 9; largest lambda-GC = 1.0255, 29/34 traits showed lambda-GC < 1.02).

### Association analyses between genetically predicted expression and phenotypes

We evaluated the association between genetically regulated expression (GRex) and phenotypes across multiple brain regions by S-Predixcan. We used pre-computed weights provided by the authors (available at https://predictdb.org/), derived from an elastic net regression model with transcriptome reference data from GTEx(v7). The most significant associations were observed for *DUS3L*, which showed significant associations (FDR < 0.05) with EWR_Total in four brain regions including amygdala, caudate basal, cerebellar hemisphere and putamen (Table 2 and Supplementary Data 4.1). The top 20 association results from S-PrediXcan are presented in Table 2 (see also Supplementary Data 4 for the top 100 associations).

**Table 2:** Top 20 S-Predixcan results after correction of multiple testing

Phenotype^a^	Tissue_name	Gene	Zscore	*P*	FDR-adjust *P*^b^
EWR_Total	Brain_Amygdala	*DUS3L*	4.81	1.52E-06	4.18E-02
EWR_Total	Brain_Caudate_basal_ganglia	*DUS3L*	4.72	2.35E-06	4.18E-02
EWR_Total	Brain_Putamen_basal_ganglia	*DUS3L*	4.69	2.76E-06	4.18E-02
EWR_Total	Brain_Cerebellar_Hemisphere	*DUS3L*	4.6	4.20E-06	4.77E-02
EWR_Total	Brain_Hypothalamus	*AC005523.3*	4.37	1.23E-05	1.12E-01
EMA_Total	Brain_Frontal_Cortex_BA9	*ZNF585B*	−4.67	3.07E-06	1.30E-01
CVB_Total	Brain_Cerebellum	*BNIPL*	4.58	4.70E-06	2.13E-01
EWR_Total	Brain_Frontal_Cortex_BA9	*DUS3L*	4.13	3.60E-05	2.72E-01
RC_MC	Brain_Cortex	*RP11-508N22.12*	−4.52	6.18E-06	2.81E-01
EWR_Total	Brain_Nucleus_accumbens_basal_ganglia	*DUS3L*	3.99	6.58E-05	4.27E-01
EDC_Total	Brain_Cerebellum	*GTF3C5*	4.41	1.03E-05	4.66E-01
ELD_Total	Brain_Cerebellum	*FAM86B2*	−4.37	1.24E-05	5.62E-01
EMA_Total	Brain_Cerebellum	*KIAA0355*	4.12	3.80E-05	5.79E-01
EMA_Total	Brain_Substantia_nigra	*CHL1*	4.1	4.11E-05	5.79E-01
EMA_Total	Brain_Cerebellar_Hemisphere	*TSEN15*	−3.81	1.41E-04	7.48E-01
EMA_Total	Brain_Hippocampus	*HNRNPCP1*	−3.84	1.25E-04	7.48E-01
EMA_Total	Brain_Nucleus_accumbens_basal_ganglia	*RP11-521C20.2*	−3.92	8.98E-05	7.48E-01
EMA_Total	Brain_Putamen_basal_ganglia	*RASA4*	−3.91	9.22E-05	7.48E-01
EMA_Total	Brain_Spinal_cord_cervical_c-1	*C20orf202*	−3.84	1.22E-04	7.48E-01
EVA_Total	Brain_Amygdala	*RP11-178F10.3*	−3.94	8.18E-05	8.33E-01

^a^Please refer to Table 10 for abbreviations of the phenotype.

^b^FDR-adjust *P*: Calculated by the R.program p.adjust using Benjamini-Hochberg procedure (BH).

Furthermore, we employed S-MulTiXcan to improve power by combining evidence of differential expression across all brain regions. We observed 248 significant gene-level associations (with FDR < 0.05) by this approach and identified the best representative brain region (the region showing the strongest single-tissue association). The top 20 results are presented in Table 3 and full results in Supplementary Data 5. We highlight a few findings here. The most significant S-Multixcan association was observed for gene *HSD3B7* with EVA_total (Hydroxy-Delta-5-Steroid Dehydrogenase, 3 Beta- And Steroid Delta- Isomerase 7; best brain region, Brain_Cortex; FDR-adjusted *p* = 9.55E-20). *HSD3B7* was also associated with other English literacy phenotypes, such as EVB, EVK, EVD, EDRAN and EWR. For Chinese literacy skills, the most significant association was observed for gene *SEMA6C* (Semaphorin 6C; best brain region, Brain_Cerebellar_Hemisphere; FDR-adjusted *p* = 2.77E-12) with CVB_Total.

**Table 3:** Top 20 S-Multixcan results after correction of multiple testing

Phenotype^a^	T_i_best^b^	Gene	P_i_best^c^	FDR.adjust *P*^*d*^
EVA_Total	Brain_Cortex	*HSD3B7*	1.71E-03	9.55E-20
ELD_Total	Brain_Hypothalamus	*RP11-497H16.2*	9.72E-06	4.55E-14
EVK_Total	Brain_Caudate_basal_ganglia	*HSD3B7*	5.12E-03	3.68E-13
CVB_Total	Brain_Cerebellar_Hemisphere	*SEMA6C*	3.77E-04	2.77E-12
CDICT_Total	Brain_Caudate_basal_ganglia	*LINC00638*	4.47E-03	6.79E-12
CVA_Total	Brain_Nucleus_accumbens_basal_ganglia	*PIF1*	8.76E-03	6.91E-12
EVB_Total	Brain_Cortex	*HSD3B7*	1.16E-02	1.69E-11
CWR_Norm	Brain_Hypothalamus	*RP11-497H16.2*	4.14E-04	9.80E-11
CWR_Total	Brain_Hypothalamus	*RP11-497H16.2*	9.01E-04	1.66E-09
CCR_Total	Brain_Hypothalamus	*RP11-497H16.2*	3.53E-05	2.96E-09
EVD_Total	Brain_Caudate_basal_ganglia	*HSD3B7*	8.07E-03	7.52E-09
EWR_Total	Brain_Cortex	*HSD3B7*	1.40E-02	1.71E-08
ELRAN_Mean	Brain_Hypothalamus	*RP11-497H16.2*	1.93E-04	3.30E-08
ELRAN_Mean	Brain_Nucleus_accumbens_basal_ganglia	*BAK1P1*	3.91E-03	4.34E-08
EDRAN_Mean	Brain_Nucleus_accumbens_basal_ganglia	*ZNF565*	2.00E-02	5.33E-08
EDRAN_Mean	Brain_Cortex	*HSD3B7*	2.01E-02	2.34E-07
EDICT_Total	Brain_Anterior_cingulate_cortex_BA24	*MYO6*	3.35E-04	3.77E-07
COM_Score	Brain_Cerebellum	*RBM8A*	8.38E-02	4.39E-07
CLD_Total	Brain_Caudate_basal_ganglia	*OXCT2P1*	3.87E-04	6.56E-07
ELRAN_Mean	Brain_Nucleus_accumbens_basal_ganglia	*CYP2E1*	7.51E-03	6.70E-07

^a^Please refer to Table 10 for abbreviations of the phenotype.

^b^T_i_Best: name of best single-tissue S-Predixcan association.

^c^P_i_Best: best p-value of single tissue S-Predixcan association.

^d^FDR-adjust *P*: FDR-adjusted *p*-value of the overall *p*-value output by S-Multixcan. FDR was calculated by the R program p.adjust using the Benjamini-Hochberg procedure (BH).

### Gene-based tests

We also conducted gene-based analyses using MAGMA, which aggregates SNP-level associations into a gene-level statistic. The top 20 significant results are presented in Table 4 and full results in Supplementary Data 6. We highlight several genes within the top-10 list here.

**Table 4:** Top 20 gene-based results (Magma) after correction of multiple testing

phenotype^a^	Description	Gene	CHR	ZSTAT	*P*	FDR.adjust *P*^*b*^
PureC_Total	potassium voltage-gated channel subfamily C member 1	*KCNC1*	11	6.03	8.18E-10	1.49E-05
CVD_Total	general transcription factor IIIC subunit 1	*GTF3C1*	16	5.41	3.24E-08	5.90E-04
EWR_Total	cation channel sperm associated auxiliary subunit delta	*CATSPERD*	19	5.16	1.22E-07	2.22E-03
EIS_Total	solute carrier family 2 member 12	*SLC2A12*	6	5.16	1.25E-07	2.27E-03
EIS_Total	radial spoke head component 1	*RSPH1*	21	5.01	2.74E-07	2.49E-03
CVB_Total	mitogen-activated protein kinase 10	*MAPK10*	4	5.09	1.76E-07	3.20E-03
MS_Total	regulatory factor X8	*RFX8*	2	4.96	3.57E-07	3.25E-03
MS_Total	small lysine rich protein 1	*SMKR1*	7	5.05	2.24E-07	3.25E-03
CVK_Total	general transcription factor IIIC subunit 1	*GTF3C1*	16	5.09	1.81E-07	3.30E-03
EVB_Total	cation channel sperm associated auxiliary subunit delta	*CATSPERD*	19	5.03	2.42E-07	4.40E-03
CVB_Total	BCL2 interacting protein like	*BNIPL*	1	4.86	5.87E-07	5.34E-03
EVB_Total	cilia and flagella associated protein 65	*CFAP65*	2	4.84	6.46E-07	5.89E-03
BDS_Total	transmembrane serine protease 13	*TMPRSS13*	11	4.96	3.48E-07	6.33E-03
EVK_Total	cilia and flagella associated protein 65	*CFAP65*	2	4.83	6.95E-07	1.27E-02
EWR_Total	caveolae associated protein 2	*CAVIN2*	2	4.46	4.19E-06	1.39E-02
EWR_Total	Morf4 family associated protein 1 like 1	*MRFAP1L1*	4	4.49	3.57E-06	1.39E-02
EWR_Total	biogenesis of lysosomal organelles complex 1 subunit 4	*BLOC1S4*	4	4.44	4.57E-06	1.39E-02
EWR_Total	proline rich 22	*PRR22*	19	4.54	2.81E-06	1.39E-02
EWR_Total	dihydrouridine synthase 3 like	*DUS3L*	19	4.57	2.43E-06	1.39E-02
EDRAN_Mean	ankyrin repeat domain 50	*ANKRD50*	4	4.80	7.76E-07	1.41E-02

^a^Please refer to Table 10 for abbreviations of the phenotype.

^b^FDR-adjust *P*: Calculated by the R.program p.adjust using Benjamini-Hochberg procedure (BH).

The most significant association was observed for *KCNC1* (potassium voltage-gated channel subfamily C member 1) with PureC_total (FDR corrected *p* = 1.49E-5). For English-related phenotypes, the most significant association was identified for gene *CATSPERD* (cation channel sperm associated auxiliary subunit delta) with EWR_Total (FDR corrected *p* = 2.22E-03); the same gene was also associated with EVB_Total (FDR corrected *p* = 4.40E-03). Two genes showed associations with EIS_Total, namely *SLC2A12* (solute carrier family 2 member 12; FDR corrected *p* = 2.27E-03) and *RSPH1* (radial spoke head component 1; FDR-corrected *p* = 2.49E-03).

As for Chinese literacy skills, *GTF3C1* (general transcription factor IIIC subunit 1) was associated with CVD_Total (FDR corrected *p* = 5.90E-04) and CVK_Total (FDR corrected *p* = 3.03E-3); *MAPK10* (mitogen-activated protein kinase 10) was associated with CVB_Total (FDR corrected *p* = 3.20E-03). As for morphosyntactic skills in Chinese, the genes *SMKR1*(small lysine rich protein 1; FDR corrected *p* = 3.25E-03) and *RFX8* (regulatory factor X8; FDR corrected *p* = 3.25E-03) were associated with MS_Total.

Quantile-quantile plots (QQ-plots) with lambda-GC (*λ*) were constructed for each trait based on gene-based test results. There is no evidence of inflated false positives, with most *λ* < 1 and only two traits having *λ* > 1 (1.02 and 1.07) (see Supplementary Figs. 4, 5).

### Pathway enrichment analysis

To reveal relevant functional pathways, we conducted a self-contained gene-set analysis in GAUSS, testing 10679 canonical pathway and gene ontology (GO) gene sets from the MSigDB database. Full results with FDR < 0.2 are shown in Supplementary Data 7.1 and 7.2. Tables 5, 6 summarize the pathway and GO analyses results with FDR-corrected *p* < 0.05. We also present the top two pathways and GO terms enrichment for every trait in Supplementary Data 7.3 and 7.4.

**Table 5:** Significant gene ontology (GO) enrichment results (by GAUSS) after correction of multiple testing (FDR < 0.05)

GeneSet	Pvalue	Phenotype	FDR adjust *P*^*a*^
GO_SPHINGOLIPID_MEDIATED_SIGNALING_PATHWAY	6.88E-09	CDICT_Total	4.07E-05
GO_GLYCEROPHOSPHOLIPID_CATABOLIC_PROCESS	6.40E-08	PureC_Total	3.78E-04
GO_PROTON_TRANSPORTING_V_TYPE_ATPASE_COMPLEX	1.20E-07	CWR_Norm	7.13E-04
GO_ALCOHOL_TRANSMEMBRANE_TRANSPORTER_ACTIVITY	2.38E-07	RC_MC	1.41E-03
GO_DIVALENT_INORGANIC_ANION_HOMEOSTASIS	5.74E-07	PureC_Total	1.70E-03
GO_CELLULAR_ANION_HOMEOSTASIS	2.25E-06	PureC_Total	4.44E-03
GO_BIOACTIVE_LIPID_RECEPTOR_ACTIVITY	2.13E-06	CDICT_Total	6.29E-03
GO_ATP_HYDROLYSIS_COUPLED_TRANSMEMBRANE_TRANSPORT	2.22E-06	EWR_Total	1.31E-02
GO_LYMPHANGIOGENESIS	7.35E-06	CDICT_Total	1.45E-02
GO_ORGANIC_HYDROXY_COMPOUND_TRANSMEMBRANE_TRANSPORTER_ACTIVITY	4.90E-06	RC_MC	1.45E-02
GO_POSITIVE_REGULATION_OF_VASODILATION	2.00E-05	PureC_Total	1.48E-02
GO_POSITIVE_REGULATION_OF_B_CELL_DIFFERENTIATION	2.00E-05	PureC_Total	1.48E-02
GO_POSITIVE_REGULATION_OF_BLOOD_CIRCULATION	2.00E-05	PureC_Total	1.48E-02
GO_NEURON_PROJECTION_GUIDANCE	2.00E-05	PureC_Total	1.48E-02
GO_POLYSACCHARIDE_BINDING	2.00E-05	PureC_Total	1.48E-02
GO_MONOVALENT_INORGANIC_ANION_HOMEOSTASIS	3.00E-05	PureC_Total	1.97E-02
GO_REGULATION_OF_MITOCHONDRIAL_FISSION	1.53E-05	CDICT_Total	2.27E-02
GO_RESPONSE_TO_NERVE_GROWTH_FACTOR	1.08E-05	EWR_Total	2.43E-02
GO_PROTON_TRANSPORTING_TWO_SECTOR_ATPASE_COMPLEX_CATALYTIC_DOMAIN	1.64E-05	EWR_Total	2.43E-02
GO_PROTON_TRANSPORTING_V_TYPE_ATPASE_COMPLEX	1.35E-05	EWR_Total	2.43E-02
GO_LIGAND_GATED_CHANNEL_ACTIVITY	4.14E-06	EDRAN_Mean	2.45E-02
GO_HYDROGEN_TRANSPORT	3.00E-05	EWR_Total	2.96E-02
GO_VACUOLAR_PROTON_TRANSPORTING_V_TYPE_ATPASE_COMPLEX	3.00E-05	EWR_Total	2.96E-02
GO_RNA_CAP_BINDING_COMPLEX	5.34E-06	CDC_Total	3.16E-02
GO_POSITIVE_REGULATION_OF_MITOCHONDRIAL_FISSION	3.00E-05	CDICT_Total	3.29E-02
GO_DIOL_METABOLIC_PROCESS	4.00E-05	CDICT_Total	3.29E-02
GO_LYMPH_VESSEL_MORPHOGENESIS	5.00E-05	CDICT_Total	3.29E-02
GO_LYMPH_VESSEL_DEVELOPMENT	5.00E-05	CDICT_Total	3.29E-02
GO_VENOUS_BLOOD_VESSEL_DEVELOPMENT	4.00E-05	CDICT_Total	3.29E-02
GO_G_PROTEIN_COUPLED_PURINERGIC_NUCLEOTIDE_RECEPTOR_SIGNALING_PATHWAY	5.61E-06	CVK_Total	3.32E-02
GO_ORGANIC_HYDROXY_COMPOUND_TRANSPORT	5.00E-05	RC_MC	3.70E-02
GO_NERVE_DEVELOPMENT	5.00E-05	RC_MC	3.70E-02
GO_BLOOD_VESSEL_REMODELING	2.09E-05	RC_MC	3.70E-02
GO_KINETOCHORE	5.00E-05	RC_MC	3.70E-02
GO_CONDENSED_CHROMOSOME_CENTROMERIC_REGION	5.00E-05	RC_MC	3.70E-02
GO_CONDENSED_NUCLEAR_CHROMOSOME_CENTROMERIC_REGION	5.00E-05	RC_MC	3.70E-02
GO_DETECTION_OF_LIGHT_STIMULUS	6.00E-05	RC_MC	3.94E-02
GO_STEROID_BINDING	7.00E-05	PureC_Total	4.14E-02
GO_G_PROTEIN_COUPLED_PURINERGIC_NUCLEOTIDE_RECEPTOR_SIGNALING_PATHWAY	7.58E-06	CVD_Total	4.48E-02
GO_WNT_SIGNALING_PATHWAY_CALCIUM_MODULATING_PATHWAY	2.31E-05	CCR_Total	4.56E-02
GO_LOCOMOTORY_EXPLORATION_BEHAVIOR	2.00E-05	CCR_Total	4.56E-02
GO_RNA_CAP_BINDING_COMPLEX	2.00E-05	CCR_Total	4.56E-02
GO_LIPASE_ACTIVATOR_ACTIVITY	8.00E-05	CDICT_Total	4.73E-02
GO_DRUG_TRANSPORTER_ACTIVITY	8.07E-06	CVA_Total	4.78E-02
GO_POSITIVE_REGULATION_OF_B_CELL_ACTIVATION	9.00E-05	PureC_Total	4.84E-02

Please refer to Table 10 for abbreviations of the phenotypes. Full descriptions of each gene-set can be found by looking up the pathway names at https://www.gsea-msigdb.org/gsea/msigdb/.

^a^FDR-adjust *P*: Calculated by the R.program p.adjust using Benjamini-Hochberg procedure (BH).

**Table 6:** Significant Pathway enrichment results (GAUSS) after correction of multiple testing (FDR < 0.05)

GeneSet	Pvalue	Phenotype	FDR adjust *P*^*a*^
REACTOME_RNA_POL_III_TRANSCRIPTION	3.36E-08	WO_Total	1.60E-04
BIOCARTA_P35ALZHEIMERS_PATHWAY	3.41E-07	EWR_Total	1.62E-03
REACTOME_P2Y_RECEPTORS	3.94E-07	CVK_Total	1.88E-03
REACTOME_KINESINS	7.07E-07	BDS_Total	3.37E-03
STOSSI_RESPONSE_TO_ESTRADIOL	3.04E-06	RC_MC	1.45E-02
IGLESIAS_E2F_TARGETS_DN	4.29E-06	CWR_Norm	2.04E-02
REACTOME_P2Y_RECEPTORS	5.25E-06	CVD_Total	2.50E-02
PID_S1P_META_PATHWAY	9.02E-06	CDICT_Total	3.88E-02
GOLUB_ALL_VS_AML_DN	1.63E-05	CDICT_Total	3.88E-02
BIOCARTA_AKAPCENTROSOME_PATHWAY	2.00E-05	CCR_Total	4.76E-02
BANDRES_RESPONSE_TO_CARMUSTIN_MGMT_48HR_UP	2.00E-05	CCR_Total	4.76E-02
LIM_MAMMARY_LUMINAL_PROGENITOR_UP	2.00E-05	EWR_Total	4.76E-02

Please refer to Table 10 for abbreviations of the phenotypes. Full descriptions of each gene-set can be found by looking up the pathway names at https://www.gsea-msigdb.org/gsea/msigdb/.

^a^FDR-adjust *P*: Calculated by the R.program p.adjust using Benjamini-Hochberg procedure (BH).

In pathway-based enrichment analysis of Chinese comprehension skills, the strongest association was observed for WO_Total with the Reactome RNA polymerase III transcription pathway (FDR corrected *p* = 1.60E-04). The second most significant association was observed for EWR_Total with the ‘Deregulation of CDK5 in Alzheimers Disease’ pathway (BioCarta) (FDR corrected *p* = 1.62E-03). Other pathways with the top five included the P2Y receptors (associated with CVK_total) and kinesins pathways (associated with BDS_total). GAUSS has also identified a collection of corresponding core genes (CS) for each pathway (Supplementary Data 7.1).

In gene ontology (GO) enrichment analysis, the most significant enrichment was observed between CDICT_Total and sphingolipid-medicates signaling pathway (FDR corrected *p* = 4.07E-05). Other GO gene-sets within the top 5 (with respect to lowest p-values) included glycerophospholipid catabolic process, proton-transporting V-type ATPase complex, alcohol transmembrane transporter activity and divalent inorganic anion homeostasis. They were associated with PureC_total, CWR_norm, RC_MC and PureC_total, respectively. With regards to English literacy skills, the GO gene-set ‘ATP hydrolysis coupled cation transmembrane transport’ (FDR corrected *p* = 1.31E-02) showed the strongest association (with EWR_total). GAUSS selected 14 core genes for the gene set, in which one of them, *BLOC1S4*, was individually and significantly associated with EWR_Total (Supplementary Data 7.2).

### PRS analysis with neuropsychiatric phenotypes, cognitive performance (CP), and education attainment (EA)

Here we briefly describe several significant or suggestive findings (with FDR-corrected *p* < = 0.1) in PRS analysis. The most consistent PRS associations were observed for EA and CP. For example, PRS constructed from GWAS of EA was significantly associated with 20 out of 34 traits (at FDR < 0.1 at one or more *p* thresholds), while PRS of CP was significantly associated with 16 traits (FDR < 0.1), using the clumping and thresholding (C + T) approach. Another approach SBayesR also produced similar results, with 25 traits showing significant associations with PRS of CP and 10 traits showing associations with PRS of EA (at FDR < 0.1). All associations were in the expected direction (i.e., higher EA and CP PRS associated with better reading/language abilities).

Interestingly, these associations appeared to be more consistent across English reading/literacy phenotypes compared to Chinese phenotypes. We aggregated the p-values from SBayesR analysis of EA and CP across all Chinese- and English-related traits respectively (*p*-value aggregation performed using Simes/ACAT tests). PRS of EA was significantly associated with English-related phenotypes (Simes *p* = 3.34e-4; ACAT *p* = 1.90e-4) but not with Chinese-related phenotypes (Simes *p* = 3.55e-1; ACAT *p* = 1.45e-1). As for PRS of CP, it was significantly associated with both English-related (Simes *p* = 5.43e-4; ACAT *p* = 3.08e-4) and Chinese-related phenotypes (Simes *p* = 3.03e-3; ACAT *p* = 1.92e-3), yet the level of statistical significance was stronger for English-related traits.

As for other neuropsychiatric traits, using SbayesR, PRS of ASD was significantly associated with various language phenotypes, such as reading comprehension (RC), English vocabulary, English word reading and dictation, and several other traits. The C + T approach mainly showed associations with RC. Higher ASD PRS were associated with better reading abilities. PRS of other psychiatric disorders did not show consistent evidence of association with most language phenotypes, although there were a few results with FDR < 0.1.

**Fig. 1:**
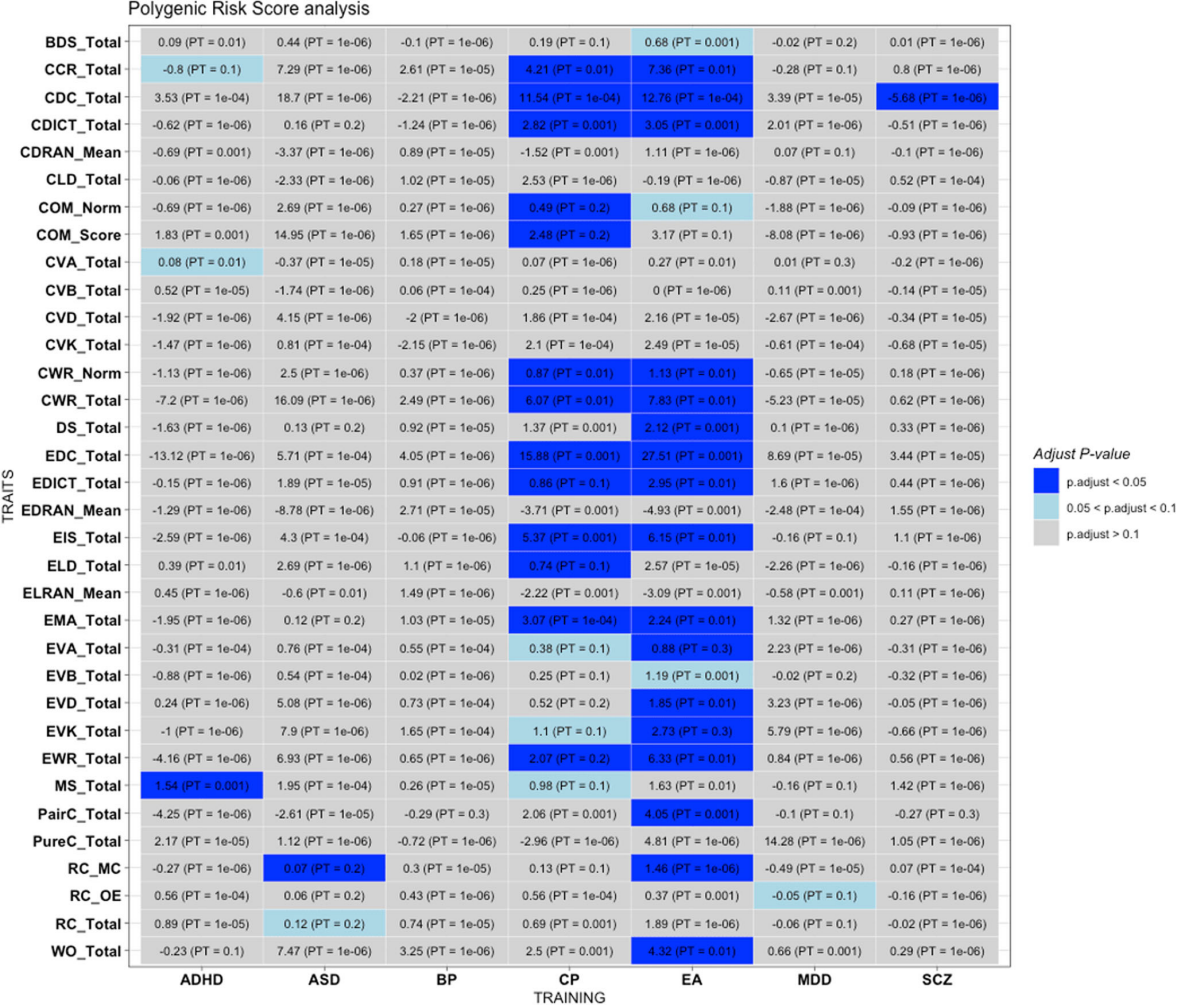
Results of polygenic risk score (PRS) analysis on the 34 language-related phenotypes analyzed in this study, with PRS constructed from external GWAS data of different neuropsychiatric disorders/traits (training set). The following neuropsychiatric disorders/traits were included: attention deficit hyperactivity disorder (ADHD), autism spectrum disorders (ASD), Education attainment (EA), cognitive performance (CP), schizophrenia (SCZ), bipolar disorder (BP) and major depressive disorder (MDD). In the heatmap, for each PRS analysis, we select the result with the lowest FDR-adjusted *p*-value (p.adjust), and show the regression coefficient in the graph. The PRS represent the average risk allele score per non-missing SNP. PT: the optimal *p*-value threshold at which the most significant association was observed.

**Fig. 2:**
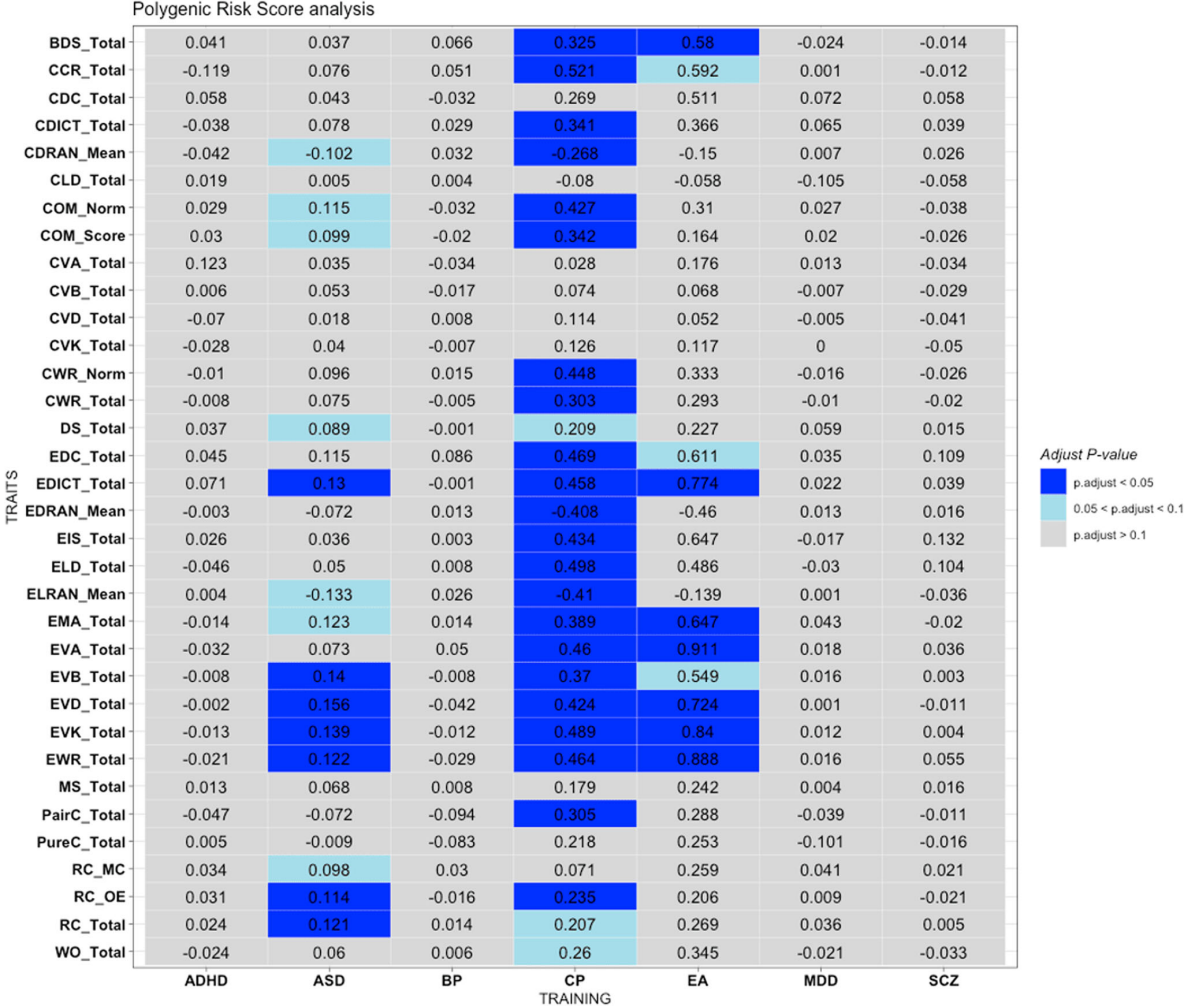
Results of polygenic risk score (PRS) analysis using SBayesR on the 34 language-related phenotypes analyzed in this study, with PRS constructed from external GWAS data of different neuropsychiatric disorders/traits (training set). Note that SBayesR assumes a mixture model on the SNP effect sizes, and does not require choosing p-value thresholds. Please also refer to the legend of Fig. 1.

We present in Fig. 1 the results of PRS analysis at the best pthres cutoff; Fig. 2 shows the results from SBayesR. The full results for the (C + T) approach across all pthres can be found in Supplementary Data 8, while the results for SBayesR are reported in Supplementary Data 15.

### Testing for genetic overlap with other GWAS on dyslexia/reading abilities

#### SNP-set and gene-set analysis based on top SNPs/genes reported from Doust et al.^8^ and Wang et al.^22^

For the SNP-based analysis, the identified susceptibility SNPs for CVB_Total showed significant overlap with those identified for dyslexia in an independent GWAS^8^. There is also some evidence for overlap for CWR_Norm. Based on another smaller study by Wang et al.^22^, significant overlap were observed for CVB_Total, CVK_Total, ELS_Total, EMA_Total (Table 7). Full results are presented in Supplementary Data 13. The above analysis results were consistent across the Simes and ACAT tests.

**Table 7:** Testing for genetic overlap with Doust et al. and Wang et al. on reading/language phenotypes, based on SNP-based test statistics

(1) GWAS by Doust et al.			
Phenotype	*P* < 5e-08	*P* < 5e-06	*P* < 1-06
CVB_Total	3.11E-04	6.58E-04	6.46E-04
CWR_Norm	3.62E-02	7.66E-02	7.52E-02
(2) GWAS by Wang et al.			
**Phenotype**		***P*** < **5e-06**	***P*** < **1e-05**
CVB_Total		2.51E-02	5.01E-02
CVK_Total		2.12E-02	4.23E-02
EIS_Total		8.10E-03	1.62E-02
EMA_Total		2.98E-02	2.83E-02

The above is based on the Simes test. Only traits showing significant results are shown above. Full results (including results from Simes and ACAT tests) are presented in Supplementary Data 13.

For SNP-set analysis based on the GWAS by Wang et al., since the number of SNPs with available data is small, we aggregated the top SNPs across all eight phenotypes studied by Wang et al.

For details of the statistical test, please refer to the main text. Briefly, for SNP-set analysis, we first identified top SNPs (defined by p-values smaller than predefined cutoffs) from two independent GWAS datasets on dyslexia and reading abilities. Then we extracted the same SNP-set from our data, and performed the Simes test and ACAT test to examine whether the SNP-set as a whole was significantly associated with our studied traits.

In a similar manner, we also performed gene-set analysis based on the top genes identified in Doust et al.^8^ and Wang et al.^22^. Significant results (using Simes test) are presented in Table 8 and full results in Supplementary Data 14. Here we mainly report the results from the Simes test, as ACAT produced similar findings. As shown in Supplementary Data 14, based on top genes from the dyslexia GWAS^8^, significant gene-set analysis results were observed across multiple reading/language phenotypes. A total of 10 phenotypes were significant (*p* < 0.05) across at least 2 *p*-value thresholds, and 6 phenotypes showed significant aggregate *p*-value (the *p*-value aggregating evidence from multiple *p* thresholds using Simes test). These phenotypes include CVB_Total, CVD_Total, CVK_Total, CDICT_Total, CCR_Total and MS_Total. ACAT tests showed concordant results but seemed to be more powerful, with 11 phenotypes having significant aggregate *p*-values. For the top genes identified from the other Chinese GWAS^22^, we also observed significant results for various phenotypes (6 with Simes test and 8 with ACAT), suggesting an overlap of genetic signals.

**Table 8:** Testing for genetic overlap with Doust al. and Wang et al. on reading/language phenotypes, based on gene-based test statistics

(1) GWAS by Doust et al.							
Phenotype	*P* < 0.05	*P* < 0.01	*P* < 0.001	*P* < 1e-04	*P* < 1e-05	*P* < 1e-06	Aggregate_P
CCR_Total	8.73E-02	4.18E-02	2.98E-02	1.47E-02	8.63E-03	2.74E-02	4.40E-02
CDICT_Total	1.88E-02	9.01E-03	6.69E-02	4.56E-02	2.68E-02	1.73E-02	3.76E-02
CVB_Total	6.60E-04	3.16E-04	1.97E-01	9.68E-02	5.70E-02	3.67E-02	1.90E-03
CVD_Total	1.22E-04	1.12E-01	4.76E-02	4.54E-01	4.39E-01	2.83E-01	7.31E-04
CVK_Total	6.81E-04	7.73E-02	6.58E-02	6.15E-01	7.24E-01	4.66E-01	4.09E-03
DS_Total	2.03E-01	1.94E-01	8.26E-02	4.06E-02	2.39E-02	1.54E-02	7.18E-02
EVB_Total	2.69E-02	1.29E-02	5.78E-01	2.84E-01	2.23E-01	1.56E-01	7.73E-02
MS_Total	8.41E-04	4.03E-04	5.66E-01	4.00E-01	2.42E-01	1.56E-01	2.42E-03
PairC_Total	6.51E-02	3.12E-02	2.65E-02	1.41E-01	1.20E-01	7.74E-02	9.36E-02
WO_Total	4.49E-02	2.15E-02	1.45E-01	4.23E-01	2.73E-01	3.05E-01	1.29E-01
(2) GWAS by Wang et al.							
**Phenotype**	***P*** < **1e-05**						
CLD_Total	4.89E-03						
CVK_Total	8.15E-03						
EMA_Total	8.44E-03						
CVB_Total	1.26E-02						
MS_Total	1.52E-02						
RC_OE	4.75E-02						

The above is based on the Simes test. Only traits showing significant results across at least two p-value thresholds (for the first study) and or at *p* < 1e-5 (for the second study) are shown. Full results (including results from Simes and ACAT tests) are presented in Supplementary Data 14.

For gene-set analysis based on the GWAS by Wang et al., since the number of genes with available data is small, we aggregated the top genes across all eight phenotypes studied by Wang et al.

For details of the statistical test, please refer to the main text. Briefly, we first extracted top genes from the external datasets with (gene-based) p-values smaller than predefined cutoffs, then extracted the same set of genes from our sample. We then tested whether the gene-set (as a whole) was significantly associated with the studied phenotypes. This replication analysis was conducted under various *p*-value cutoffs (*p* = 0.05, 1e-2, 1e-3, 1e-4, 1e-5 and 1e-6) (for the Doust et al. study). For the other GWAS, only one threshold was used, as only the summary gene-based statistics with *p* < 1e-5 were available.

#### Testing for genetic dependence using full GWAS summary statistics from GenLang

The results are presented in Table 9 and Supplementary Data 16. We observed that multiple Chinese and ESL-related phenotypes showed genetic overlap with the reading/language traits from the GenLang study^9^, as evidenced by the Hoeffding’s test of independence. Out of the 170 pairs (34 traits from HK sample x 5 traits from ref. 9) of reading/language phenotypes, 42 achieved nominal significance (*p* < 0.05) in the test for genetic dependence, while 22 achieved FDR-adjusted *p*-values < 0.1 (mostly with ‘word reading’ from the GenLang sample). The top pairs of traits showing the most significant genetic dependence were DS_Total, EIS_Total and CVA_Total with ‘word reading’ of the GenLang sample.

**Table 9:** Testing for genetic dependence with the GenLang sample (Eising et al.), using full GWAS summary statistics and the Hoeffding’s test of independence (results with FDR adjusted *p* < 0.1 are shown)

Trait A	Trait B	Scaled statistic	*p*-value	FDR-adjusted *p*
BDS_Total	Word Reading	1.663	2.04E-02	**4.63E-02**
CDC_Total	Spelling	2.696	4.24E-03	8.75E-02
CLD_Total	Word Reading	2.406	6.55E-03	**2.02E-02**
CVA_Total	Word Reading	5.853	4.31E-05	**4.88E-04**
CVB_Total	Word Reading	2.247	8.34E-03	**2.36E-02**
CVK_Total	Word Reading	1.852	1.53E-02	**3.70E-02**
CWR_Total	Word Reading	1.122	4.84E-02	8.65E-02
DS_Total	Word Reading	6.382	2.03E-05	**4.47E-04**
EDRAN_Mean	Word Reading	1.216	4.15E-02	7.85E-02
EIS_Total	Word Reading	6.199	2.63E-05	**4.47E-04**
ELD_Total	Word Reading	4.428	3.33E-04	**2.83E-03**
ELRAN_Mean	Word Reading	2.665	4.44E-03	**1.68E-02**
ELRAN_Mean	Spelling	2.566	5.14E-03	8.75E-02
EVA_Total	Word Reading	1.228	4.07E-02	7.85E-02
EVD_Total	Word Reading	2.975	2.79E-03	**1.36E-02**
EWR_Total	Word Reading	2.137	9.85E-03	**2.58E-02**
MS_Total	Word Reading	3.298	1.74E-03	**9.84E-03**
PureC_Total	Word Reading	1.038	5.55E-02	9.43E-02
RC_MC	Word Reading	2.774	3.77E-03	**1.60E-02**
RC_OE	Word Reading	2.433	6.29E-03	**2.02E-02**
RC_Total	Word Reading	3.786	8.48E-04	**5.77E-03**
WO_Total	Word Reading	1.460	2.81E-02	5.98E-02
WO_Total	Phoneme awareness	2.967	2.83E-03	9.62E-02

Trait A comes from the Hong Kong sample, while trait B is from the study by Eising et al. Scaled statistic: the test statistic rescaled for a standard null distribution (please refer to the R package “independence” for details). FDR adjusted-*p* < 0.05 are in bold and those between 0.05 and 0.1 are in italics. FDR adjustment was performed with stratification by trait B.

As a further exploratory analysis, we also evaluated the correlations of the effect sizes of top SNPs from HK and GenLang samples. In general, we did not find significant correlations that pass multiple testing corrections, but the SNP effect sizes for CCR_total showed a positive Pearson correlation with spelling (Supplementary Data 18). The lack of significant correlations for example could be due to our limited sample size, and/or heterogeneity across studies. On the other hand, we note that the above is a preliminary measure of the correlation of genetic signals (Supplementary Notes), and more rigorous methods such as LDSC shall be attempted to assess genetic correlation in future studies with larger sample sizes.

#### Polygenic risk score analysis

**Fig. 3:**
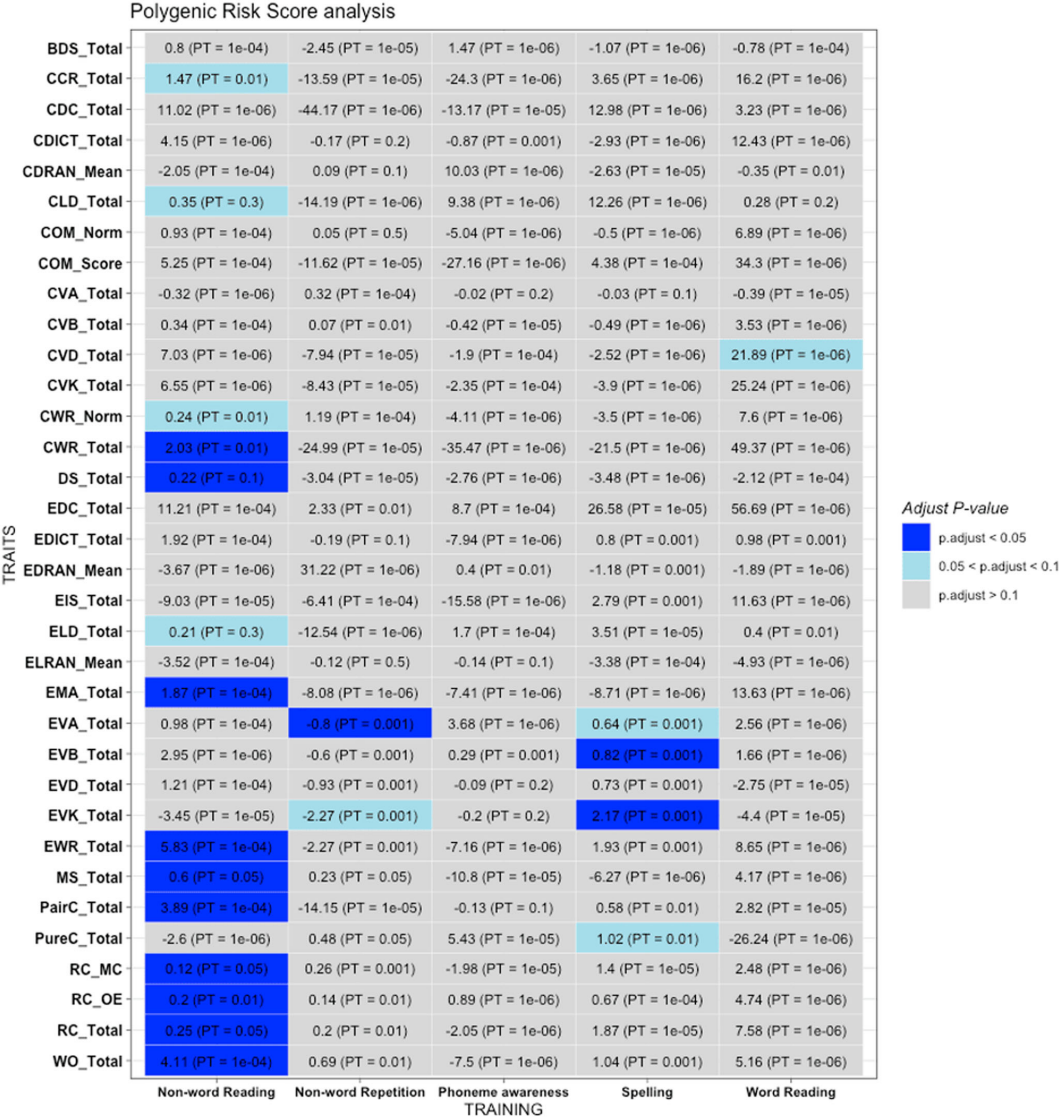
Results of polygenic risk score (PRS) analysis on the 34 language-related phenotypes analyzed in this study, with PRS constructed from external GWAS data of reading and language-related traits from Eising et al. The following traits were included: word reading, nonword reading, spelling, phoneme awareness, and nonword repetition. In the heatmap, for each PRS analysis, we select the result with the lowest FDR-adjusted *p*-value (p.adjust), and show the regression coefficient in the graph. The PRS represent the average risk allele score per non-missing SNP. PT: the optimal *p*-value threshold at which the most significant association was observed.

Based on PRS constructed from the GWAS by Eising et al.^9^, we found that PRS of ‘non-word reading’ was significantly and positively associated (at FDR < 0.05) with multiple reading/language phenotypes of our study, especially those related to Chinese language (e.g. Chinese word reading (CWR), discourse skills, morphosyntax, reading comprehension and word order). Please refer to Supplementary Data 17 and Fig. 3 for the detailed results. We also observed significant and positive associations of the PRS of ‘spelling’ with English vocabulary knowledge. Significant results were primarily observed with the clumping and thresholding approach. SBayesR in general did not return significant findings (Supplementary Fig. 6), however, we observed several associations of language phenotypes from the HK sample with ‘non-word reading’, with FDR < 0.2.

We did not find significant results surviving multiple testing when PRS was constructed from the dyslexia GWAS^8^. However, the directions of effects are consistent with prior expectations. At a *p*-value thres1hold of 1.31e-6, a total of 25 (out of 34) traits showed concordant directions of effect (i.e. higher dyslexia PRS associated with poorer reading abilities; *p* = 0.0045, one-sided binomial test); at a p-threshold of 5e-8, 29/34 traits showed concordant directions of effect; *p* = 1.928e-5).

As for PRS analysis based on the GWAS from Wang et al.^22^, in general there are few significant results after FDR correction. We note that the sample size of the above GWAS is relatively modest (*N* = 2284), and since only SNPs with *p* < 1e-5 are available, there are few SNPs (<10) left for PRS construction after standard LD-clumping. As such, this analysis is considered exploratory, and the results should be interpreted with the above limitations in mind. To highlight one notable finding, PRS constructed from morphological awareness (MA) measure from Wang et al. showed some evidence of association with English MA in our sample (*p* = 0.0018), with the same direction of expected effect. The full results are presented in Supplementary Data 11.

## Discussion

In this study, we attempted to uncover the genetic basis of a comprehensive range of cognitive, literacy, and language-related phenotypes of Chinese (as a native language) and English (as a second language). To gain insights into the genetic architecture of the above phenotypes, we carried out a GWAS within a group of Hong Kong children. To the best of our knowledge, this is among the first GWAS to explore the genetic basis of a comprehensive set of literacy- and language-related traits in both Chinese and English in a Chinese population. Compared to the previous GWAS on language traits (see introduction), this study also covers the widest range of phenotypes, enabling a finer resolution into the genetic architecture of language abilities.

One distinct feature of this study is that we selected the subjects drawn from a large longitudinal project in Hong Kong, a city with a unique linguistic background due to its geographical location and political history^23^. As such, our study is among the first to assess the genetics of language and literacy skills of bilingual (Chinese and English) children systematically.

Here we highlight several genes associated with literacy/language phenotypes based on our SNP- or gene-based analysis. For English literacy skills, the most significant association was observed for a SNP close to *MANEA* and *MANEA-AS1* (rs6905617) with English lexical decision. Interestingly, by a search of the GWAS catalog, we found that a variant in *MANEA* showed tentative association with general cognitive ability in a previous GWAS (*p* = 5e-6)^24^ ; genetic variants in *MANEA-AS1* may also be associated with behavioral inhibition^25^. Another gene of interest in *PLXNC1*; variants in this gene have been reported to be associated (at *p* < 1e-5) with multiple neuropsychiatric phenotypes such as major depression^26^, Lewy body dementia^27^, brain shape (segment 15 and 79)^28^ and neuroticism^29^. We also briefly highlight a few genes with corresponding SNPs having FDR < 0.1 (and MAF > 0.05) in GWAS analysis (see Supplementary Data 3.2). A block of variants in *STXBP6* were associated with CVB_total. Syntaxin-binding protein 6 (STXBP6) is an essential component of the SNAP receptor (SNARE) complex and plays an important role in synaptic transmission and neuronal vesicle trafficking; mutations of genes encoding the SNARE proteins are associated with various neurological disorders^30,31^. Common variants in *STXBP6* were reported to be linked to cortical surface area^32^ and rate of cognitive decline in Alzheimer’s disease^33^. Variants in *NRXN3* (Neurexin-3) were also associated with CVB_total in our study. Of note, variants in this gene were found to be associated with word reading^18^ and education attainment^34^ in recent GWAS. Neurexin-3 plays crucial roles in synapse development and functions and neurotransmission^35^. Another gene of interest is *MAP1B*, which was associated with BDS_total. SNPs in this gene were linked to educational attainment^34^ and brain morphology^36^.

Several gene-based tests reached a significant level after FDR correction for reading and spelling measures. The most significant gene from MAGMA was *KCNC1*, which encodes a subunit of the KV3 voltage-gated K^+^ channels. Mutations in this gene were associated with a range of neurological disorders including epilepsy and also intellectual disability and cognitive decline in some patients^37–39^. In terms of Chinese literacy skills, the most significant association signal was observed for gene *GTF3C1* (General Transcription Factor lllC Subunit 1) with CVD_Total. *GTF3C1* has been widely investigated on its interactive connections to other genes; for example, it is involved in networks pathologically related to neurodegeneration and Alzheimer’s disease^40–42^. *GTF3C1* is also involved in regulation of rearrangement of neuronal nuclear architecture following neuronal excitation^43^. Of note, the nuclear architecture plays an important role in neural development and function^44^. *CHL1* was another gene implicated from S-PrediXcan analysis, and variants in this gene were reported to show association with education attainment^45^ and mathematics abilities^45^.

In addition, our results showed that *SLC2A12* was associated with English comprehension skills. *SLC2A12* encodes GLUT12, a glucose transporter. It has been reported that amyloid-beta increases GLUT12 protein expression in the brain in mouse models, implicating an important role of this transporter in Alzheimer disease^46^ and cognitive functioning.

We discovered that several language/literacy phenotypes were associated with PRS of psychiatric disorders, cognitive performance and educational attainment. Our results were consistent with previous studies that have demonstrated shared genetic factors among childhood intelligence, educational attainment, and literacy skills.

For example, Luciano et al. (2017)^47^ showed that PRS of word reading, general reading and spelling, as well as non-word repetition, were positively associated with educational attainment (college/university degree versus none), income and verbal-numerical cognitive test results. Moreover, in a GWAS by Price et al.^14^, substantial genetic overlap was found between word reading and number of years of education (*R*^2^ = 0.07, *P* = 4.91 × 10^−48^) and intelligence score (*R*^2^ = 0.18, *P* = 7.25 × 10^−181^) in a population-based sample. In a recent study by Gialluisi et al.^48^, risk of developmental dyslexia was significantly associated with PRS of EA and intelligence. In addition, in another large-scale GWAS on dyslexia^8^, negative genetic correlation of dyslexia with intelligence and education attainment was reported. Combined with our current findings, these results provide evidence to support a partially shared genetic etiology among literacy skills, cognitive measures, and educational outcomes. On the other hand, it is interesting to note that the polygenic scores of EA and CP appeared more strongly associated with English language phenotypes (English as a second language) than their Chinese counterparts, which is a novel finding to our knowledge. The above finding also suggests there may be differences underlying the genetic basis of Chinese and English literacy skills.

Another interesting finding was that ASD PRS was associated with reading/language-related phenotypes, with higher PRS associated with better reading/language abilities. Notably, several genetic studies on ASD have observed positive genetic correlation or positive PRS associations between ASD and CP or EA^49–51^. However, a recent study^49^ also showed significant heterogeneity of polygenic associations across ASD subtypes. Regarding EA, in the above study, PRS of EA was significantly and positively associated with childhood autism and Asperger’s syndrome, but not for atypical autism, or the group of unspecified/other pervasive developmental disorders (PDD). Similar pattern of associations was observed for PRS of intelligence. In another study^52^, it was found that language problems related to ASD was positively associated with dyslexia, however, ASD-related inflexibility was associated with a reduced odds of dyslexia. Here we observed a positive association of ASD PRS with language traits, which may warrant further studies in independent samples, ideally with more refined PRS of different ASD subtypes or symptom domains. However, we note that the significant associations are primarily observed using SBayesR but not the conventional C + T approach, and our sample size is modest, as such the findings may need to be further replicated in other studies.

Here we have performed genetic overlap/replication and PRS analyses based on several other relevant GWAS on dyslexia and language phenotypes, namely Doust et al.^8^, Wang et al.^22^ and Eising et al.^9^. As detailed above, we observed some evidence of genetic overlap between these datasets and our HK sample. Most significant findings of genetic overlap by PRS were observed with the study by Eising et al.^9^. However, there was weaker evidence of genetic overlap with the other two samples, and not all reading/language phenotypes showed significant PRS associations. We highlight possible reasons for discrepancies in genetic findings below.

Firstly, for the analysis with the dyslexia GWAS, a major limitation is that only the 10,000 top SNPs were available (highest *p* ~ 1.31e-6 after LD-clumping). As language and literacy phenotypes are complex traits which are likely highly polygenic, inclusion of a smaller number of SNPs might limit the power to detect associations. As a reference, for PRS analyses of EA and CP, the most significant associations were in general observed at more relaxed *p*-value cutoffs (>0.001).

Secondly, since the dyslexia GWAS is mainly based on a European population^8^, differences in genetic findings could be attributed to ethnic differences. Another study by Eising et al.^9^ is also primarily based on European samples. It is increasingly recognized that PRS constructed from one ethnic group often have poorer performance in other ethnicities. The deterioration in performance may even occur across ethnic subgroups within the same ancestry (e.g. European ancestry)^53^. Differences in other environmental or genetic backgrounds may also affect effect sizes of genetic variants. A recent study also revealed that even within the UK-Biobank sample, prediction accuracy varies for various traits depending on socio-economic status, age and sex of the subjects^54^. Differences in the background of subjects may be present across the current and other studies.

In addition, while dyslexia was the target phenotype in the above-cited GWAS^8^, we focused on a variety of literacy and language-related phenotypes. Also, we focused on Chinese language phenotypes and phenotypes associated with ESL, as compared to dyslexia in a mostly European and English-speaking population. The differences in phenotypes may contribute to different variants/genes being detected. Moreover, the 23andMe sample^8^ largely depends on self-reported diagnosis of dyslexia, which may be subject to error and heterogeneity.

We also highlight a few other differences between our current study and Eising et al.^9^ (GenLang study). Eising et al.^9^ focused on five English reading phenotypes and discovered a GW-significant loci for word reading, while in this study we observed significant signals mainly for Chinese-related phenotypes (and English Lexical decision, which was not directly studied in Eising et al.). Also, the GenLang study is a meta-analysis which may be more heterogeneous (e.g. phenotypic heterogeneity across different sub-samples) than a single study, and the age range was also wider (up to 18 years old). On the other hand, compared to the large meta-analysis by GenLang, although we identified several novel loci, we consider our findings more preliminary and tentative given the limit of sample size. We cannot exclude the possibility of some false positives and independent replications are needed.

As for another GWAS on reading traits in Chinese^22^, the power to detect significant PRS associations may be limited due to the modest sample size, and that only limited number of top SNPs are available for modeling. Other reasons stated above, such as heterogeneity of the study sample and phenotypes studied, may also play a role.

There are several strengths of our study. First, to the best of our knowledge, this is among the first GWAS to investigate the genetic basis of a wide range of both Chinese and English literacy- and language-related skills in a Chinese population. Importantly, as reading and language comprehension are highly complex traits, here we performed detailed phenotyping to decipher the genetic basis of various different domains of these skills. On the other hand, previous studies largely followed another research strategy by focusing on a limited range of language phenotypes or binary outcomes. While it is also possible to only focus on a few selected phenotypes (e.g., those with higher heritability, or by other criteria), such choice of phenotypes may inevitably be arbitrary, and one may still discover variants of biological importance for a trait with lower heritability. In addition, the SNP-based heritability, or the extent to which common variants contribute to a trait, is unknown for most phenotypes studied here. To enable a more comprehensive and unbiased examination of the genetic architecture of language/literacy-related traits, we have included a wide range of phenotypes in the current study. We also employed the FDR approach to account for multiple testing.

To gain deeper insights into the biological basis of the studied traits, we not only performed standard SNP-based tests but also gene-based (MAGMA, S-PrediXcan, S-MulTiXcan) and pathway-based analysis (GAUSS). This ‘multi-level’ approach helps to bridge the gap between SNP associations and biological mechanisms, thus enhancing our knowledge and understanding of reading and language. In addition to studying the associations between phenotypes and genetic factors, we performed PRS analysis to study the overlap of included phenotypes with other neuropsychiatric traits, which could provide insight into the genetic architecture of language-related traits.

Our study also has a few limitations. Our study is based on a Hong Kong Chinese sample (under a bilingual environment). It remains uncertain whether the genetic findings from the current study can be generalized to other populations. Further studies in other populations with different genetic and language backgrounds may be warranted. In a similar vein, the GWAS summary statistics of CP, EA and other psychiatric disorders were primarily derived from Europeans (due to lack of relevant data from Chinese populations), which may attenuate the genetic overlap with the studied phenotypes in our Chinese sample. Nevertheless, several studies (on other complex traits) have shown that genetic variants and PRS from Europeans may still be transferrable to Chinese^55,56^, albeit with possibly weaker predictive power. Besides, here we employed the 1000-Genomes as the reference for imputation, following the findings from Lin et al.^57^ that satisfactory imputation performance in Chinese could be achieved using this panel. In Lin et al.’s report, the mean imputation r^2^ in two Chinese cohorts were at or above ~0.7 for SNPs having MAF > 1%, and were even better for higher MAF. At the time of this analysis, most established imputation servers (e.g. Michigan Imputation Server) does not contain Chinese-specific reference panels. Note that we also reported the imputation quality score (r^2^) for all reported variants for easy reference and have removed variants with low imputation quality (r^2^ < 0.3).

In this study, we performed extensive and deep phenotyping covering most domains of Chinese and English literacy- and language-related skills. This GWAS covers the widest range of language phenotypes to date. However, as a compromise, our sample size is relatively modest and statistical power may be insufficient to detect variants of small effects. In addition, given that we only performed genetic analysis in a single sample and a number of phenotypes were probably studied for the first time (e.g. most phenotypes on Chinese language/literacy), we emphasize that further replications in other samples are required. The modest sample size may also contribute to negative heritability estimates by LDSC; future studies of SNP-based heritability using larger samples are warranted. In addition, this study focused on the contribution of common variants; rare variant association was not our focus and further sequencing studies may be warranted. In addition, while we have performed further gene-based and pathway-based bioinformatics analyses, the findings are based on statistical associations and will require further experimental validations.

In summary, we conducted one of the first GWAS on a comprehensive range of phenotypes on *both* Chinese and English abilities in a HK Chinese (Cantonese-speaking) population. We discovered a few novel genetic loci that may underlie these traits, and revealed genes and pathways that may be associated, although we stress that further replications are warranted owing to the modest sample size. We believe our work will be an important starting point and reference for further studies into the biological and genetic basis of language abilities, and ultimately such knowledge will be useful for the development of better treatment for children with specific reading disabilities.

## Methods

### Participants and phenotypes studied

The participants were Hong Kong Chinese-English bilingual twins and singletons, recruited through kindergarten and primary schools in Hong Kong. All children were typically developing with Cantonese as their mother language and English as their second language. The participants’ ages ranged between 5 to 12 years old at the time of assessment. A total of 1048 children were recruited for this study, including 274 MZ subjects (137 pairs), 350 DZ subjects (175 pairs) and 424 singletons. Zygosity determination on twin pairs was based on the genotyped small tandem repeat (STR) markers using Quantitative Fluorescence Polymerase Chain Reaction (QF-PCR)^58^. Singleton children were selected from the same schools as those twin pairs. Parental written informed consent for all the participants was obtained before testing. Children completed a series of cognitive and literacy-related tasks in Chinese and English either in a laboratory setting, their school, or their home by trained research assistants.

For details of the tasks and phenotypes, please refer to the Supplementary Notes. Briefly, a total of 34 phenotypes were included (Table 10), covering a wide range of literacy- and language-related skills. All tasks were finished in a given order that had been predetermined. Except the three measures on rapid automatized naming (RAN), a higher score indicates better literacy skills. A correlation matrix of all phenotypes is presented in Supplementary Fig. 1.

**Table 10:** Overview of phenotypes included in the study

Variable	Variable Label
BDS_Total	Backward Digit Span
CCR_Total	Chinese Character Reading
CDC_Total	Chinese Delayed Copying
CDICT_Total	Chinese Dictation
CDRAN_Mean	Chinese Digit Rapid Naming
CLD_Total	Chinese Lexical Decision
COM_Score	Chinese 1 min Word Reading Adjusted Total Score
COM_Norm	Chinese 1 min Word Reading Scaled Score
CVA_Total	Chinese Vocabulary - Receptive Vocabulary (10 items)
CVB_Total	Chinese Vocabulary - Expressive Vocabulary (12 items)
CVD_Total	Chinese Vocabulary - Vocabulary Definition (26 items)
CVK_Total	Chinese Vocabulary Knowledge (48 items; sum of CVA, CVB and CVK)
CWR_Total	Chinese Word Reading Raw Score
CWR_Norm	Chinese Word Reading Scaled Score
DS_Total	Chinese Discourse Skills
EDC_Total	English Delayed Copying
EDICT_Total	English Dictation
EDRAN_Mean	English Digit Rapid Naming
EIS_Total	English Invented Spelling
ELD_Total	English Lexical Decision
ELRAN_Mean	English Letter Rapid Naming
EMA_Total	English Morphological Awareness - Written Test
EVA_Total	English Vocabulary - Receptive Vocabulary (15 items)
EVB_Total	English Vocabulary - Expressive Vocabulary (15 items)
EVD_Total	English Vocabulary - Vocabulary Definition (15 items)
EVK_Total	English Vocabulary Knowledge (45 items; sum of EVA, EVB and EVK)
EWR_Total	English Word Reading Total Score
MS_Total	Morphosyntax in Chinese
PairC_Total	Pair Cancellation
PureC_Total	Pure Copying of Unfamiliar Scripts
RC_MC	Chinese Reading Comprehension - Multiple Choice
RC_OE	Chinese Reading Comprehension - Open End
RC_Total	Chinese Reading Comprehension - Total
WO_Total	Chinese Word Order

### Genotype quality control (QC) and imputation

Three groups of subjects, including monozygotic (MZ) twins, dizygotic twins (DZ), and singletons, were genotyped. Based on previous studies^59^, reducing the MZ pairs to singletons leads to a loss of statistical power. It has also been shown that including both MZ twins in the genetic analysis does not lead to an inflation of type I error (when relatedness is accounted for) but can improve power^59^. We therefore followed ref. 59 and included both MZ twins in our GWAS. Monozygosity was confirmed by QF-PCR as described above, and only one member of each MZ pair was genotyped. The other MZ twin was assumed to share identical genotypes. We employed the Human Infinium OmniZhongHua-8 v1.3 Beadchip from Illumina for genotyping.

Quality control (QC) was performed by PLINK-1.9 on each dataset separately before merging. We removed those SNPs which deviated from Hardy–Weinberg equilibrium (HWE, *P* < 1E-5), with Minor Allele Frequency (MAF) < 1%, missingness per individual (MIND) > 10%, and missingness per marker (GENO) > 10%. After QC, 911178 SNPs and 1046 individuals were kept for further analysis, including 274 MZ subjects (59 male pairs, 78 female pairs), 349 DZ subjects (39 male pairs, 37 female pairs, 1 member of a female pair and 98 opposite-sex pairs), as well as 423 singletons (218 males, 205 females).

Following QC, variant-level imputation was performed by the Michigan Imputation Server based on “Mininac”^60^. The imputation was based on the reference panel 1000 Genomes (1000 G) Phase 3 v5, as previous studies reported satisfactory performance of imputation in Chinese based on the 1000 G panel^57^. The imputed data were converted into a binary dosage file by the program “DosageConverter” (https://genome.sph.umich.edu/wiki/DosageConvertor). Imputed variants with INFO score (R-squared) > 0.3 (12,475,316 SNPs) were retained.

### Genome-wide association study (GWAS)

GWAS of all phenotypes was conducted through a univariate linear mixed model in GEMMA (http://github.com/genetic-statistics/GEMMA). We included age and sex as fixed-effects covariates. The genetic relationship matrix (GRM) was included as a random effect to account for relatedness between subjects. This approach also controls for population stratification. We tested for the association of allelic dosages with phenotypes. An MAF threshold of 0.05 was employed for the SNP-based analysis. We considered *p* < 5e-8 as the genome-wide significance threshold.

Although multiple phenotypes were studied, our primary objective was to explore and prioritize genetic variants for further studies, and a further Bonferroni correction to penalize the number of phenotypes tested may be too conservative for this purpose. Instead, we employed the false discovery rate (FDR) approach to control for multiple testing. FDR controls the expected *proportion* of false positives among the findings declared to be significant. This approach has been argued to be a more reasonable methodology as it ‘adaptively’ considers the data instead of imposing a direct penalty for the number of hypotheses tested, and the FDR approach has also been widely used in genomic studies^61^.

FDR was calculated separately for each trait, for all SNP- and gene-based analyses (see below). It is worth noting that FDR control is generally still attained when we stratify the hypotheses^62^, because FDR controls the *proportion* (instead of the number) of false positives. For details, please refer to^62^. As such, the results can be considered to have accounted for multiple testing, in the sense that the false discovery rate (FDR) is controlled despite the presence of multiple phenotypes.

To identify independent significant risk loci, we employed PLINK-1.9 to perform LD-clumping with r^2^ = 0.01 and distance = 1000 kb, using 1000 Genomes East Asian sample as reference. SNP-to-Gene mapping was done using Bioconductor package ‘biomaRt’(version 2.48.2) on R-4.0.3.

The histograms and summary table of all phenotypes are shown in Supplementary Fig. 2 and Supplementary Data 10. We note that some of the phenotypes were normally distributed though some were not. Nevertheless, in large sample sizes with few covariates, violation of the normality assumption usually does not affect the validity of results^63^. There is no clear consensus on whether transformations (such as the rank-based inverse normal transformation, RINT) should be performed on (non-normal) phenotypes in GWAS. For example, Beasley et al.^64^ reported that RINT does not necessarily control type I error and may lead to reduced statistical power, while another study^65^ showed improved performance of the RINT approach. Intuitively, the untransformed approach keeps the original value of the phenotype and does not lead to loss of information, and is more interpretable. Here we performed analysis on both RINT-transformed^65^ and non-transformed phenotypes for all traits under study. As described below, on inspection of the QQ-plots, most traits have very similar distributions of p-values, except for four phenotypes. We primarily present our results of the non-transformed phenotypes except for the latter four which were RINT-transformed.

### Gene-based analysis with MAGMA

Gene-based analysis has been considered more powerful than SNP-based analysis performed in GWAS^66^. We utilized MAGMA (Multi-marker Analysis of GenoMic Annotation) v1.06 to conduct gene-based association tests with GWAS summary statistics of our phenotypes^13^. Briefly, MAGMA considers the aggregate effects of all variants in each gene to produce a gene-based test statistic. We employed the FDR procedure^67^ to control for multiple testing. In our gene-based study and the following analyses, results with FDR < = 0.05 are regarded as significant, while those with 0.05 < = FDR < = 0.2 are considered suggestive associations.

### Pathway analysis with GAUSS

We subsequently performed pathway enrichment tests with a powerful subset-based gene-set analysis method called GAUSS (Gene-set analysis Association Using Spare Signal)^68^, based on gene-based association results obtained by MAGMA. We utilized two collections of gene-sets derived from the Molecular Signature Database (MsigDB v6.2)^69^. The first is a collection of curated pathways (C2) which include canonical pathways such as KEGG, BioCarta, REACTOME, as well as chemical and genetic perturbations; the other is gene-ontology (GO) gene-sets (C5), which include biological processes, molecular processes, and cellular processes. Please refer to https://www.gsea-msigdb.org/gsea/msigdb/collections.jsp for details. If a significant association with a pathway is found, GAUSS also identifies the core subset (CS) of genes within the pathway that is driving the association.

### Transcriptome-wide association studies with S-Predixcan & S-Multixcan

We also employed other approaches to compute gene-based association results. MAGMA is a widely used approach, but it does not consider the functional impact of SNPs (e.g., impact on expression). S-PrediXcan is another gene-based analysis approach which *imputes* gene expression changes in relevant tissues due to genetic variations, using reference eQTL datasets such as the GTEx. This approach is also known as transcriptome-wide association study (TWAS)^70^. Here we considered 13 brain regions, including the amygdala, anterior cingulate cortex (BA24), caudate basal ganglia, cerebellar hemisphere, cerebellum, cortex, frontal cortex (BA9), hippocampus, hypothalamus, nucleus accumbens (basal ganglia), putamen (basal ganglia), spinal cord (cervical c-1) and substantia nigra. For S-PrediXcan, FDR correction was performed separately for each trait across all brain regions.

To increase statistical power to identify candidate genes, we also integrated the joint effects of expression changes across multiple tissues in a secondary analysis by ‘S-MultiXcan’^71^. S-MultiXcan combines evidence across tissues using multiple regression (fitting predicted expression as independent variables), which also takes into account the correlation structure.

### Polygenic risk score analysis

To evaluate genetic overlap of the studied phenotypes with other neuropsychiatric traits, we performed a PRS analysis. PRS aggregates the joint effect of multiple genetic variants, weighted by the effect size from GWAS summary statistics data. PRS were generated by PLINK 1.9 across 11 P-value thresholds (pthres) = {1e-06, 1e-05, 1e-04, 0.001, 0.01, 0.1, 0.2, 0.3, 0.4, 0.5,0.05} (multiple testing corrected by FDR, stratified by each exposure-outcome pair)^72^, LD-clumped at r^2^ = 0.1 within a distance of 1000 kb.

We constructed PRS for various neuropsychiatric disorders/traits, including educational attainment (EA; *N* = 1,131,881)^45^, cognitive performance (CP; *N* = 257,841; derived from scores of verbal-numerical reasoning from the UK Biobank and neuropsychological test results from the COGENT Consortium, details described in^45^), autism spectrum disorders (ASD; *N* = 46,350)^49^, attention deficit hyperactivity disorder (ADHD; *N* = 225,534)^73^, schizophrenia (SCZ; *N* = 320,404)^74^, bipolar disorder (BP; *N* = 413,466)^75^, and major depressive disorder (MDD; *N* = 194,548)^76^.

GWAS summary statistics were downloaded from the Social Science Genetic Association Consortium (SSGAC) (https://www.thessgac.org/), Psychiatric Genomics Consortium (PGC) (https://www.med.unc.edu/pgc) and The Integrative Psychiatric Research project (iPSYCH) (https://ipsych.au.dk/downloads/).

We employed linear mixed models in GEMMA to test for associations between PRS and phenotypes. The model was adjusted for age and sex as fixed effects. GRM was fit as a random effect, accounting for both relatedness and population stratification^77^.

In addition to the clumping and p-value thresholding (C + T) approach, we also employed SBayesR^78^ for PRS analysis. Briefly, this approach assumes a mixture model of the coefficients and performs Bayesian posterior inference to estimate the effect sizes of SNPs. The approach does not require selection of particular p-value thresholds. We followed the default settings of SBayesR and assumed a four-component mixture model for the coefficients (for details please refer to the original paper^78^).

We also tested for genetic overlap of our findings with other GWAS on dyslexia or reading abilities, as detailed below.

### Genetic overlap with findings from two related GWAS (Doust et al.^8^ and Wang et al.^22^), based on the top SNPs/genes reported

We performed SNP-set and gene-set analysis based on the top SNPs/genes reported from two relevant external studies (one on dyslexia by Doust et al.^8^ and the other on language/reading abilities in Chinese by Wang et al.^22^), to examine genetic overlap between the external GWAS and our HK study. Note that full GWAS summary statistics are not available from these two studies, so we focused on the top SNPs and genes reported.

Briefly, for SNP-set analysis, we first identified top SNPs (defined by p-values smaller than predefined cutoffs) from two independent GWAS datasets on dyslexia and reading abilities^8,22^. Then we extracted the same SNP-set from our data, and performed the Simes test^79^ and the aggregated Cauchy association test (ACAT)^80^ to examine whether the SNP-set as a whole was significantly associated with our studied traits. In other words, we tested for overlap in genetic signals across the external and HK datasets. The Simes and ACAT tests are established statistical methods for testing variant-set or gene-sets/pathways^80–82^, and are valid under dependent hypothesis tests.

Using the same analytic approach, we also performed gene-set analysis to examine genetic overlap across the external and local datasets. Similar to before, we first extracted top genes from the external datasets with (gene-based) *p*-values smaller than a predefined cutoff, then extracted the same set of genes from our sample, and tested whether the gene-set (as a whole) was significantly associated with the studied phenotypes. This replication analysis was conducted under various *p*-value cutoffs (*p* = 0.05, 1e-2, 1e-3, 1e-4, 1e-5 and 1e-6).

Besides, we also performed PRS analysis based on the above two external GWAS. The analytic strategies follow those described above, except that SBayesR was not used for PRS analysis due to the limited number of SNPs available. For Doust et al.^8^, summary statistics of the top 10,000 SNPs (corresponding to a p-value threshold of ~1.31e-6 after LD-clumping) were publicly available; for the other GWAS by Wang et al.^22^, summary data from the top SNPs (*p* < 1e-5) were available. Our replication analyses were therefore restricted to the SNPs with available summary statistics.

### Genetic overlap/dependence with the GenLang study, using full GWAS summary statistics

As for another study conducted by the GenLang Consortium (Eising et al.^9^), full summary statistics are available, hence enabling analysis to examine genetic overlap across different traits using whole-genome data. LD score regression (LDSC) is the standard approach for genetic correlation analysis, yet it has been reported that the method cannot reliably estimate genetic correlation for small or modest sample sizes^83^. It has been observed that for an (effective) sample size < 5000, unreliable and negative heritability may be reported. Here we tried LDSC on our sample, however, the heritability estimate for each reading/language trait was negative, corroborating with previous reports. We note that such negative estimates are inconsistent with previous twins/family studies that reported a significant heritable component^5,84^ of reading/language abilities. In addition, if a trait has negative heritability, genetic correlation with any other trait cannot be reliably estimated. We therefore turned to alternative approaches.

Inspired by a recent study^85^, here we employed the Hoeffding’s test^86^ to evaluate genetic dependence across phenotypes. We used the term ‘genetic dependence’ here to distinguish it from the standard ‘genetic correlation’ measure by LDSC. Following the above study^85^, Hoeffding’s test of independence is one of the methods that may serve as an alternative to LDSC under modest sample sizes, with satisfactory control of type I errors. Hoeffding’s test is a well-established non-parametric test based on examining the marginal and joint distributions of the two input variables (say *X* and *Y*)^86,87^. It is a non-parametric test based on the ranks of *X* and *Y* only. No assumptions are made on the distributions of *X* and *Y*, other than that they are continuous variables.

We followed a similar testing procedure as described in the previous study^85^. For each reading/language trait studied in the HK sample, we first performed LD-clumping based on GWAS results from our HK sample, and then extracted the same set of SNPs from Eising et al.^9^. Clumping was performed by plink (v1.9) by setting the physical distance threshold as 10,000 kb, and *r*^2^ threshold as 0.2. Five traits (word reading, non-word reading, spelling, phenome awareness, non-word repetition) were included from Eising et al.^9^.

We then performed the Hoeffding’s test (using the R package ‘independence’^86^ and *p*-values as input) for the phenotypes studied in our HK sample against the above 5 traits. We also performed PRS analysis following the approach described above.

### Correlation analysis of the effect sizes of top SNPs in HK and external samples

As a further exploratory analysis, we also evaluated the effect size correlations of the top associated SNPs (with *p* < 1e-5) from HK and GenLang samples^9^. Both Pearson and Spearman correlations were tested. We note that such correlations should be considered preliminary or crude measures of the true correlation of genetic signals, and more rigorous methods such as LDSC should be used to assess genetic correlation in future studies with larger sample sizes.

Compared to standard approaches like LDSC, we note that there are several limitations of this approach. Firstly, unlike LDSC, LD between variants is not accounted for. Secondly, the observed effect sizes are usually not equal to the true effect sizes^88–90^, and this was not accounted for in this approach. Since existing studies mostly focus on LDSC or other similar (advanced) methods, the performance of simpler approaches such as directly computing correlations among significant SNPs remains to be studied. Taken together, we consider this as an exploratory/preliminary analysis (and as an alternative to LDSC since the latter cannot be performed).

### Ethics approval

This study has received ethics approval from The Joint Chinese University of Hong Kong – New Territories East Cluster Clinical Research Ethics Committee (The Joint CUHK-NTEC CREC) (reference no: 2017.479).

### Reporting summary

Further information on research design is available in the Nature Research Reporting Summary linked to this article.

### Supplementary information

Supplementary Data 1

Supplementary Data 2

Supplementary Data 3

Supplementary Data 4

Supplementary Data 5

Supplementary Data 6

Supplementary Data 7

Supplementary Data 8

Supplementary Data 9

Supplementary Data 10

Supplementary Data 11

Supplementary Data 12

Supplementary Data 13

Supplementary Data 14

Supplementary Data 15

Supplementary Data 16

Supplementary Data 17

Supplementary Data 18

Supplementary Data 19

Supplemental Material File #1

reporting summary

## Data Availability

GWAS summary statistics of other neuropsychiatric disorders/traits were downloaded from the Social Science Genetic Association Consortium (SSGAC) (https://www.thessgac.org/), Psychiatric Genomics Consortium (PGC) (https://www.med.unc.edu/pgc) and The Integrative Psychiatric Research project (iPSYCH) (https://ipsych.au.dk/downloads/). Data of the top 10,000 associated SNPs from the GWAS on dyslexia was downloaded from 10.7488/ds/3465. Data of GWAS on reading/language-related traits from Eising et al. were downloaded from https://www.ebi.ac.uk/gwas/publications/35998220. Summary statistics of the most significant SNPs, genes and pathways (across all phenotypes) of the current study are available in supplementary tables. For further summary data supporting the findings of this study, please kindly make a request to the corresponding author. Individual-level data are not available due to confidentiality concerns.

## Code availability

For this study, no custom code or new algorithms were developed. The references provide details on the specific tools or programs applied. Any access or licensing requirements associated with the references’ codes can be obtained by consulting the original sources.
