# Supplementary figures and images for: A genome-wide association study of Chinese and English language phenotypes in Hong Kong Chinese children
Source: NPJ Sci Learn. 2024 Mar 27;9:26. doi: 10.1038/s41539-024-00229-7 (PMC10973362; doi:10.1038/s41539-024-00229-7)

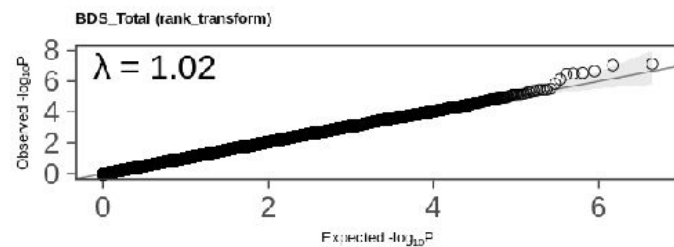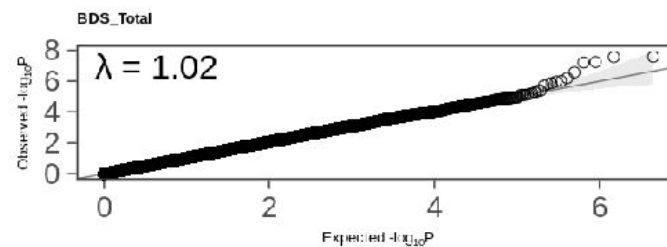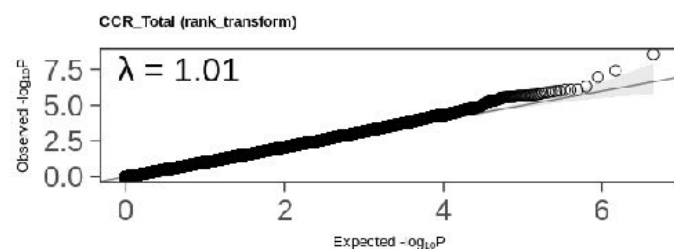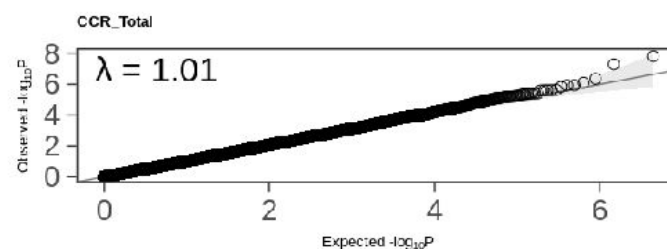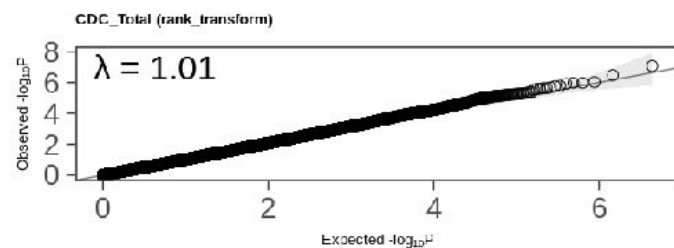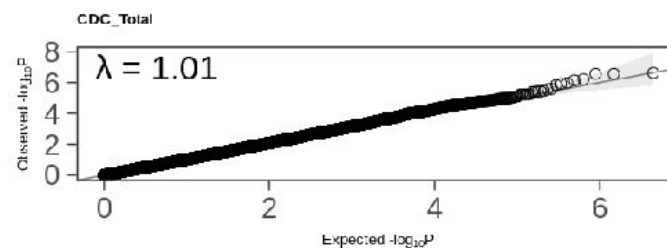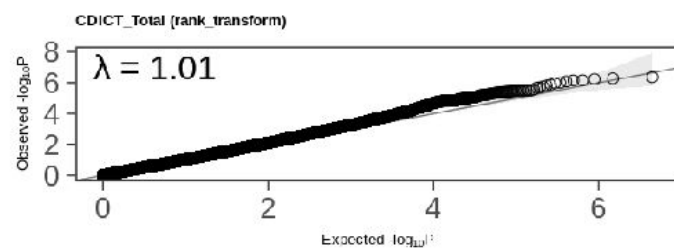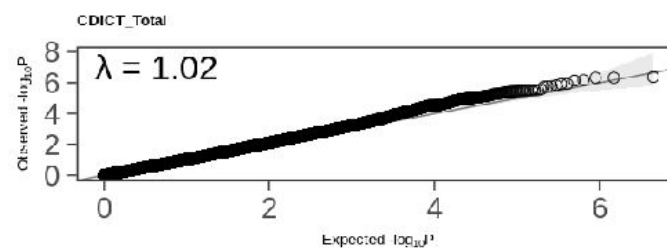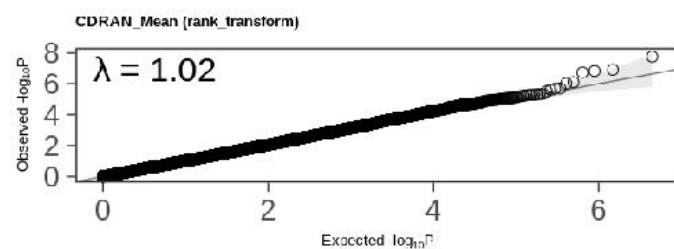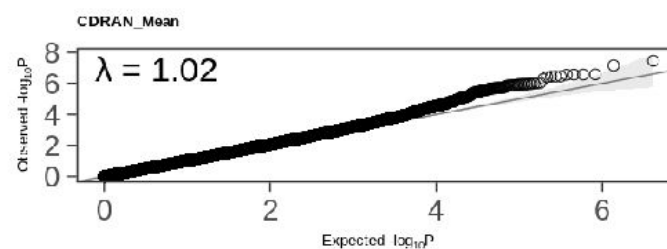

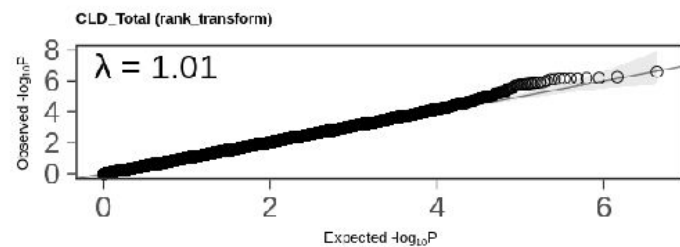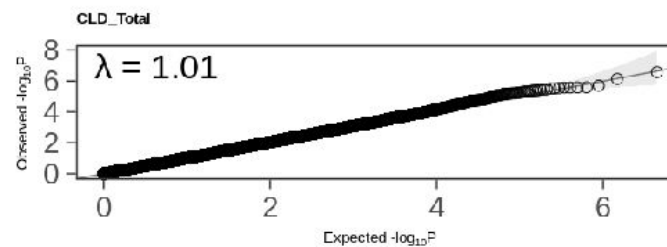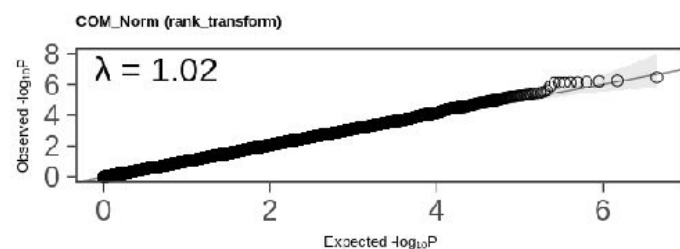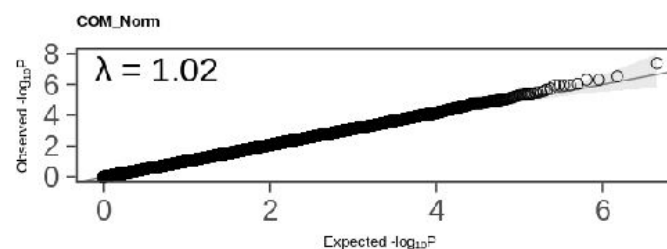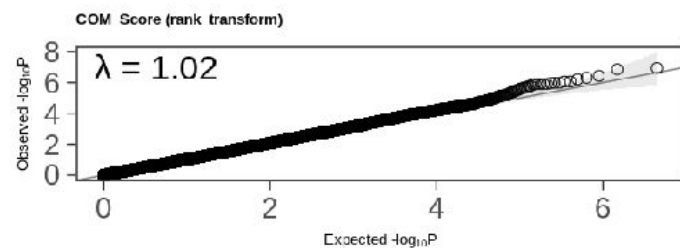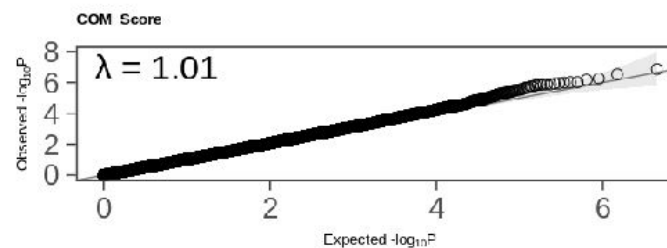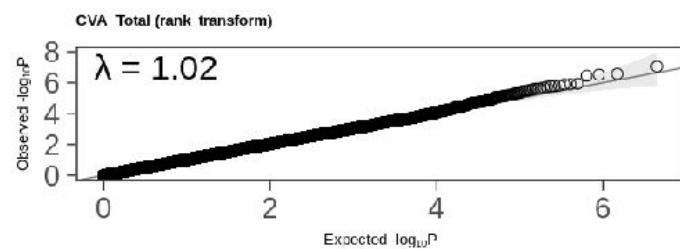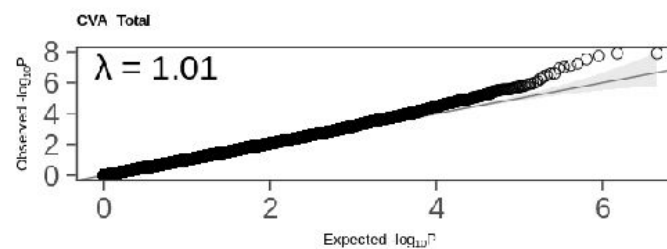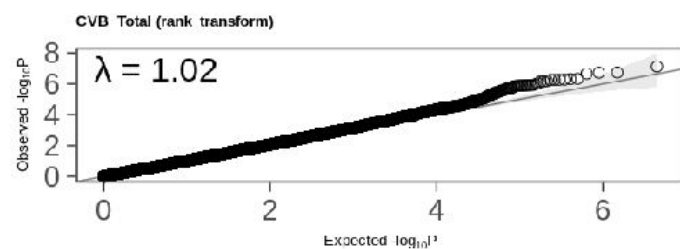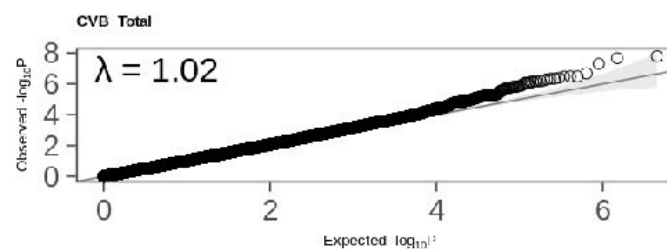

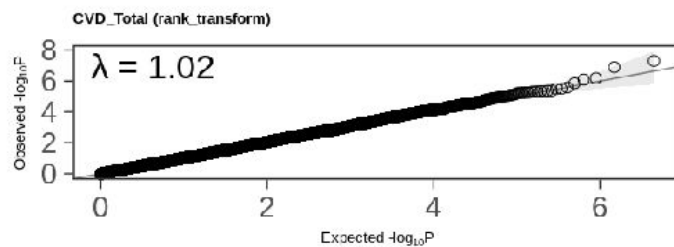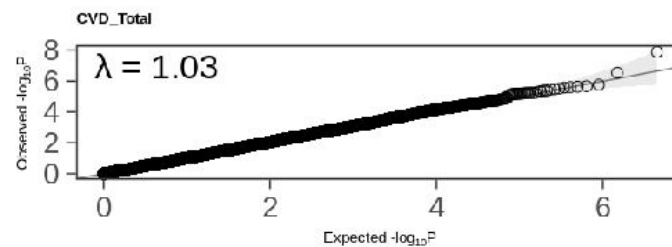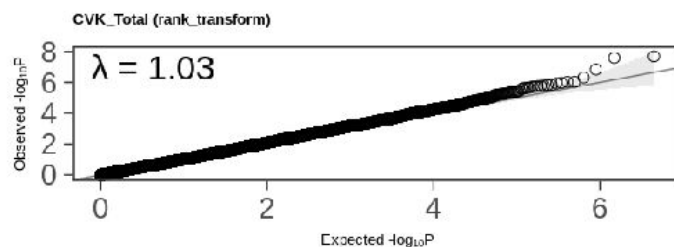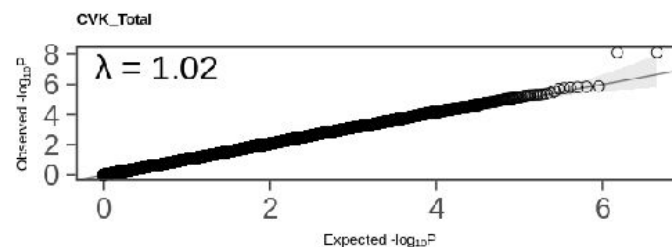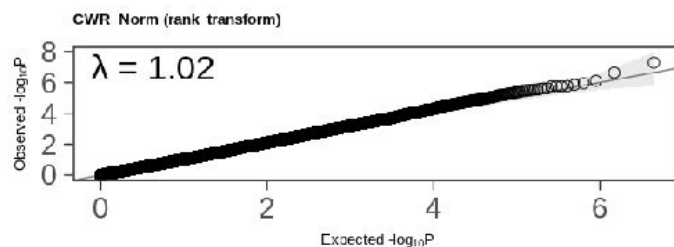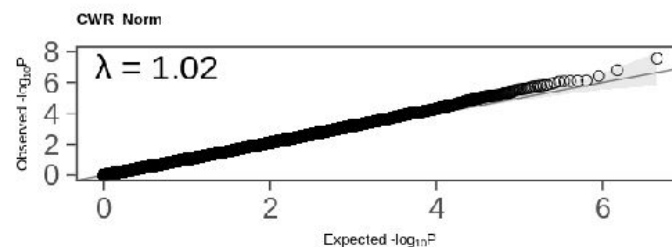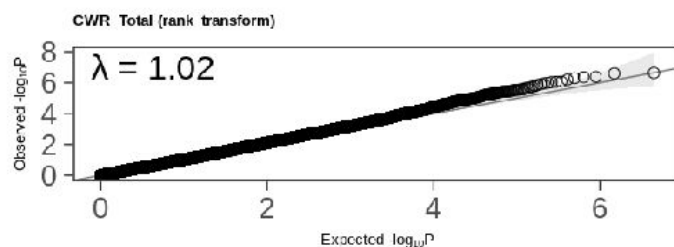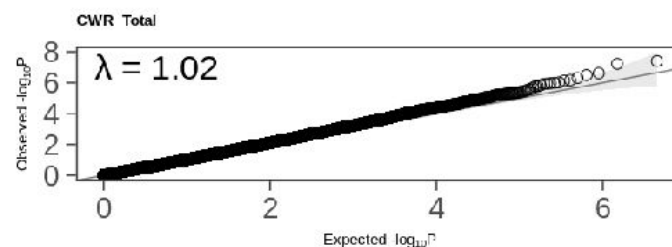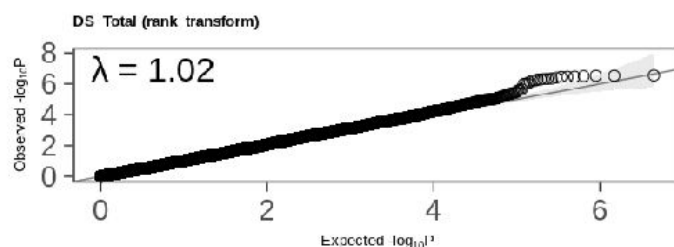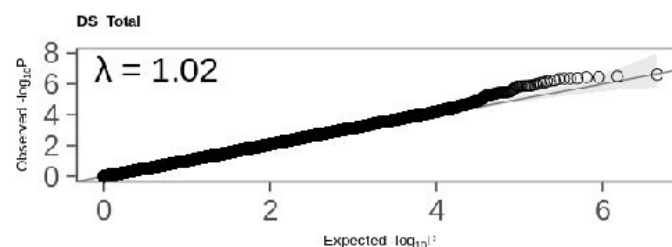

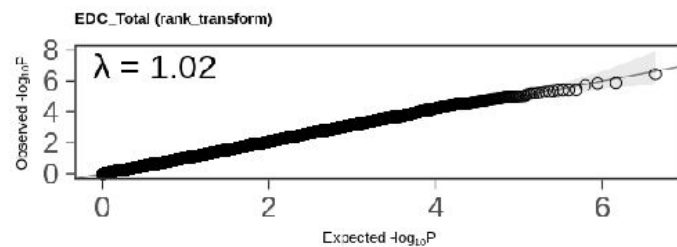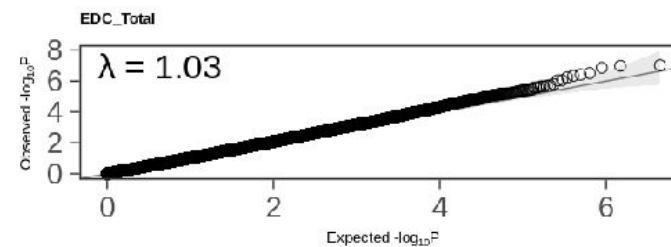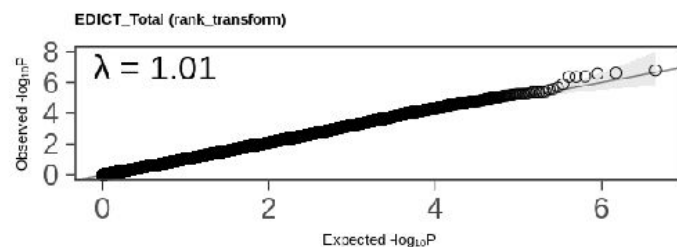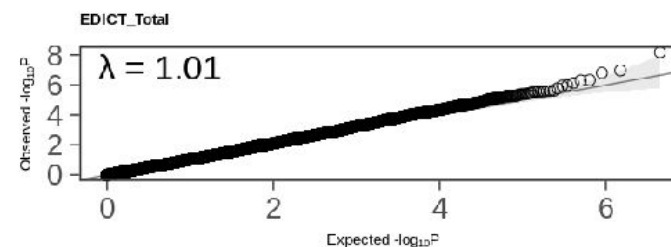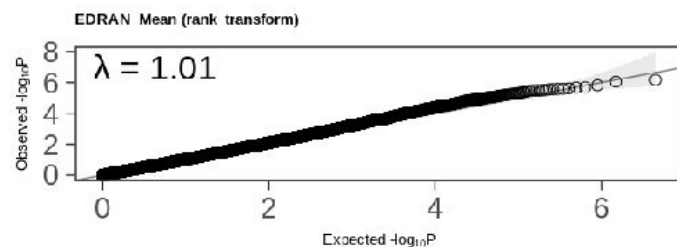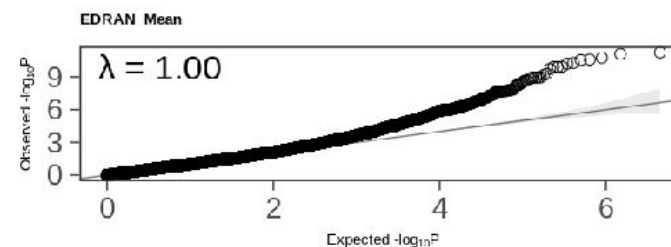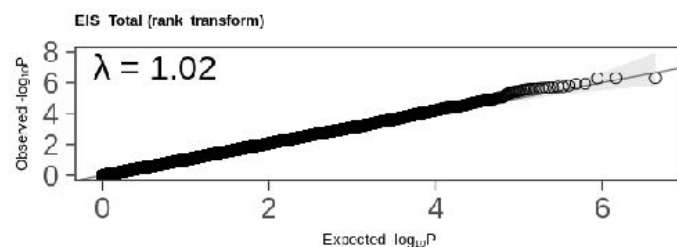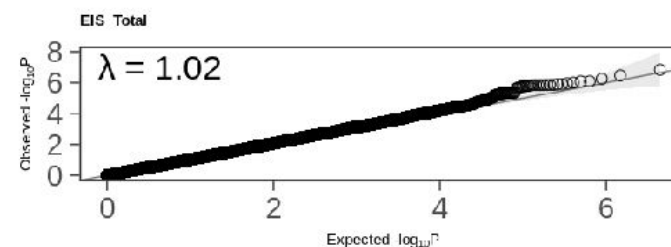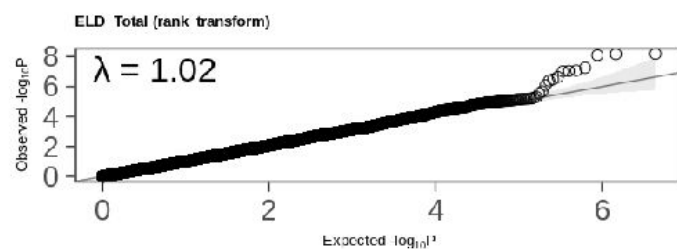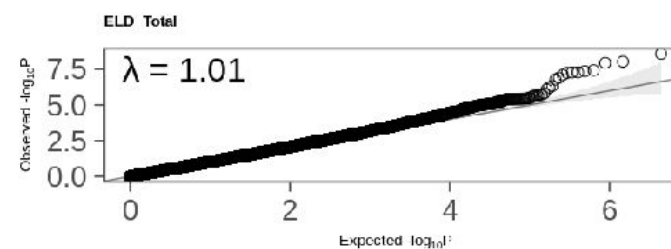

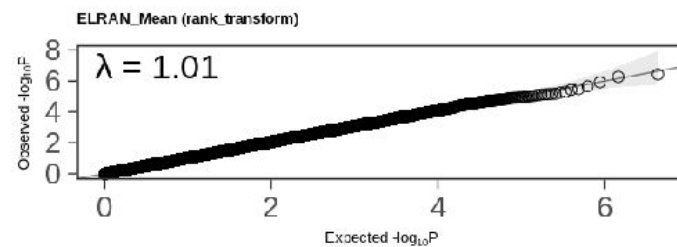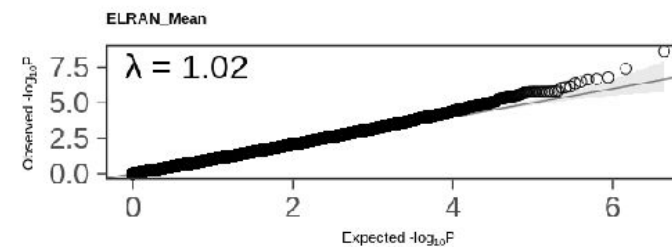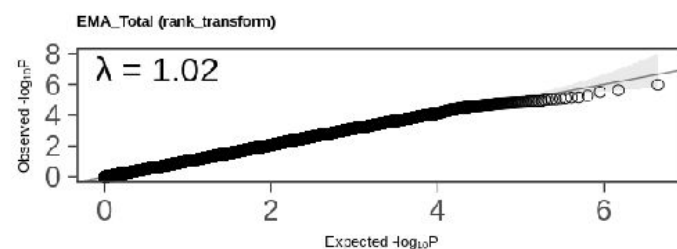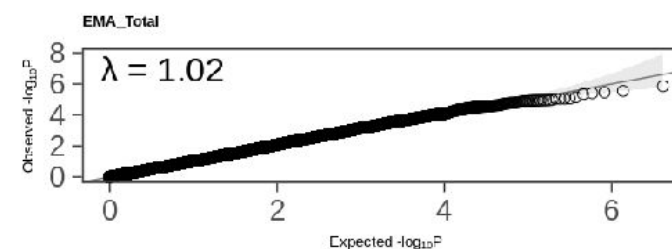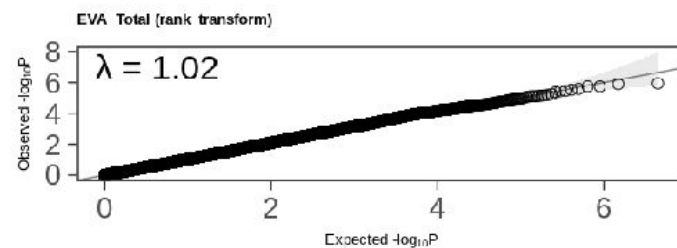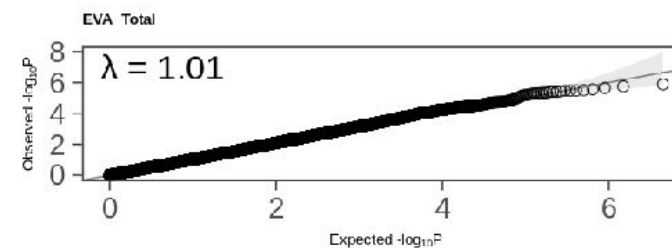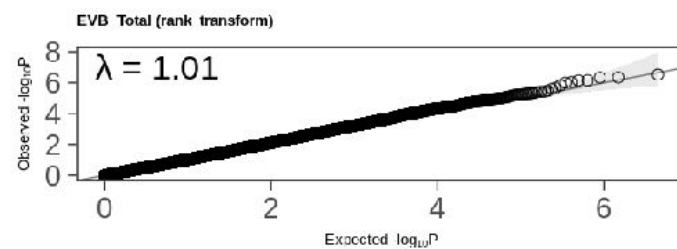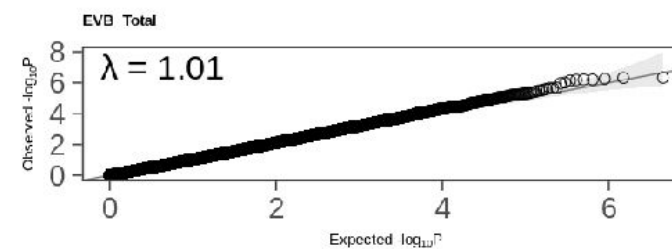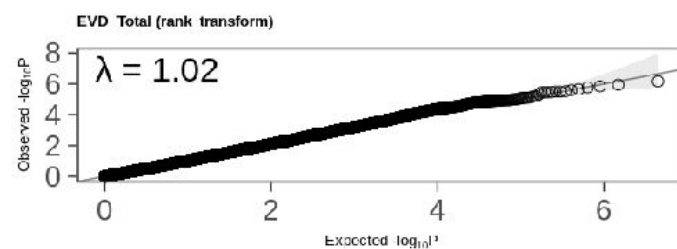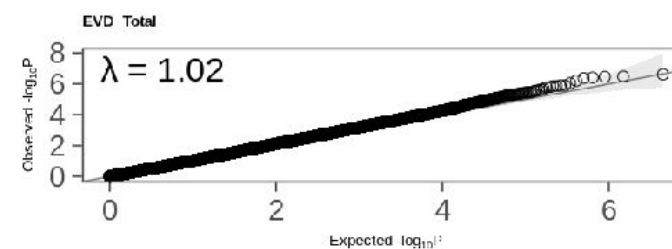

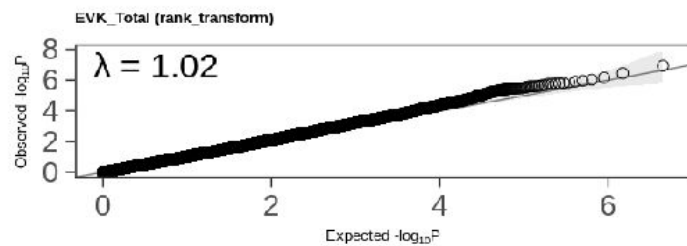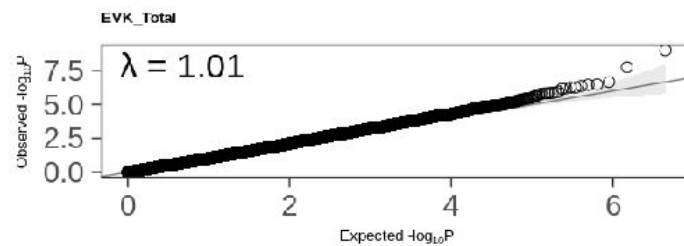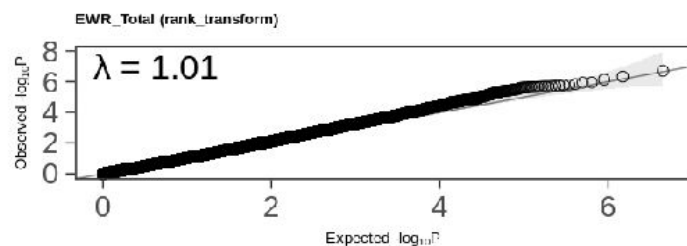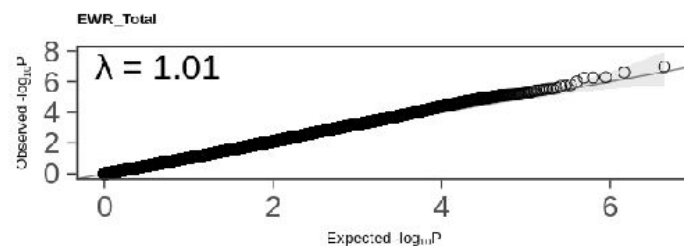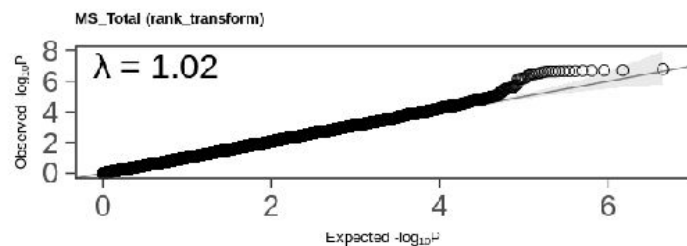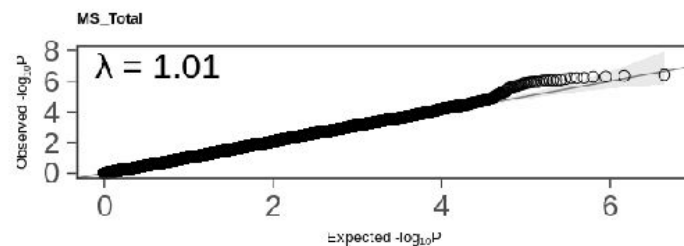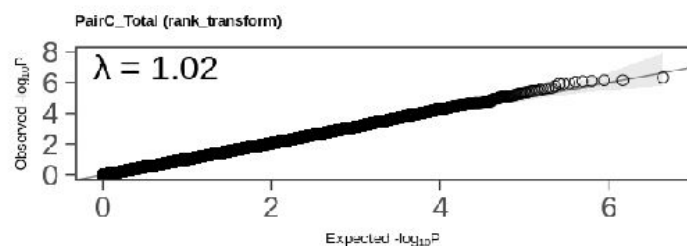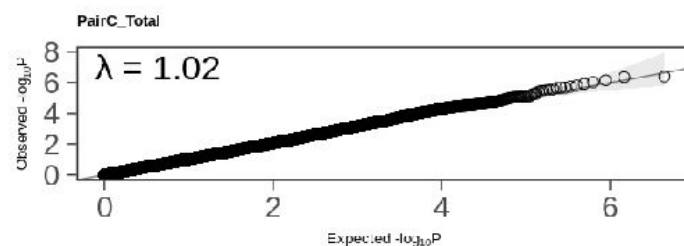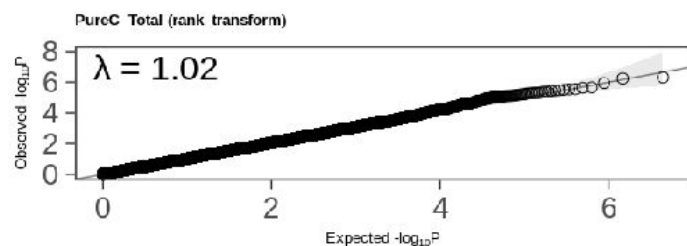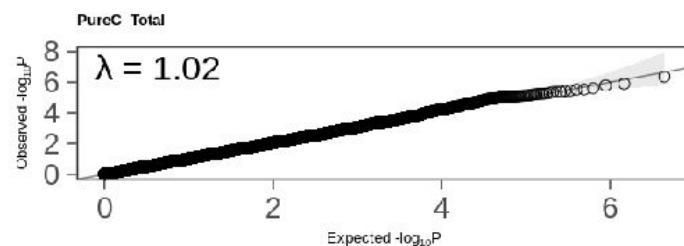

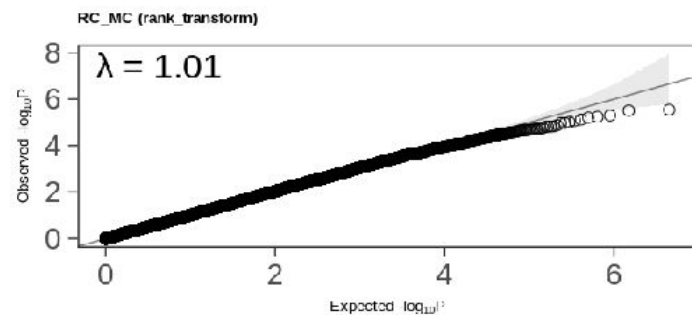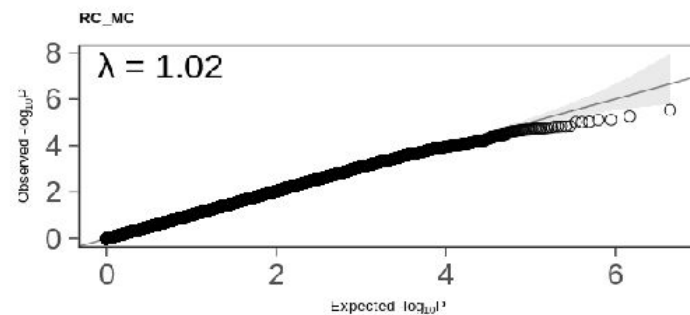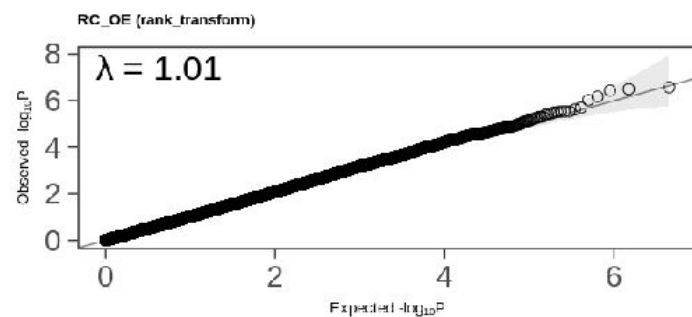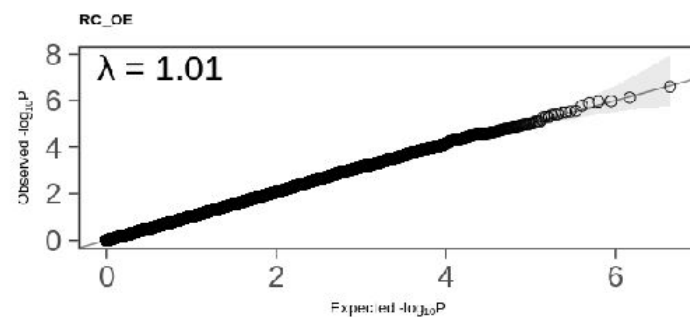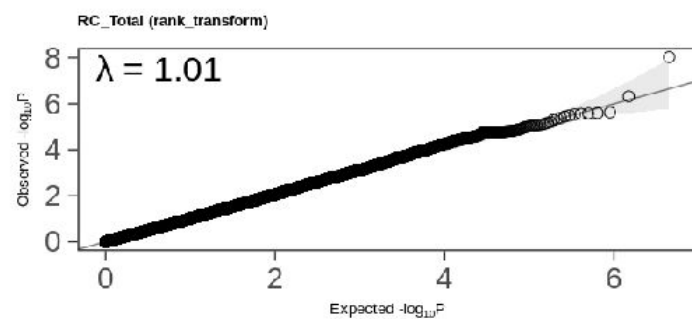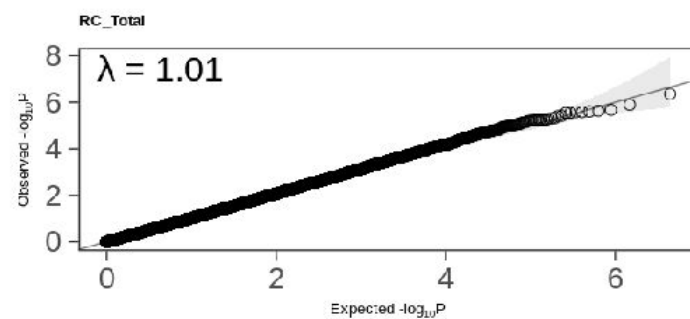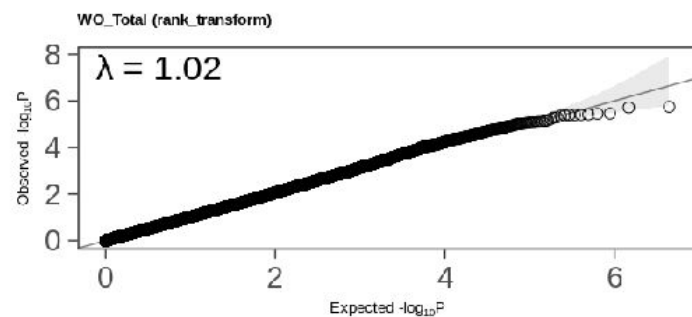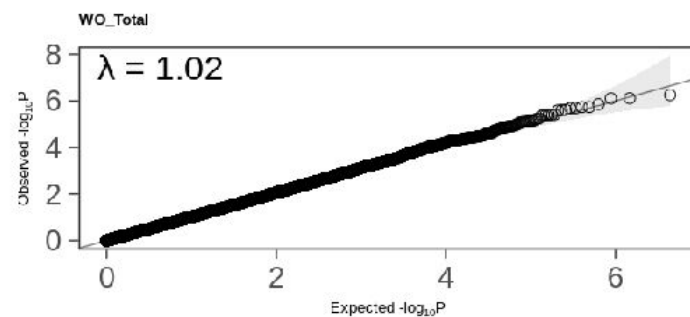

Supplement: Supplementary file 1 — Supplementary Data 1 [file 41539_2024_229_MOESM1_ESM.pdf]

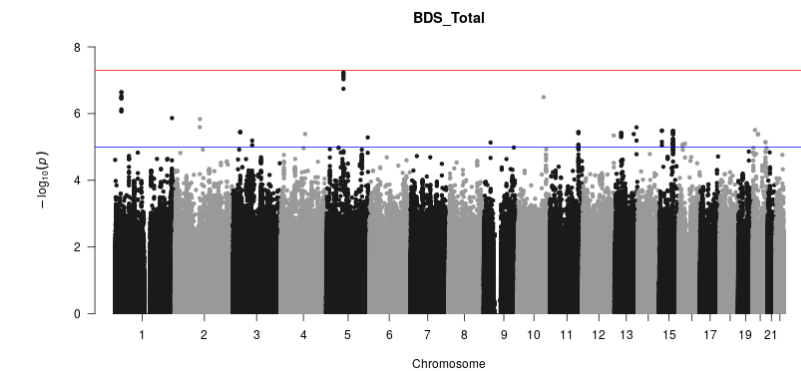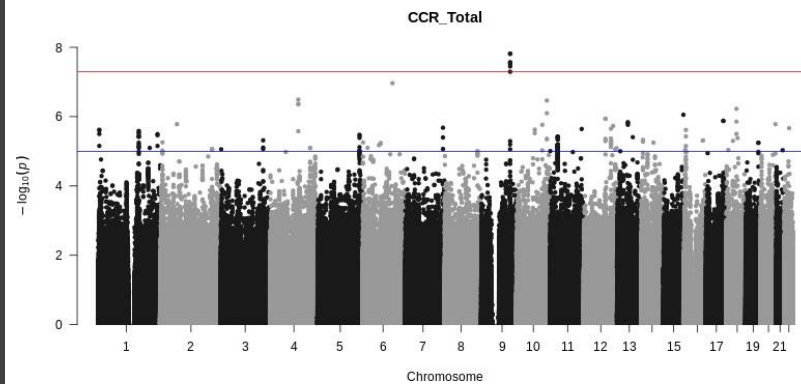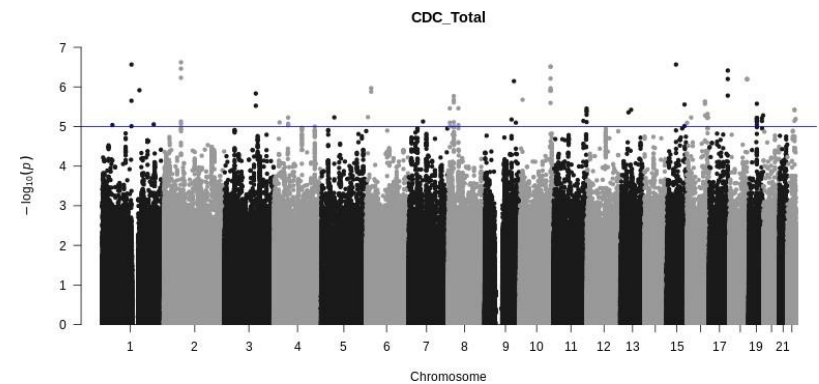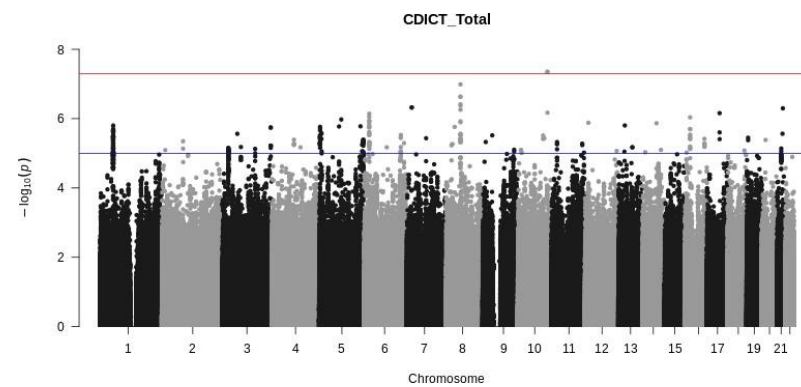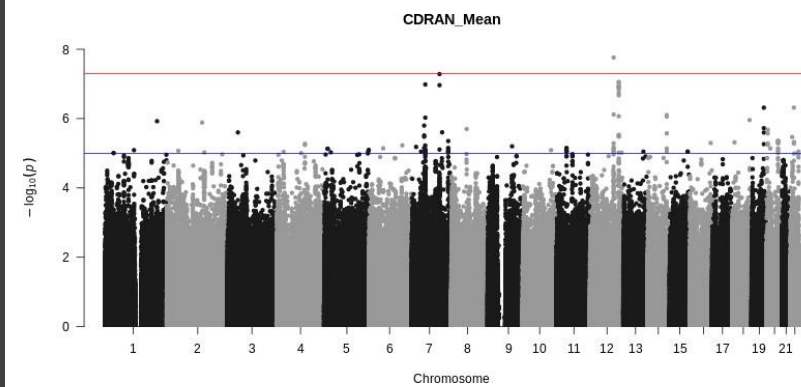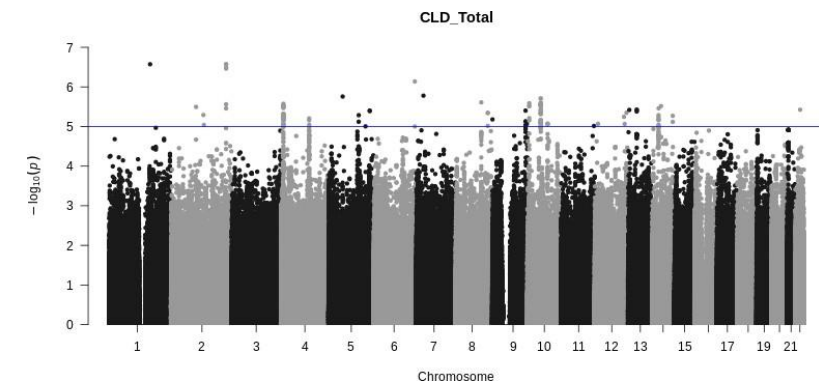

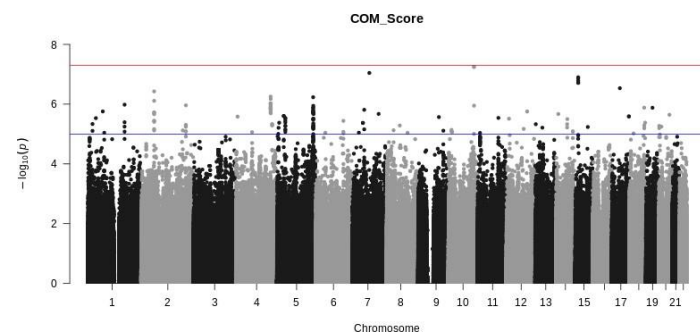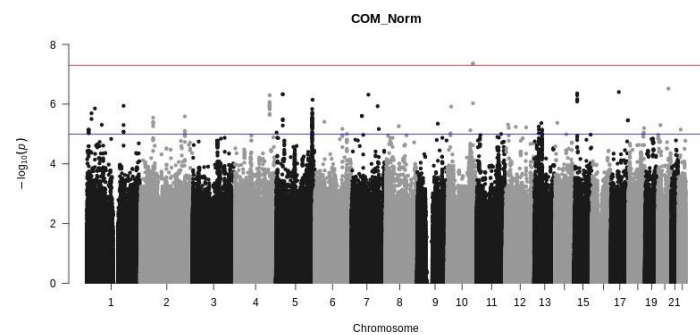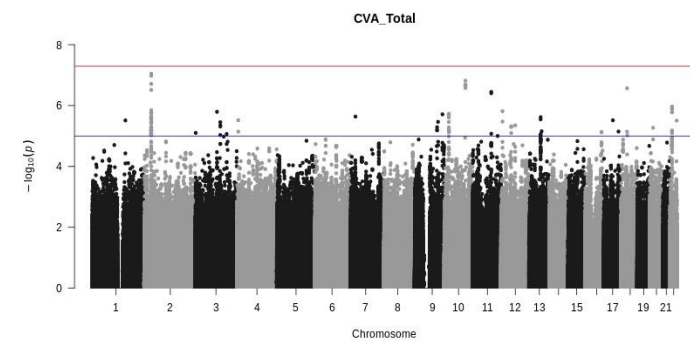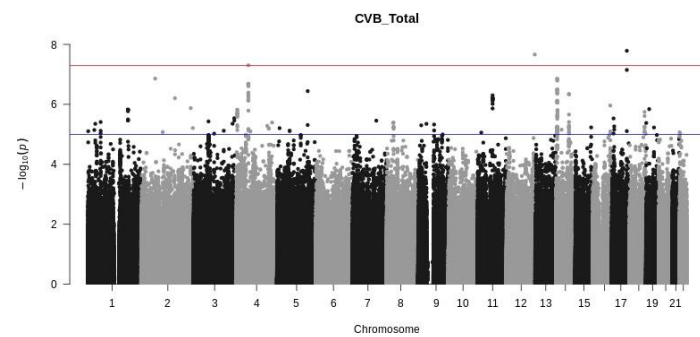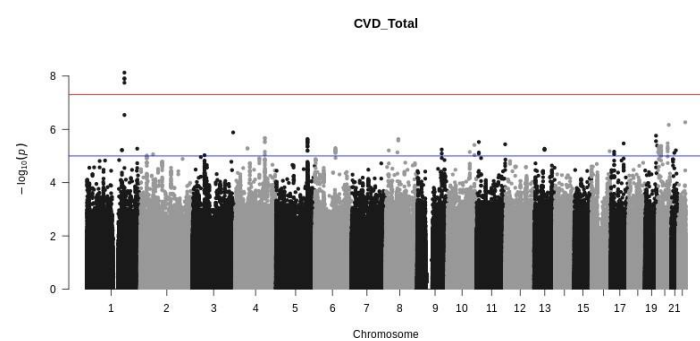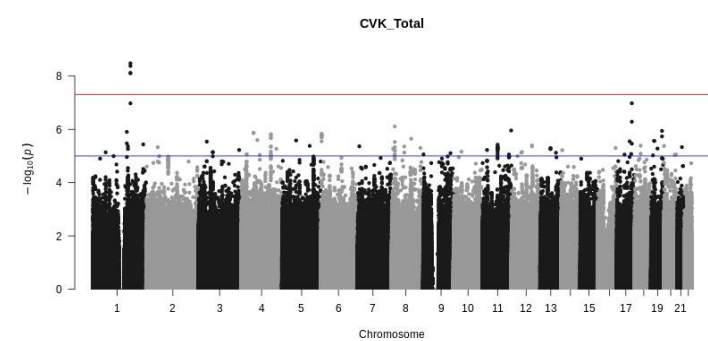

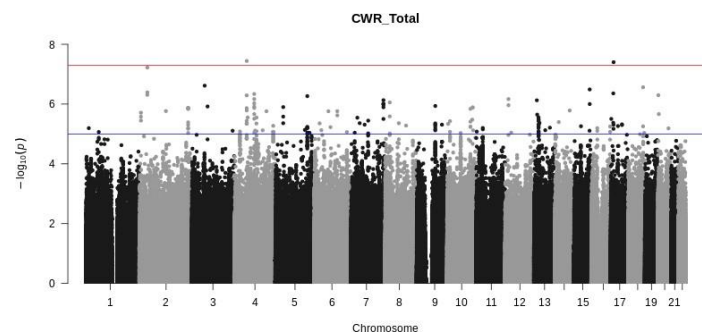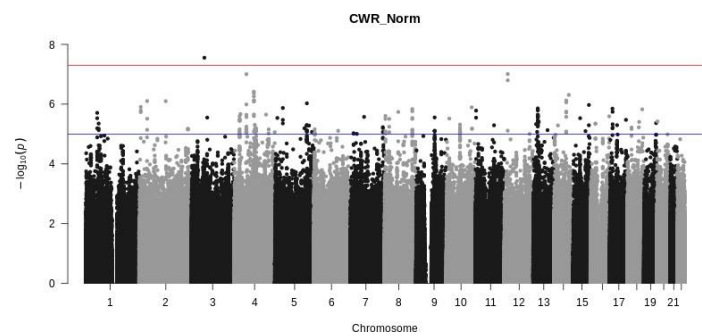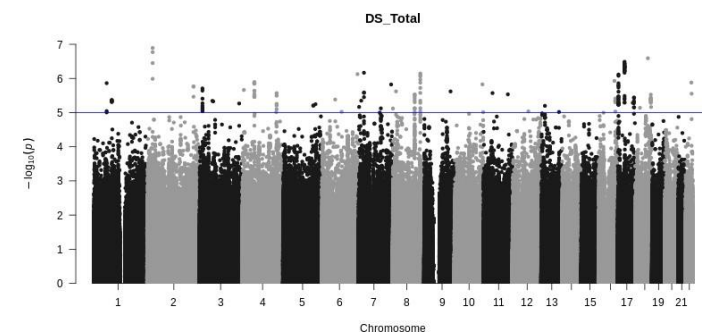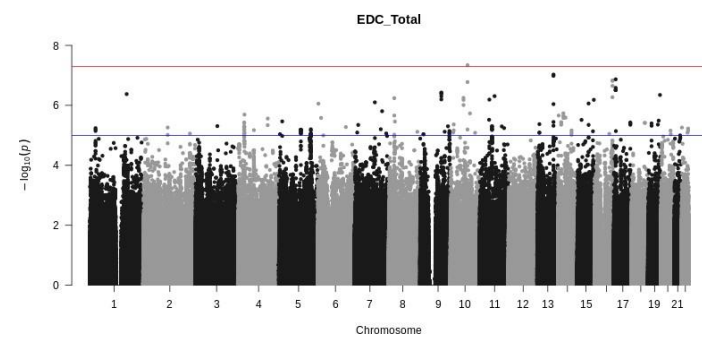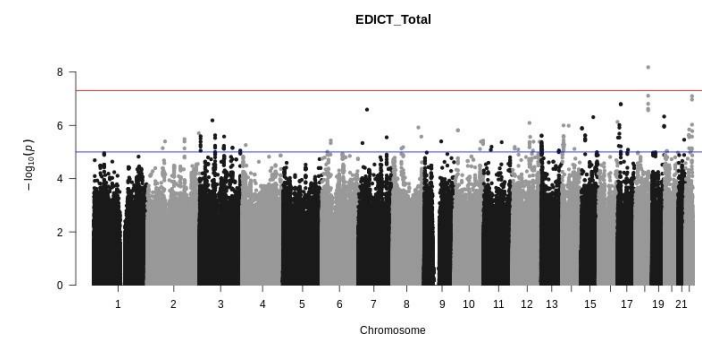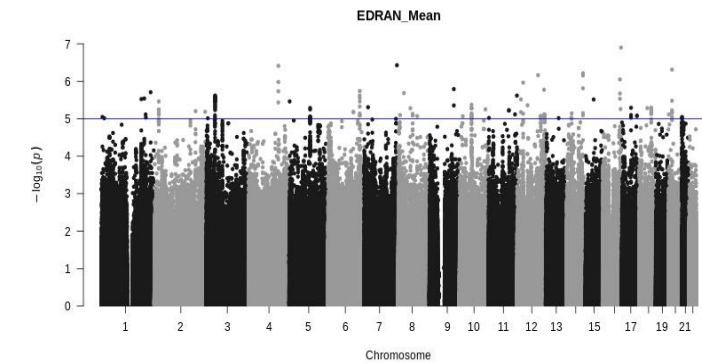

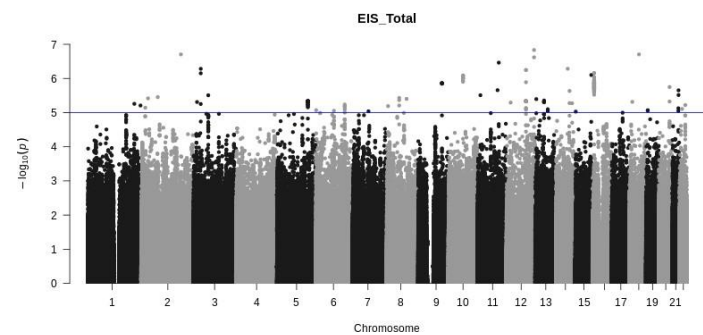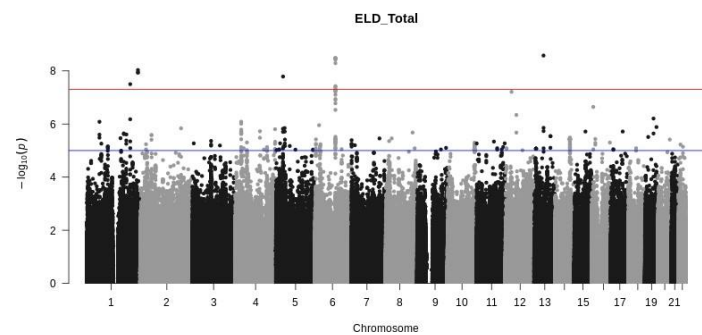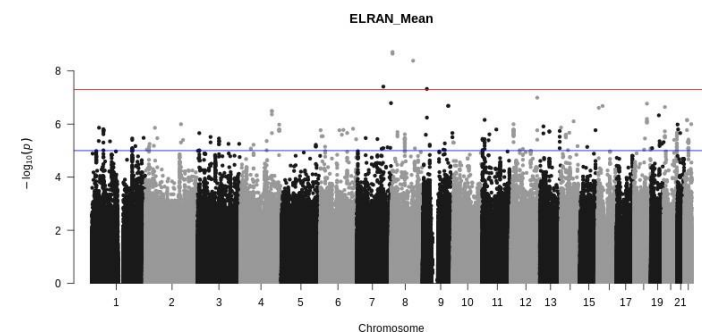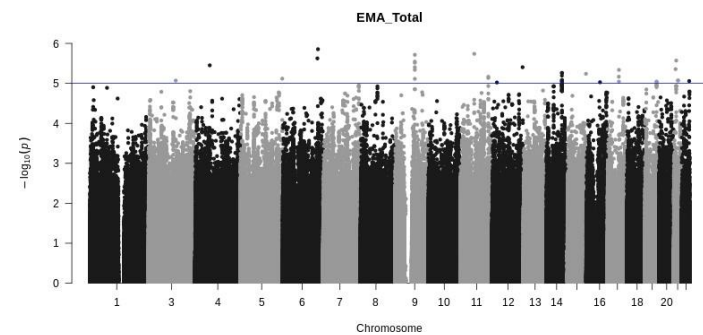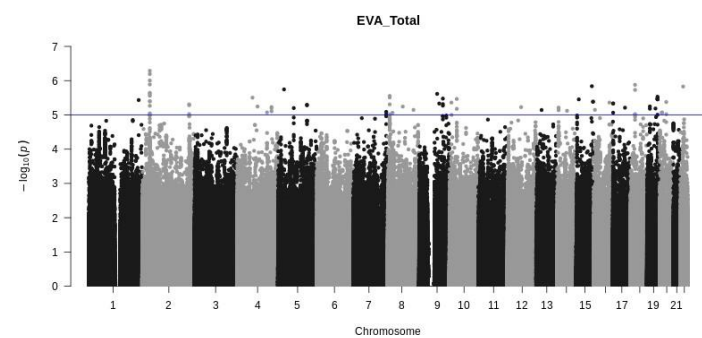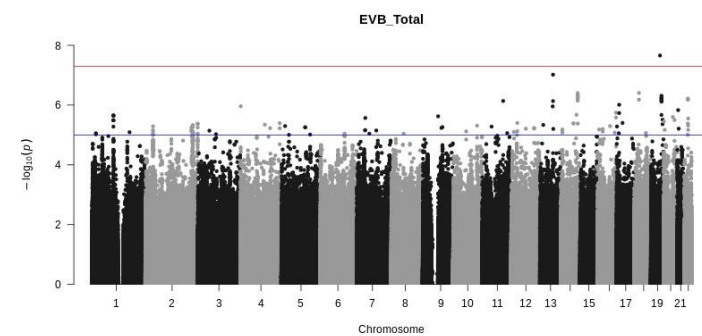

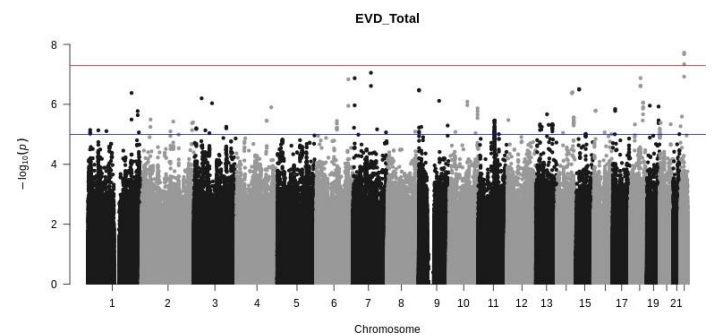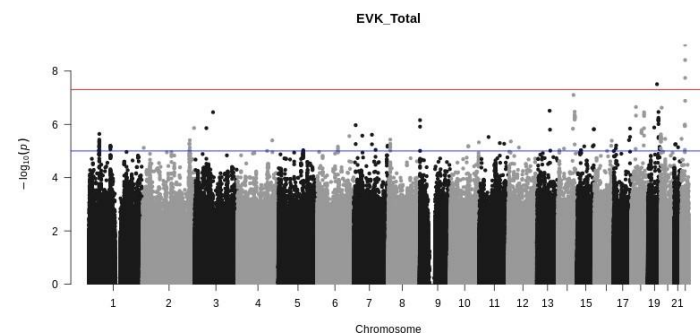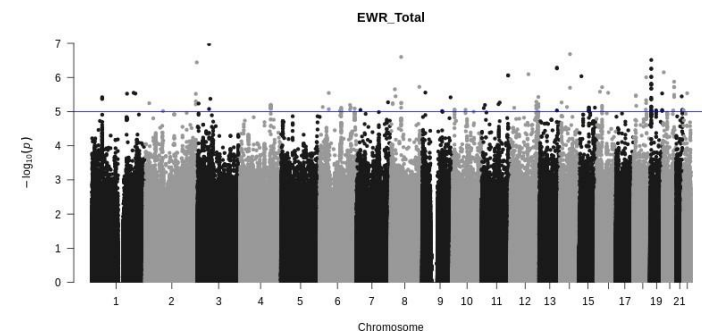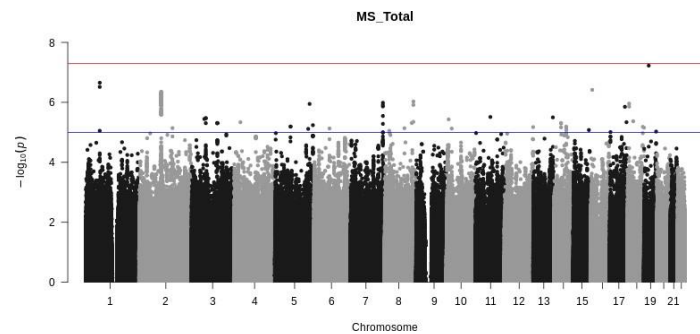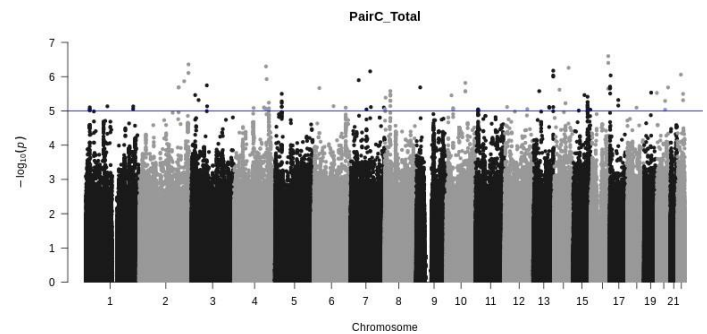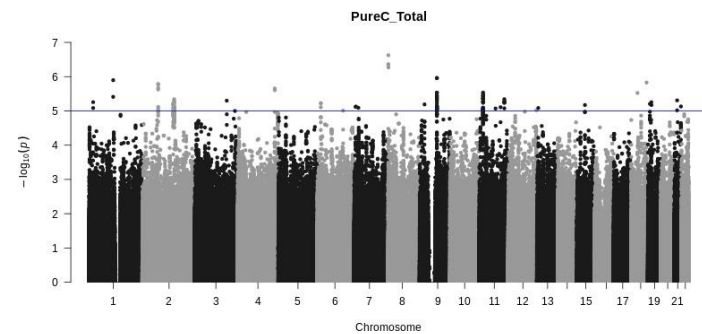

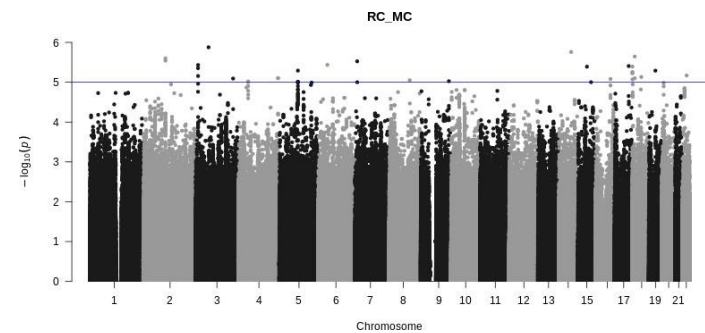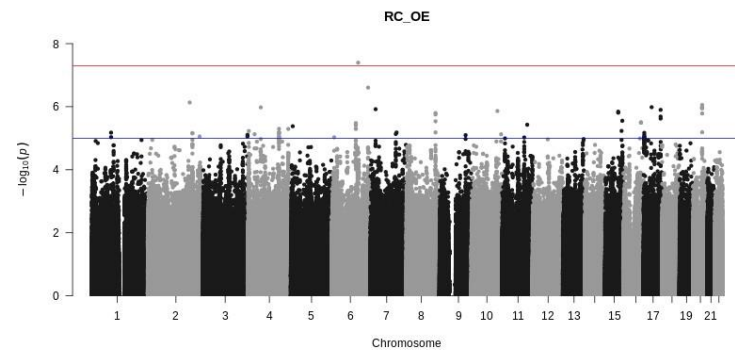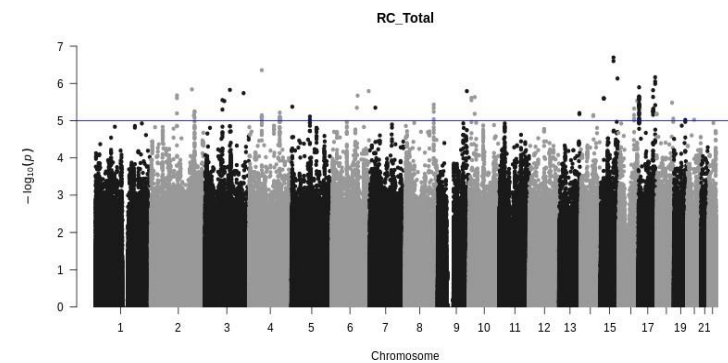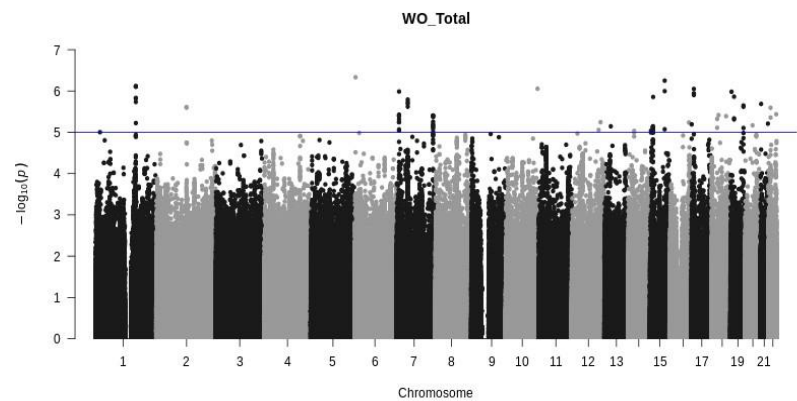

Supplement: Supplementary file 2 — Supplementary Data 2 [file 41539_2024_229_MOESM2_ESM.pdf]
