# Supplementary material for: A genome-wide association study of Chinese and English language phenotypes in Hong Kong Chinese children
Source: NPJ Sci Learn. 2024 Mar 27;9:26. doi: 10.1038/s41539-024-00229-7 (PMC10973362; doi:10.1038/s41539-024-00229-7)

Supplementary Information

A genome-wide association study of Chinese and English language phenotypes in Hong Kong Chinese children

Yu-Ping Lin, Yujia Shi, Ruoyu Zhang, Xiao Xue, Shitao Rao, Liangying Yin, Kelvin Fai Hong Lui, Dora Jue PAN, Urs Maurer, Kwong-Wai Choy, Silvia Paracchini, Catherine McBride, Hon-Cheong So

The supplementary information includes:

Supplementary Notes 2

Supplementary Data Captions 5

Supplementary Discussions 10

Supplementary References 11

Supplementary Figures 12

Supplementary Figure 1 12

Supplementary Figure 2 13

Supplementary Figure 3 14

Supplementary Figure 4 15

Supplementary Figure 5 16

Supplementary Figure 6 17

Supplementary Notes

Participants and testing procedures

The twins were drawn from a large longitudinal twin project in Hong Kong [1]. They came from different schools (government-run, government-aided, private and international schools) located in different districts of Hong Kong. All children were typically developing twins with Cantonese as their mother language and English as their second language.

Written consent was obtained from their parents before test administration. Children completed a systematic battery of literacy-related tasks in Chinese and English either in a laboratory setting, their school or their home by trained research assistants. All the tasks were finished in a given order that had been predetermined.

Measure descriptions

Working memory (BDS_Total)

Backward digit span was used to assess children's working memory. There were 8 levels of items in this task, with each comprising 2 trials of the same span length. For each trial, the experimenter presented a sequence of random one-digit numbers at the rate of one digit per second (e.g., 2, 5, 8; 3, 4, 8, 9). Participants were required to recall orally the sequence of digits in the reverse order. The task started with two-digit sequences, and the number of digits increased for each following subsequent level. One point was given to each correct response.

Chinese word reading (CCR_Total & CWR_Total/Norm)

In the character recognition task, children were asked to read aloud 80 Chinese single characters in order of graded difficulty. In the word reading task, children were asked to read aloud 150 two-character words arranged in order of ascending difficulty. One point was given for each correct recognition of single character or two-character word. CWR_Total refers to the total number of words read correctly (no time limit), while CWR_Norm refers to the adjusted score based on the Hong Kong Test of Specific Learning Difficulties in Reading and Writing [2].

Chinese delayed copying (CDC_Total)

This task required the children to copy an unfamiliar word after they saw it on a computer screen. The target words were low-frequency Chinese characters and consisted of 2–4 logographemes. Scores were given according to the copied logographemes. If a given logographeme was completely correct, 2 points were given. If a minor error was observed in a logographeme (e.g., missing stroke, extra stroke), 1 point was given.

Chinese dictation (CDICT_Total)

Children were asked to write 25 two-character Chinese words in the order of increasing character complexity and age of acquisition. A two-point scale was developed to code the children's writing. One point was given if the child could write down at least one radical of the character or s/he only made a mistake in one of the radicals (e.g., missing stroke, additional component) and two points were given if s/he could write the whole character correctly.

Chinese digit rapid naming (CDRAN_Mean)

The task consisted of eight rows of five digits [e.g., 2, 6, 5, 7, and 9]. These digits were arranged in different orders for each row. They were presented on a page and children were asked to name the digits in Cantonese as quickly and accurately as possible. Two trials were completed and the average time in seconds was recorded.

Chinese orthographic knowledge (CLD_Total)

This task contains 60 characters in which 30 of them were rear but real Chinese characters and 30 of them were non-characters. Children were required to recognize whether the character is a real character or not. A point was awarded for each correctly identified item.

Chinese 1 Min Word Reading (COM_Score/COM_Norm)

This task asks children to read 90 highly-frequent Chinese two-character words as quickly and accurately as possible within one minute. The total number of words read correctly within one minute was recorded. If a participant finished the task in less than one minute, the time duration to complete the task was also recorded. The raw score (COM_Score) was calculated as (number of words read correctly ∗ 60)/ (duration in seconds); this score was then converted to age-adjusted score (COM_Norm) based on The Hong Kong Test of Specific Learning Difficulties in Reading and Writing [2] norm.

Chinese vocabulary knowledge (CVK_Total= CVA_total + CVB_total + CVD_total)
This task contains 48 items presented in the order of increasing difficulty. The first 10 receptive vocabulary items were presented with four pictures along with an orally presented Chinese word. The children were asked to choose the correct picture that matched the Chinese word. In the subsequent 12 expressive vocabulary items, the children were asked to name a given picture in Chinese (e.g., compass). Each item was scored either 0 or 1 in these 22 items. The last 26 vocabulary definition items were orally presented with a Chinese word that represented a concept or an object. Children were asked to orally define the word. Children were given a score of either 0, 1, or 2 for each item based on how close their response was to the correct answer.

Chinese discourse skills (DS_Total)
This task was to measure children's skills in drawing inferences between sentences and integrating them to form a meaningful discourse. The children were first presented orally with three to five sentences and were asked to reorder them into a meaningful discourse. The content of the discourse ranged from a narration to procedures or factual information. There was one practice and 11 test items in the order of ascending difficulty. One to four point(s) were given for each correctly ordered three to six-sentence items respectively.

English delayed copying (EDC_Total)
This task requires children to copy an unfamiliar word after they saw it on a computer screen. The target words consisted of 6-11 letters. Children were asked to write the target word on an answer sheet. Scores were given according to copied letters and the letters' position separately. As each first and last letter was relatively important and easier to remember, bonus points were given if the first or last letter was correct.

English dictation (EDICT_Total)
This task required the participants to write out 18 English compound words. A two-point scale was developed to code each item. One point was given for each correctly spelt morpheme, or word within the compound word.

English digit rapid naming (EDRAN_Mean)
The task consisted of eight rows of five digits (e.g., 2, 5, 6, 7, and 3). These digits were arranged in different orders for each row. They were presented on a page and children were asked to name the digits in English as quickly and accurately as possible. Two trials were completed and the average time in seconds was recorded.

English invented spelling (EIS_Total)
The task consists of 12 pseudo-words containing three to six letters. All the 12 words are single-syllable words. The children heard a recording of a native English speaker saying the target word and they were instructed to spell out the English words that they heard on a sheet of paper. A 5-point scale (0–5 points) was used for each spelling.

English orthographic knowledge (ELD_Total)
This task required the participants to identify an English nonword from three similar stimuli. There was one practice item and 30 experimental items. For each item, three similar stimuli, including a real word, a pseudoword and a nonword were presented to the participants. The participants were asked to recognize one word which did not look like a real word. One point was given to the correct answer.

English letter rapid naming (ELRAN_Mean)
The children were presented a list of English letters and were asked to name the letters as quickly and accurately as possible. Two trials were completed and the average time in seconds was recorded.

English morphological awareness (EMA_Total)
This task includes 4 practice items and 20 test items presented in ascending difficulty. For the first 11 test items, children were required to create a new word based on a given compound word example. In the next 4 items, children were presented with a picture and a sentence and were asked to present the answer both orally and in written form. For example, *this boy knows how to RICK. What is he doing? He is ____. The correct answer was ricking*). One point was given to each correct response for these 15 items. For the remaining five test items, children were asked to make up an English word which best described the newly created object presented in a scenario without being given hints on the morphological structure. For example, *what do we call a house which is made of corn? The model answer was corn-house.* Answers were rated on a 0- to 4-point scale according to the rationale given in the study of [3].

English vocabulary knowledge (EVK_Total= EVA_Total + EVB_Total + EVD_Total)
This task included 45 items presented in order of ascending difficulty. For the first 15 receptive vocabulary items, the children were asked to choose the right picture among four alternatives that best represent an orally presented English word. In the next 15 expressive vocabulary items, the children were asked to name a picture in English (e.g., globe). One point was marked for each correct response in these 30 items. In the final 15 vocabulary definition items, children were asked to give the definition of an orally presented English word. Children were given a score of either 0, 1, or 2 for each item according to how close their response was to the correct answer.

English word reading (EWR_Total)
This task requires children to read 60 English words in the order of graded difficulty. One mark was given for each word pronounced correctly.

Morphosyntactic skill (of Chinese) (MS_Total)
This task measured children's skills in detecting and correcting morpho-syntactic errors in Chinese sentences. They were then asked to circle the error and replace it with a correct word. There was a total of 2 practice and 18 test items. In each item, 1 point was given for correct identification of the error and 2 points for accurate word replacement (one point for accurate syntactic use and one point for correct meaning in the sentence, 0.5 point for partially correct answer).

Pair cancellation (PairC_Total)
This task was to measure children's attention control. Children were presented with a piece of paper covered with randomly sequenced images of dogs, balls and cups. They were asked to circle as many ball-dog pairs with the dog after the ball as they could in 2 minutes.

Pure Copying of Unfamiliar Scripts (PureC_Total)
The participants were asked to copy 10 words from two unfamiliar scripts (five for Hebrew and five for Korean). Each Korean word consisted of three to four segments and partial scoring was used to score the shape and position for each segment (maximum 2 points for each correct answer in each category, 1 point was given for a similar shape or position. If the shape was scored as 0, a position point was not given). For Hebrew, each word consisted of three to six segments. They were scored by their position (1 point for each word), horizontal alignment (1 point for each word) and shape (1 point for each segment).

Reading comprehension (in Chinese) (RC_Total = RC_MC + RC_OE)
This task required children to read three passages in Chinese and answered the following multiple-choice and open-ended questions after each passage. One point was given for a correct answer to each of the 12 multiple-choice questions. For the open questions, one point was allocated to a correct answer for four of the questions, and two points were given for correct answers to four relatively difficult questions.

Chinese word order (WO_Total)
This task was used to measure the children's understanding of some basic sentence structure rules in written Chinese. It includes a total of 20 items. The children were asked to arrange three to eight sentence fragments

to form a syntactically correct sentence. Items were scored in Excel and the scoring algorithm created by [4].

Supplementary Data Captions

Supplementary Data 1:
Quantile-quantile plot for each rank-transformed and untransformed trait (except for BDS_Total, CVA_Total, CDRAN_Mean, and EDRAN_Mean; lambda refers to the genomic inflation factor).

Supplementary Data 2:
Manhattan plots for the 34 phenotypes under study.

Supplementary Data 3 Significant single-variant associations from GWAS (MAF > 0.05):

T1) All single nucleotide polymorphisms (SNPs) that passed genome-wide significance (p<5e-8) (all results with imputation quality score Rsq > 0.3 are shown)
Legend:
- A2: effect allele.
- MAF: minor allele frequency.
- BP (position of the snp): base pair.
- AvgCall: average call rate.
- Rsq: imputation quality score.

T2) All genetic variants with FDR<0.1 in SNP-based analysis

T3) Independent risk loci (LD-clumped with r2 threshold 0.01, including SNPs with MAF > 0.05)
Legend:
- S0001: Number of clumped SNPs (SNPs in LD) with p<1e-03.

Supplementary Data 4 Significant S-Predixcan results:

Top 100 S-Predixcan results after correction for multiple testing
Legend:
1. var_g: variance of the gene expression, calculated as W' * G * W (where W is the vector of SNP weights in a gene's model, W' is its transpose, and G is the covariance matrix).
2. pred_perf_r2: (cross_validated) R2 of tissue model's correlation to gene's measured transcriptome (prediction performance).
3. pred_pref_pval: pval of tissue model's correlation to gene's measured transcriptome(prediction performance).
4. n_snps_used: number of snps from GWAS that got used in S-Predixcan analysis.
5. n_snps_in_cov: number of snps in the covariance matrix.
6. n_snps_in_model: number of snps in the model.
7. FDR.adjust : Calculated by the R program p.adjust using Benjamini-Hochberg procedure (BH).

(please also refer to https://github.com/hakyimlab/MetaXcan)

Supplementary Data 5 Significant S-Multixcan results:

Significant S-Multixcan results after correction for multiple testing (FDR<0.2)
Legend:
1. n: number of tissues available for this gene.
2. n_indep: number of independent components of variation kept among the tissues' predictions. (Synthetic independent tissues).
3. p_i_best: best p-value of single-tissue S-PrediXcan association.
4. t_i_best: name of best single-tissue S-PrediXcan association.
5. p_i_worst: worst p-value of single-tissue S-PrediXcan association.
6. t_i_worst: name of worst single-tissue S-PrediXcan association.
7. eigen_max: In the SVD decomposition of predicted expression correlation: eigenvalue (variance explained) of the top independent component.

8. eigen_min: In the SVD decomposition of predicted expression correlation: eigenvalue (variance explained) of the last independent component.
9. eigen_min_kept: In the SVD decomposition of predicted expression correlation: eigenvalue (variance explained) of the smalles independent component that was kept.
10. z_min: minimum z-score among single-tissue S-Predican associations.
11. z_max: maximum z-score among single-tissue S-Predican associations.
12. z_mean: mean z-score among single-tissue S-Predican associations.
13. z_sd: standard deviation of the mean z-score among single-tissue S-Predican associations.
14. tmi: trace of T * T', where Tis correlation of predicted expression levels for different tissues multiplied by its SVD pseudo-inverse. It is an estimate for number of independent components of variation in predicted expression across tissues (typically close to n_indep).
15. FDR.adjust : Calculated by the R.program p.adjust using Benjamini-Hochberg procedure (BH).

(please also refer to https://github.com/hakyimlab/MetaXcan)

Supplementary Data 6 Significant gene-based associations results:

Significant gene-based results (MAGMA) after correction for multiple testing (FDR<0.05)

Legend:

1. START/STOP: the annotation boundaries of the gene on that chromosome (this includes any window around the gene applied during annotation).
2. NSNPS: the number of SNPs annotated to that gene that were found in the data and were not excluded based on internal SNP QC.
3. NRARE: the number of those SNPs classified as rare (when using the --burden option).
4. NPARAM: the number of relevant parameters used in the model. For the SNP-wise models this is an approximate value; for the principal components regression (raw data default) this is set to the number of principal components retained after pruning; for the multi-models this is the mean NPARAM value of the component base models.
5. N: the sample size used when analyzing that gene; can differ when analyzing SNP p-value input with variable sample size by SNP (due to missingness or differences in coverage in meta-analysis).
6. ZSTAT: the Z-value for the gene, based on its (permutation) p-value.
7. FDR.adjust: Calculated by the R program p.adjust using Benjamini-Hochberg procedure (BH).

Supplementary Data 7 Significant results from pathway/gene ontology terms enrichment analysis:

T1) Significant pathway enrichment analysis after correction for multiple testing (FDR<0.2)

Legend:

1. Length_GS: number of genes belong to the gene set.
2. CS: GAUSS identifies a subset of genes (called the core subset) within the gene set, which produce the maximum signal of association.
3. FDR.adjust : Calculated by the R.program p.adjust using Benjamini-Hochberg procedure (BH).

T2) Significant GO enrichment analysis after correction for multiple testing (FDR<0.2)

T3) Top pathway enrichment results for each trait (top 2 pathways listed)

T4) Top pathway enrichment results for each trait (top 2 pathways listed)

Supplementary Data 8 Result of Polygenic risk score analysis with different neuropsychiatric disorders/traits, using clumping + threshold (C+T) approach:

T1) Polygenic risk scores analysis with attention deficit hyperactivity disorder (ADHD) (p-values adjusted by the FDR approach for each language-related phenotype at 11 different p-value thresholds; same below)

Legend:

1. Logl_H1: log likelihood under the alternative hypothesis
2. l_remle: remle estimate for lambda.
3. l_mle: mle estimate for lambda.
4. p_wald: p-value from Wald test.

5. p_lrt: p-value from likelihood ratio test.
6. p_score: p-value from score test.

T2) Polygenic risk scores analysis with autism spectrum disorders (ASD)

T3) Polygenic risk scores analysis with Education attainment (EA)

T4) Polygenic risk scores analysis with cognitive performance (CP)

T5) Polygenic risk scores analysis with schizophrenia (SCZ)

T6) Polygenic risk scores analysis with bipolar disorder (BP)

T7) Polygenic risk scores analysis with major depressive disorder (MDD)

Supplementary Data 9 Lambda GC (genomic inflation factor) summary:
Lambda gc (λgc) for 34 traits. (SNPs with imputation quality score Rsq>0.3 are included.)

Supplementary Data 10 Summary of phenotypes studied

Supplementary Data 11 Result of Polygenic risk score analysis with Dyslexia / Literacy traits from Wang et al., using the clumping + threshold (C+T) approach:

T1) Polygenic risk scores analysis with developmental dyslexia (DD)

Legend:
1. Logl_H1: log likelihood under the alternative hypothesis
2. l_remle: remle estimate for lambda.
3. l_mle: mle estimate for lambda.
4. p_wald: p-value from Wald test.
5. p_lrt: p-value from likelihood ratio test.
6. p_score: p-value from score test.

(the phenotypes below are extracted from Wang et al.'s study)
T2) Polygenic risk scores analysis with word reading fluency (RFluency)

T3) Polygenic risk scores analysis with Chinese character reading accuracy(RAccuracy)

T4) Polygenic risk scores analysis with phonological awareness(PA)

T5) Polygenic risk scores analysis with morphological awareness (MA)

T6) Polygenic risk scores analysis with rapid automatized naming color (RANcol)

T7) Polygenic risk scores analysis with rapid automatized naming dice (RANdic)

T8) Polygenic risk scores analysis with rapid automatized naming picture (RANpic)

T9) Polygenic risk scores analysis with rapid automatized naming digit (RANdig)

Supplementary Data 12 Traits associated with genes listed in Tables 2-5 and Table S1.3, based on search of the GWAS catalog (SNPs in the relevant genes showing association at p<1e-5 are included; neuropsychiatric traits are highlighted)

Supplementary Data 13 Testing for genetic overlap with variants reported in other GWAS on Dyslexia/ reading ability, based on Simes and ACAT tests

Supplementary Data 14 Testing for genetic overlap with genes reported in other GWAS on Dyslexia/

reading ability, based on Simes and ACAT tests

Supplementary Data 15 Result of Polygenic risk score analysis with different neuropsychiatric disorders/traits, using SBayesR

T1) Polygenic risk scores analysis with attention deficit hyperactivity disorder (ADHD)

Legend:

1. Logl_H1: log likelihood under the alternative hypothesis
2. l_remle: remle estimate for lambda.
3. l_mle: mle estimate for lambda.
4. p_wald: p-value from Wald test.
5. p_lrt: p-value from likelihood ratio test.
6. p_score: p-value from score test.

T2) Polygenic risk scores analysis with autism spectrum disorders (ASD)

T3) Polygenic risk scores analysis with Education attainment (EA)

T4) Polygenic risk scores analysis with cognitive performance (CP)

T5) Polygenic risk scores analysis with schizophrenia (SCZ)

T6) Polygenic risk scores analysis with bipolar disorder (BP)

T7) Polygenic risk scores analysis with major depressive disorder (MDD)

Supplementary Data 16 Testing genetic dependence with variants reported in another GWAS of reading and language-related traits from Eising et al., based on the Hoeffding's test:

Legend:

1. Trait A: Hong Kong sample.
2. Trait B: Study by Eising et al.
3. Scaled test statistic: The test statistic rescaled for a standard null distribution (please refer to the R package "Independence" for details)
4. FDR.adjust : Calculated by the R program p.adjust using the Benjamini-Hochberg procedure (BH)

Supplementary Data 17 Result of Polygenic risk score analysis with different reading and language-related traits from Eising et al., using the clumping + threshold (C+T) approach (PRS are constructed based on the study by Eising et al.):

T1) Polygenic risk scores analysis with word reading

Legend:

1. Logl_H1: log likelihood under the alternative hypothesis
2. l_remle: remle estimate for lambda.
3. l_mle: mle estimate for lambda.
4. p_wald: p-value from Wald test.
5. p_lrt: p-value from likelihood ratio test.
6. p_score: p-value from score test.

T2) Polygenic risk scores analysis with non-word reading

T3) Polygenic risk scores analysis with spelling

T4) Polygenic risk scores analysis with phoneme awareness

T5) Polygenic risk scores analysis with non-word repetition

Supplementary Data 18 Testing correlation of effect sizes of top variants from the Hong Kong sample and those from GWAS of reading/language skills from Eising et al. (and vice versa):

T1) Results of effect size correlation of SNPs (with p<1e-5) from our Hong Kong sample and those from the GWAS of reading and language traits from Eising et al.

Legend:

1. Trait A: Hong Kong sample.
2. Trait B: Study by Eising et al.
3. pearson_estimate: Pearson correlation.
4. spearson_estimate: Spearman correlation.
5. pearson_p.adjust : FDR-adjusted p-value of Pearson correlation, calculated by the R program p.adjust using the Benjamini-Hochberg procedure (BH).
6. spearson_p.adjust : FDR-adjusted p-value of Spearman correlation, calculated by the R program p.adjust using the Benjamini-Hochberg procedure (BH).

T2) Results of effect size correlation of SNPs (with p<1e-5) from GWAS of reading and language traits from Eising et al. and those from our Hong Kong sample

Legend:

1. Trait A: Study by Eising et al.
2. Trait B: Hong Kong sample.

Others are the same as above.

Supplementary Data 19 Significant single-variant associations from GWAS (MAF>0.01):

T1) All single nucleotide polymorphisms (SNPs) that passed genome-wide significance (p<5e-8) (all results with imputation quality score Rsq>0.3 are shown)

Legend:

A2: effect allele.
MAF: minor allele frequency.
BP (position of the snp): base pair.
AvgCall: average call rate.
Rsq: imputation quality score.

T2) All genetic variants with FDR<0.1 in SNP-based analysis

T3) Independent risk loci (LD-clumped with r2 threshold 0.01, including SNPs with MAF between 0.01 and 0.05)

Legend:

S0001: Number of clumped SNPs (SNPs in LD) with p<1e-03.

Supplementary Discussions

Here we also briefly mention a few other SNPs reaching genomewide significance ($P<5e-8$) but with MAF between 0.01 and 0.05. We emphasize that SNPs with lower MAF should be viewed cautiously and require replications given our modest sample size; the discussions below are included for reference only. Regarding Chinese literacy skills, the SNP rs77868538 located in *TNR* (tenascin-R) was associated with CVD_Total and CVK_Total. *TNR* is primarily expressed in the central nervous system and plays a key role in human brain development by its involvement in axon growth and path finding [5]. Interestingly, variants in *TNR* have been reported to be associated with cognitive performance [6] [7]. In addition, a recent study [8] revealed that *TNR* showed a significant interaction (at $p<5e-8$) with total testosterone in affecting fluid intelligence in healthy adults. In addition, a recent GWAS on ADHD identified a variant in *TNR* as the top association achieving genome-wide significance [9]. There were also case reports that deletions in *TNR* were associated with intellectual disability and neurodevelopmental disorder [10] [11]. Moreover, animal studies showed that TNR-deficient mice had severe memory and coordination deficits [12].

In addition, we observed that the SNP rs182977703 was associated with COM_Norm and CDICT_Total, which lies within the gene *SHTN1* (Shootin 1). The gene is involved in the generation of internal asymmetric signals necessary for neuronal polarization [13]. Increased expression of *SHTN1* in hippocampal neurons may result in its accumulation in multiple neurites and formation of surplus axons [14]. Clinically, neurodevelopmental disorders such as intellectual disability (ID) may result from defects in neuronal polarity and migration [15] [16]. For example, the association of Shootin 1 with ID was supported by a whole-genome transcriptome analysis on ID patients [17].

Supplementary References

1. L Wong, S. W., Suk-Han Ho, C., McBride, C., Wing-Yin Chow, B. & Miu Yee Waye, M. Less is More in Hong Kong: Investigation of Biscriptal and Trilingual Development Among Chinese Twins in a (Relatively) Small City. *Twin Res. Hum. Genet.* **20**, 2016 (2021).
2. CHUNG, K. K. H. *et al.* The Hong Kong Test of Specific Learning Difficulties in Reading and Writing for Junior Secondary School Students [HKT-JS(II)]. (2012).
3. Liu, P. D. & McBride-Chang, C. What Is Morphological Awareness? Tapping Lexical Compounding Awareness in Chinese Third Graders. *J. Educ. Psychol.* **102**, 62–73 (2010).
4. Chik, P. P. man *et al.* Contribution of discourse and morphosyntax skills to reading comprehension in Chinese dyslexic and typically developing children. *Ann. Dyslexia* **62**, 1 (2012).
5. Leprini, A. *et al.* The Human Tenascin-R Gene *. *J. Biol. Chem.* **271**, 31251–31254 (1996).
6. Davies, G. *et al.* Study of 300,486 individuals identifies 148 independent genetic loci influencing general cognitive function. *Nat. Commun.* **9**, (2018).
7. Lee, J. J. *et al.* Gene discovery and polygenic prediction from a genome-wide association study of educational attainment in 1.1 million individuals. *Nat. Genet.* **50**, 1112–1121 (2018).
8. Liang, X. *et al.* Evaluating the genetic effects of sex hormone traits on the development of mental traits: a polygenic score analysis and gene-environment-wide interaction study in UK Biobank cohort. *Mol. Brain* **14**, (2021).
9. Hawi, Z. *et al.* A case–control genome-wide association study of ADHD discovers a novel association with the tenascin R (TNR) gene. *Transl. Psychiatry 2018 81* **8**, 1–8 (2018).
10. Dufresne, D. *et al.* Homozygous deletion of Tenascin-R in a patient with intellectual disability. *J. Med. Genet.* **49**, 451–454 (2012).
11. Wagner, M. *et al.* Loss of TNR causes a nonprogressive neurodevelopmental disorder with spasticity and transient opisthotonus. *Genet. Med. 2020 226* **22**, 1061–1068 (2020).
12. Montag-Sallaz, M. & Montag, D. Severe cognitive and motor coordination deficits </br>in Tenascin-R-deficient mice. *Genes, Brain Behav.* **2**, 20–31 (2003).
13. Toriyama, M. *et al.* Shootin1: a protein involved in the organization of an asymmetric signal for neuronal polarization. *J. Cell Biol.* **175**, 147 (2006).
14. Qiu, J. Shootin 1 for the axon. *Nat. Rev. Neurosci. 2006 712* **7**, 906–906 (2006).
15. de la Torre-Ubieta, L. & Bonni, A. Transcriptional Regulation of Neuronal Polarity and Morphogenesis in the Mammalian Brain. *Neuron* **72**, 22–40 (2011).
16. Hakanen, J., Ruiz-Reig, N. & Tissir, F. Linking cell polarity to cortical development and malformations. *Front. Cell. Neurosci.* **13**, 244 (2019).
17. InanlooRahatloo, K., Peymani, F., Kahrizi, K. & Najmabadi, H. Whole-Transcriptome Analysis Reveals Dysregulation of Actin-Cytoskeleton Pathway in Intellectual Disability Patients. *Neuroscience* **404**, 423–444 (2019).

Supplementary Figures

Supplementary Figure 1

A correlation matrix that displays the correlation coefficients for the 34 phenotypes under study.

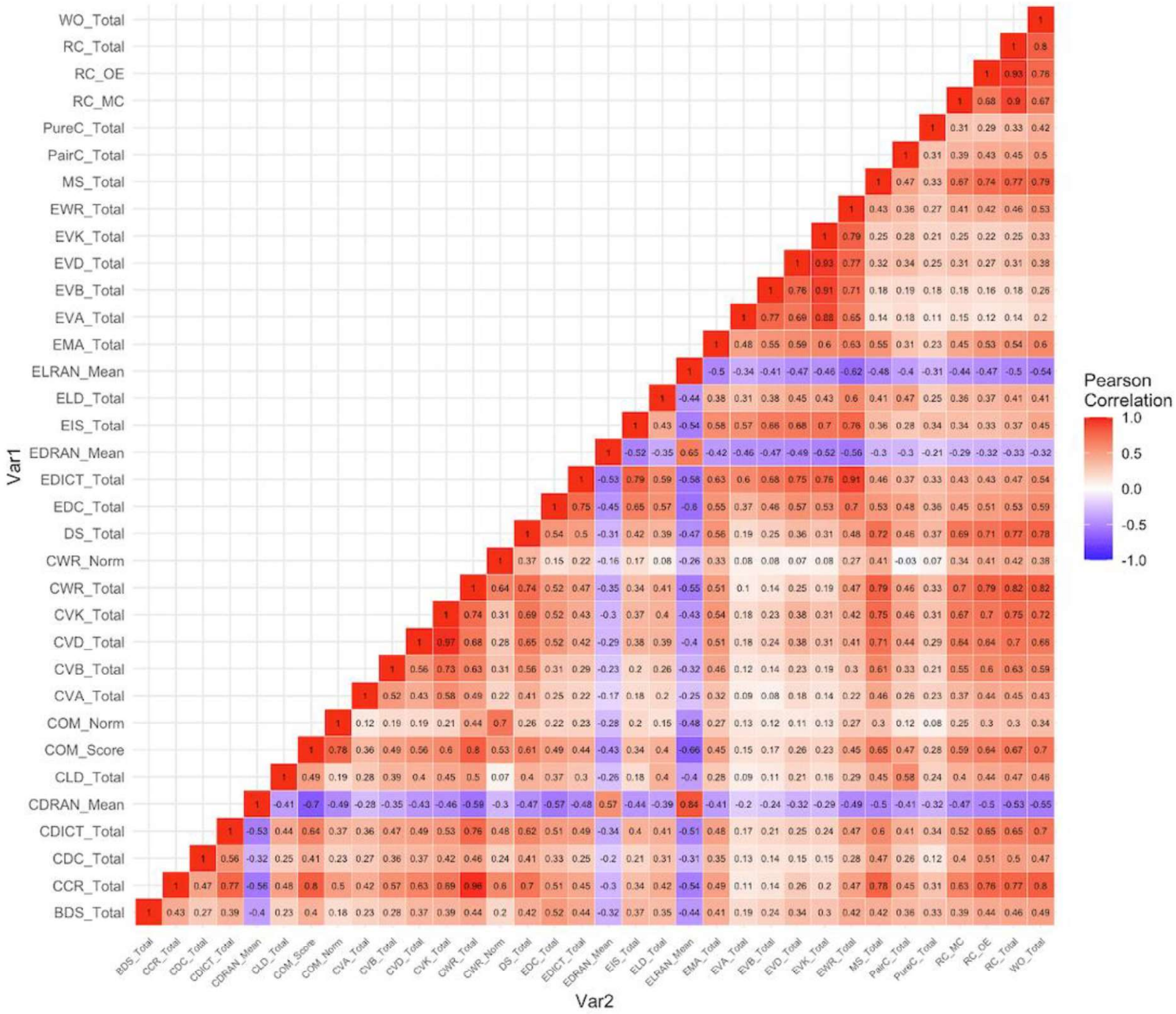

Supplementary Figure 2
The distributions of all phenotypes (without rank-based inverse normal transformation).

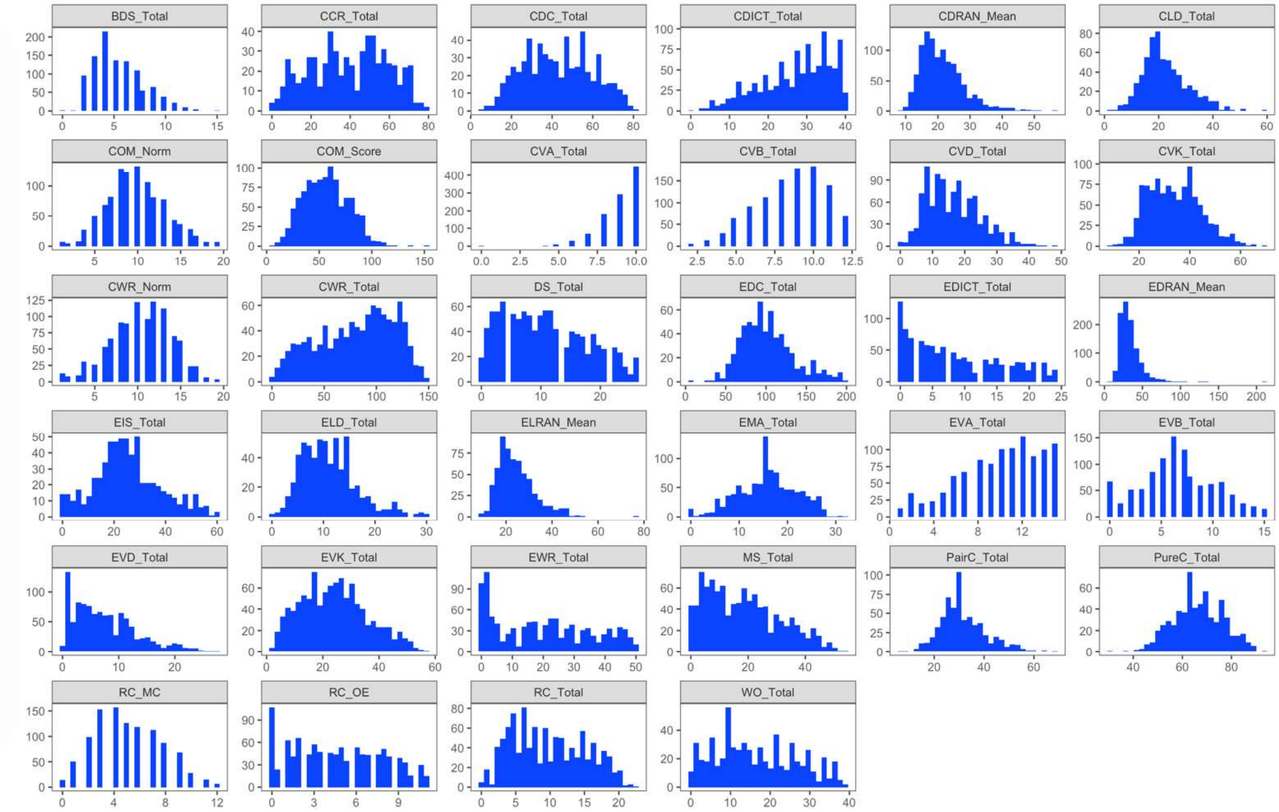

Supplementary Figure 3
Comparison of Quantile-Quantile plots for the rank-transformed and untransformed versions of BDS_Total, CVA_Total, CDRAN_Mean, and EDRAN_Mean (lambda refers to the genomic inflation factor).

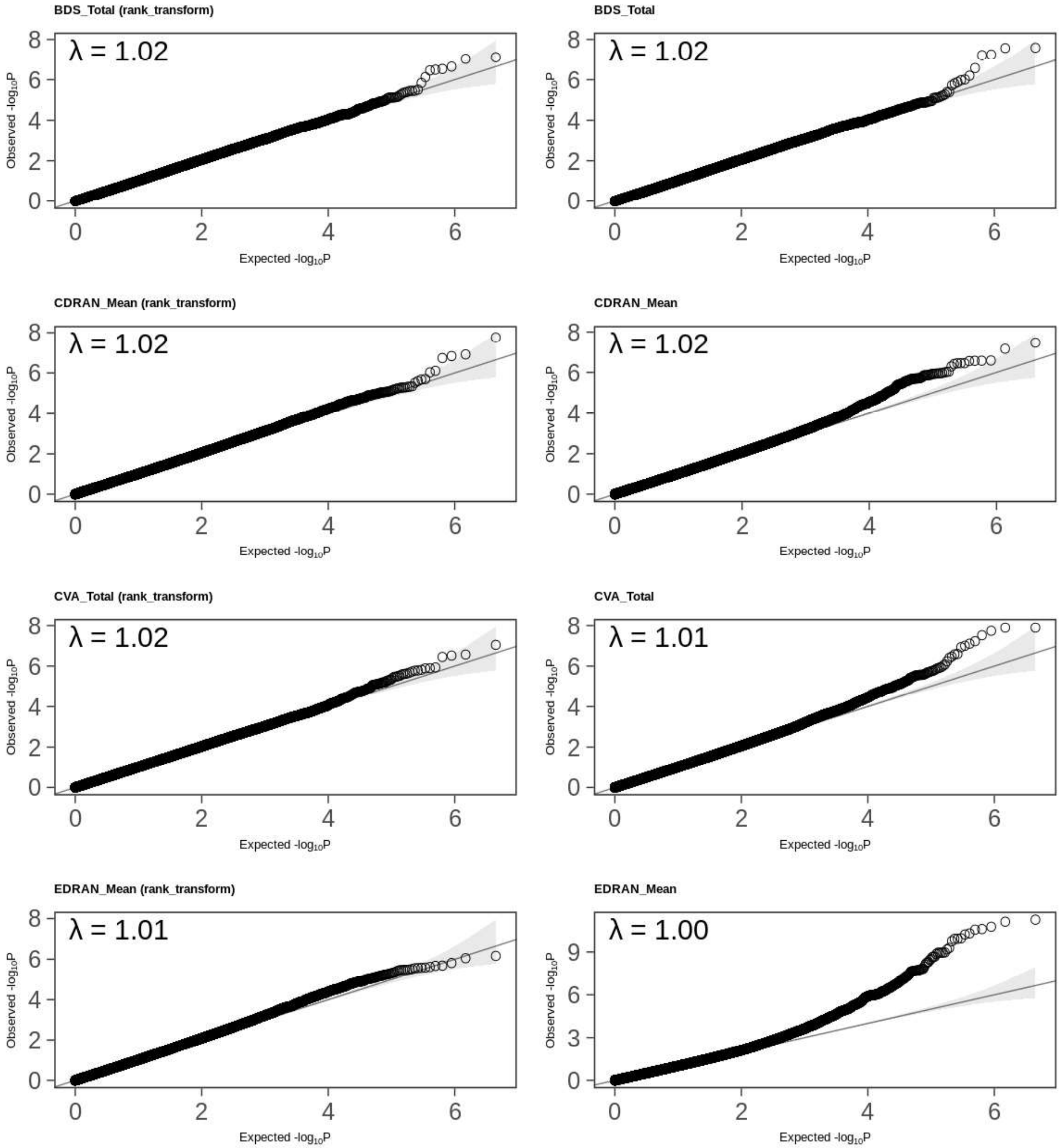

Supplementary Figure 4

Quantile-Quantile plots for the rank-transformed of BDS_Total, CVA_Total, CDRAN_Mean, and EDRAN_Mean based on gene-based test results using MAGMA.

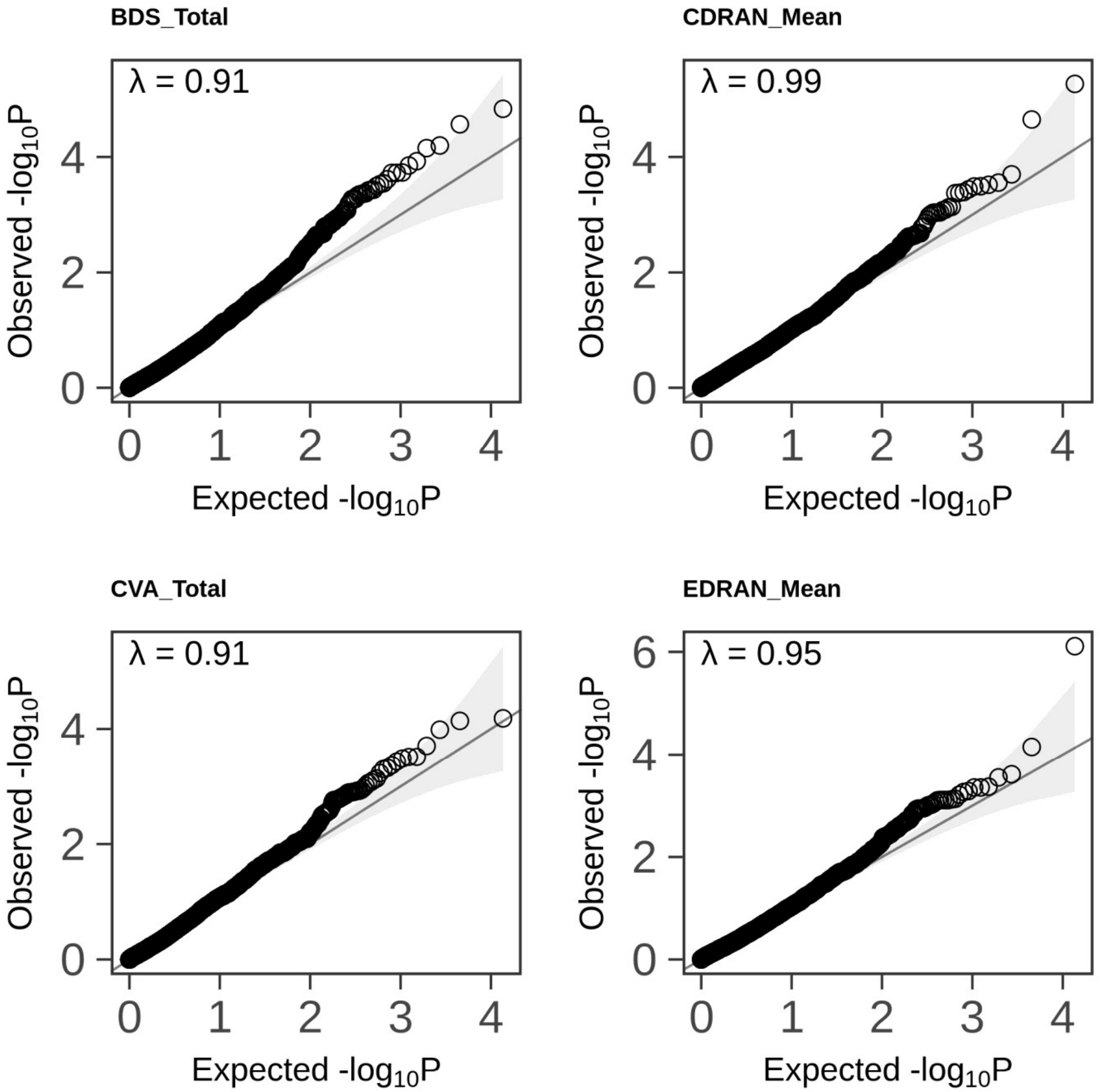

Supplementary Figure 5
Quantile-Quantile plots for other traits (un-transformed) based on gene-based test results using MAGMA.

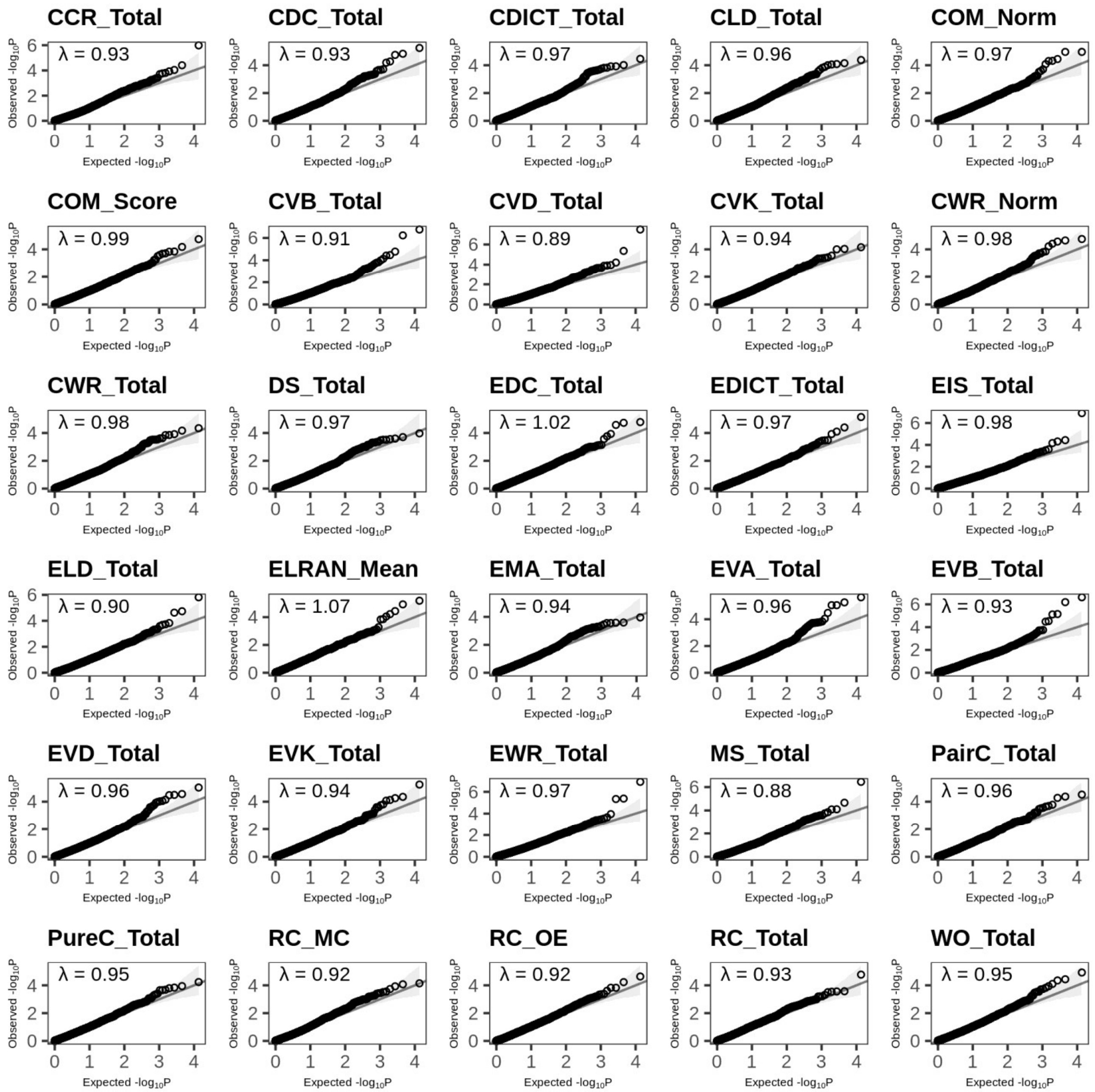

Supplementary Figure 6
Result of Polygenic risk score analysis with different reading and language-related traits from Eising et al., using SBayesR.

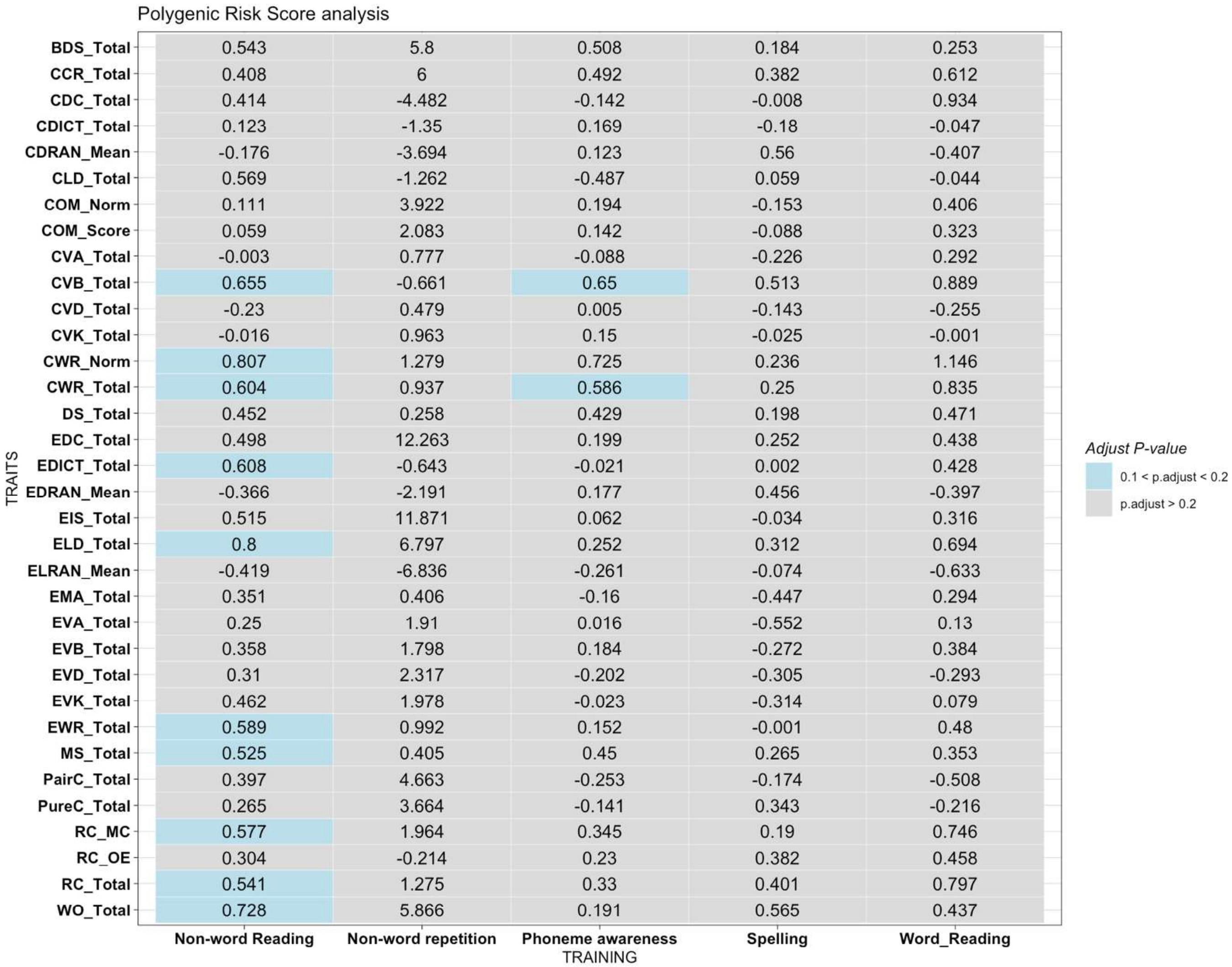

Supplement: Supplementary file 20 — Supplemental Material File #1 [file 41539_2024_229_MOESM20_ESM.pdf]
