# Supplementary material for: A genome-wide association study of Chinese and English language phenotypes in Hong Kong Chinese children
Source: NPJ Sci Learn. 2024 Mar 27;9:26. doi: 10.1038/s41539-024-00229-7 (PMC10973362; doi:10.1038/s41539-024-00229-7)
Supplement: Supplementary file 21 — reporting summary [file 41539_2024_229_MOESM21_ESM.pdf]

nature portfolio

Corresponding author(s): Hon-Cheong SO

Last updated by author(s): May 25, 2023

Reporting Summary

Nature Portfolio wishes to improve the reproducibility of the work that we publish. This form provides structure for consistency and transparency in reporting. For further information on Nature Portfolio policies, see our Editorial Policies and the Editorial Policy Checklist.

Statistics

For all statistical analyses, confirm that the following items are present in the figure legend, table legend, main text, or Methods section.

n/a	Confirmed	
☒	☐	The exact sample size (*n*) for each experimental group/condition, given as a discrete number and unit of measurement
☒	☐	A statement on whether measurements were taken from distinct samples or whether the same sample was measured repeatedly
☐	☒	The statistical test(s) used AND whether they are one- or two-sided<br>*Only common tests should be described solely by name; describe more complex techniques in the Methods section.*
☐	☒	A description of all covariates tested
☐	☒	A description of any assumptions or corrections, such as tests of normality and adjustment for multiple comparisons
☐	☒	A full description of the statistical parameters including central tendency (e.g. means) or other basic estimates (e.g. regression coefficient) AND variation (e.g. standard deviation) or associated estimates of uncertainty (e.g. confidence intervals)
☐	☒	For null hypothesis testing, the test statistic (e.g. *F*, *t*, *r*) with confidence intervals, effect sizes, degrees of freedom and *P* value noted<br>*Give P values as exact values whenever suitable.*
☒	☐	For Bayesian analysis, information on the choice of priors and Markov chain Monte Carlo settings
☒	☐	For hierarchical and complex designs, identification of the appropriate level for tests and full reporting of outcomes
☒	☐	Estimates of effect sizes (e.g. Cohen's *d*, Pearson's *r*), indicating how they were calculated

Our web collection on statistics for biologists contains articles on many of the points above.

Software and code

Policy information about availability of computer code

Data collection	no software used
Data analysis	1. GEMMA<br>2. MAGMA<br>3. MetaXcan (S-PrediXcan, S-MultiXcan)<br>4. GAUSS: Gene-set Association analysis Using Sparse Signals<br>5. Plink (Version 1.9)<br>6. SbayesR

For manuscripts utilizing custom algorithms or software that are central to the research but not yet described in published literature, software must be made available to editors and reviewers. We strongly encourage code deposition in a community repository (e.g. GitHub). See the Nature Portfolio guidelines for submitting code & software for further information.

Data

Policy information about availability of data

All manuscripts must include a data availability statement. This statement should provide the following information, where applicable:

- Accession codes, unique identifiers, or web links for publicly available datasets
- A description of any restrictions on data availability
- For clinical datasets or third party data, please ensure that the statement adheres to our policy

GWAS summary statistics of other neuropsychiatric disorders/traits were downloaded from the Social Science Genetic Association Consortium (SSGAC) (https://www.thessgac.org/), Psychiatric Genomics Consortium (PGC) (https://www.med.unc.edu/pgc) and The Integrative Psychiatric Research project (iPSYCH) (https://ipsych.au.dk/downloads/). Data of the top 10,000 associated SNPs from the GWAS on dyslexia was downloaded from https://doi.org/10.7488/ds/3465.
Summary statistics of the most significant SNPs, genes and pathways (across all phenotypes) of the current study are available in supplementary tables. For further summary statistics supporting the findings of this study, please kindly make a request to the corresponding author. Individual-level data are not available due to confidentiality concerns.

Research involving human participants, their data, or biological material

Policy information about studies with human participants or human data. See also policy information about sex, gender (identity/presentation), and sexual orientation and race, ethnicity and racism.

Reporting on sex and gender	Distribution of the gender/sex of participants are clearly described in the manuscript. The genetic findings are likely applicable to both sexes. Due to relatively modest sample size of this study, we did not perform stratified analysis in males and females.
Reporting on race, ethnicity, or other socially relevant groupings	Ethnicity is based on self-report and is reported in the manuscript (Chinese ancestry).
Population characteristics	The participants were Hong Kong Chinese-English bilingual twins and singletons, recruited through kindergarten and primary schools in Hong Kong. All children were typically developing with Cantonese as their mother language and English as their second language. The participants' ages ranged between 5 to 12 years old at the time of assessment. A total of 1048 children were recruited for this study, including 274 MZ subjects (137 pairs), 350 DZ subjects (175 pairs) and 424 singletons.
Recruitment	Children were recruited through kindergarten and primary schools in Hong Kong. A total of 1048 children were recruited for this study, including 274 MZ subjects (137 pairs), 350 DZ subjects (175 pairs) and 424 singletons. Singleton children were selected from the same schools as those twin pairs. Details of twins collection were also described in a previous paper by Wong et al. (https://doi.org/10.1017/thg.2016.90.). The children came from different schools (government-run, government-aided, private and international schools) located in different districts of Hong Kong. All children were typically developing twins with Cantonese as their mother language and English as their second language. We do not anticipate major bias in the recruitment as recruitment was performed across all districts and all types of school in Hong Kong.
Ethics oversight	This study has received ethics approval from The Joint Chinese University of Hong Kong – New Territories East Cluster Clinical Research Ethics Committee (The Joint CUHK-NTEC CREC) (reference no: 2017.479)

Note that full information on the approval of the study protocol must also be provided in the manuscript.

Field-specific reporting

Please select the one below that is the best fit for your research. If you are not sure, read the appropriate sections before making your selection.

☐ Life sciences ☒ Behavioural & social sciences ☐ Ecological, evolutionary & environmental sciences

For a reference copy of the document with all sections, see nature.com/documents/nr-reporting-summary-flat.pdf

Behavioural & social sciences study design

All studies must disclose on these points even when the disclosure is negative.

Study description	GWAS study to identify the association between genetic variant and reading/language phenotypes. The phenotypes studied are quantitative.
Research sample	The participants were Hong Kong Chinese-English bilingual twins and singletons, with Chinese (Cantonese) as their native language. Children were recruited through kindergarten and primary schools in Hong Kong. The participants' age ranged between 5 to 12 years old at the time of assessment.
Sampling strategy	Children were recruited through kindergarten and primary schools in Hong Kong. A total of 1048 children were recruited for this study, including 274 MZ subjects (137 pairs), 350 DZ subjects (175 pairs) and 424 singletons. Zygosity determination on twin pairs

	was based on the genotyped small tandem repeat (STR) markers using Quantitative Fluorescence Polymerase Chain Reaction (QF-PCR) . Singleton children were selected from the same schools as those twin pairs. Details of twins collection were also described in a previous paper by Wong et al. (https://doi.org/10.1017/thg.2016.90.).
Data collection	Parental written consent for all the participants was obtained before testing. Children completed a series of cognitive and literacy-related tasks in Chinese and English either in a laboratory setting, their school, or their home by trained research assistants. Singleton children were selected from the same schools as those twin pairs.
Timing	Subject recruitment starts from ~2014 and is an ongoing effort. The current study involves samples/phenotypes collected up to 2022.
Data exclusions	Data exclusion was only based on genotyping quality. The quality control procedure for GWAS is described in the manuscript and follows the standard practice in the field. To summarize, quality control (QC) was performed by PLINK-1.9 on each dataset separately before merging. We removed those SNPs which deviated from Hardy–Weinberg equilibrium (HWE, P < 1E-5), with Minor Allele Frequency (MAF) < 1%, missingness per individual (MIND) > 10%, and missingness per marker (GENO) > 10%. After QC, 911178 SNPs and 1046 individuals were kept for further analysis, including 274 MZ subjects (59 wmale pairs, 78 female pairs), 349 DZ subjects (39 male pairs, 37 female pairs, 1 member of a female pair and 98 opposite-sex pairs), as well as 423 singletons (218 males, 205 females).
Non-participation	No dropouts were recorded among the recruited participants (note that the assessments are performed at a single time-point, i.e. this is not a longitudinal study).
Randomization	This is not a randomized trial so participants were not randomized. Possible confounders such as age and sex are controlled for; population stratification and family relatedness are accounted for by linear mixed models.

Reporting for specific materials, systems and methods

We require information from authors about some types of materials, experimental systems and methods used in many studies. Here, indicate whether each material, system or method listed is relevant to your study. If you are not sure if a list item applies to your research, read the appropriate section before selecting a response.

Materials & experimental systems

n/a	Involved in the study
☒	☐ Antibodies
☒	☐ Eukaryotic cell lines
☒	☐ Palaeontology and archaeology
☒	☐ Animals and other organisms
☒	☐ Clinical data
☒	☐ Dual use research of concern
☒	☐ Plants

Methods

n/a	Involved in the study
☒	☐ ChIP-seq
☒	☐ Flow cytometry
☒	☐ MRI-based neuroimaging
